# Towards biological characters of interactions between transcription factors and their DNA targets in mammals

**DOI:** 10.1186/1471-2164-13-388

**Published:** 2012-08-13

**Authors:** Guangyong Zheng, Qi Liu, Guohui Ding, Chaochun Wei, Yixue Li

**Affiliations:** 1 Key Laboratory of Systems Biology, Shanghai Institutes for Biological Sciences, Chinese Academy of Sciences, 320 Yueyang Road, Shanghai, 200031, China; 2 Shanghai Center for Bioinformation Technology, 100 Qinzhou Road, Shanghai, 200235, China; 3 School of Life Sciences and Biotechnology, Shanghai Jiao Tong University, 800 Dongchuan Road, Shanghai, 200240, China

## Abstract

**Background:**

In post-genomic era, the study of transcriptional regulation is pivotal to decode genetic information. Transcription factors (TFs) are central proteins for transcriptional regulation, and interactions between TFs and their DNA targets (TFBSs) are important for downstream genes’ expression. However, the lack of knowledge about interactions between TFs and TFBSs is still baffling people to investigate the mechanism of transcription.

**Results:**

To expand the knowledge about interactions between TFs and TFBSs, three biological features (sequence feature, structure feature, and evolution feature) were utilized to build TFBS identification models for studying binding preference between TFs and their DNA targets in mammals. Results show that each feature does have fairly well performance to capture TFBSs, and the hybrid model combined all three features is more robust for TFBS identification. Subsequently, correspondence between TFs and their TFBSs was investigated to explore interactions among them in mammals. Results indicate that TFs and TFBSs are reciprocal in sequence, structure, and evolution level.

**Conclusions:**

Our work demonstrates that, to some extent, TFs and TFBSs have developed a coevolutionary relationship in order to keep their physical binding and maintain their regulatory functions. In summary, our work will help understand transcriptional regulation and interpret binding mechanism between proteins and DNAs.

## Background

Transcription factors (TFs) are important functional proteins, which play central roles in transcriptional regulation by interacting with specific DNA targets. These targets are named as transcription factor binding sites (TFBSs), which are short DNA fragments mainly located in promoter regions of genes. Generally, TFs can be grouped into four classes according to their structures and functions:(1) TFs with basic domains (basic-TFs), (2) TFs with zinc-coordinating DNA binding domains (zinc-TFs), (3) TFs with helix-turn-helix patterns (helix-TFs), and (4) beta-scaffold factors with minor groove contacts (beta-TFs)[1,2].

Interactions between TFs and their targets are significantly correlated with gene expression, so comprehensively investigating those interactions is crucial to understand transcriptional regulation. For this purpose, one of the primary steps is to represent TFBSs with appropriate features. Generally, three features are often utilized to describe biological characters of TFs’ DNA targets. (1) Sequence feature, which is the sequence similarity of DNA segments to a position weight matrix (PWM). A PWM is a mathematical model, which reflects nucleotide occurrence probability in each position [3,4]. When a DNA segment is marked with a high score to a valid PWM, it is considered as a positive instance. TFBS prediction methods based on PWM were successfully carried out on some TF data sets [3-6]. But these methods require prior PWM models, which are not available for many TFs. Besides, PWM-based methods may generate too many false positive predictions when they are executed on a genome-wide scale [7,8]. (2) Structure feature, which is conformational and physicochemical information of a DNA segment. Since transcription factors interact physically with their DNA targets, it is reasonable to depict binding preference between TFs and TFBSs through conformational and physicochemical information. For example, Pomomarenko and his colleagues [9,10] employed the conformational and physicochemical values of DNA segments to predict TFBSs. (3) Evolution feature, which is a conservation score of a DNA segment. Because transcription factor binding sites are functional elements. It is commonly believed that these elements are conserved in evolution. In fact, some algorithms for TFBS identification have been proposed based on the assumption that TFBSs are more converved than their surrounding non-functional fragments in order to maintain their functions[11-14].

Pioneer works based on the three features provide promising results and broaden our knowledge of interactions between TFs and TFBSs. Nevertheless, some aspects about interactions between TFs and TFBSs are still unclear. (1) Which feature has the greatest power for describing binding preference between TFs and TFBSs? That is to say, among the models using these three features, which one has the best performance for recognition of TFBSs? In addition, do any complementarities exist for those features? If the answer of the last question was true, then a hybrid model combining these three features should represent binding preference between TFs and TFBSs more comprehensively. (2) In terms of relationships between TFs and TFBSs, is there any correspondence existing in the sequence, structure, and evolution level? Since each of the sequence, structure, and evolution feature can denote TFBSs effectively, we can investigate the correlation between TFs and TFBSs at these three features’ aspects. To be more specific, if the sequences of two TFs are similar, will their TFBSs’ sequences be similar as well? If two TFs can be categorized into a group based on their structure information, will their corresponding TFBSs be also categorized into a group as well? If a TF is conserved in evolution, will its TFBSs be conserved as well? Answers to these questions may help people understand interactions between TFs and TFBSs and reveal their correlations in evolution.

In this paper, experimentally verified TFs and their corresponding TFBSs were first collected for three mammals (*Homo sapiens*, *Mus musculus*, and *Rattus norvegicus*), and then a TFBS recognition model was constructed based on each feature mentioned above. In total, we had three models. The accuracy of each model was used as the measurement to inspect its capability to describe binding preference between TFs and TFBSs. In addition, a hybrid model, integrating all three features, was built to evaluate complementarities of those features. After that, the correspondence between TFs and TFBSs was surveyed at sequence, structure, and evolution aspect respectively. Our results may offer new clues for TFBSs’ identification. Moreover, the correspondence between TFs and TFBSs we obtained accumulates the knowledge of interactions between proteins and DNAs. Thus, our investigation will shed light on understanding transcriptional regulation in mammals.

## Methods

### Dataset of transcription factors and their DNA targets

Experimentally verified TFs and their corresponding TFBSs were collected from the TRANSFAC database (v 9.4) [1,2] for three mammals (Human, Mouse, and Rat). A TF was selected when it contained more than 10 verified DNA targets. As a result, 326 groups of TFs and their DNA targets (TF-TFBSs) were generated. 309 of the 326 groups contained PWM patterns and the remaining 17 groups had no PWM information [see Additional file 1. The 309 groups with PWM patterns were named dataset 1, while the rest 17 groups were termed dataset 2. Moreover, according to the description of TRANSFAC database, TFs contained in the dataset 2 had less conserved binding sites, since their TFBSs were not able to be aligned to generate a PWM. Based on our previous work [15,16], among those 326 TFs, 270 TFs with amino acid sequence were classified into four classes according to their structures and domains [see Additional file 2 and Additional file 3. Detailed information of TF-TFBS datasets was summarized in Table 1. Given a TF, verified DNA targets were used as positive instances. Meanwhile, promoter sequences of the three mammals were obtained from the Eukaryotic Promoter Database (EPD) [17,18] to construct negative instances: First, those promoter sequences were utilized as training data to generate a 3^rd^-order hidden markov model; then the model was employed to produce 5 kb-long pseudo DNA sequences, which had the same nucleotide distribution of those promoter sequences; subsequently, a window (with the average length of positive instances for a TF) was employed to scan and cut those pseudo sequences for building a negative instance pool; finally, for each TF, 10 DNA sequence sets were constructed by mixing equal positive and negative instances. In practice, for each DNA sequence set, the negative instances were randomly selected from the pool.

**Table 1:** Detailed information of transcription factors and their DNA targets

**Dataset**		**Number**	**Dataset**	**Number**
TF with sequence(270)	basic-TF	56	TF-TFBSs with PWM	309
	zinc-TF	79		
	helix-TF	93	TF-TFBSs without PWM	17
	beta-TF	42		
TF without sequence(56)		56		
Total		326	Total	326

### Sequence feature of a DNA segment

For a DNA segment, its sequence feature was calculated through Equation 1 modified from some previous studies [3,5,6]. The sequence feature presented a score for assessing the similarity of a short DNA fragment to a known PWM pattern.

(1) $$\begin{array}{l} \text{score}=\sum_{j=1}^{n}W_{j(i_{j})}C_{j}\;\\ W_{j(i_{j})}=\left\{ \begin{array}{l} f_{A_{j}}\;i_{j}=A\;\\ f_{T_{j}}\;i_{j}=T\\ f_{C_{j}\;}\;i_{j}=C\\ f_{G_{j}}\;i_{j}=G \end{array}\right.\;C_{j}=\sum_{i=A}^{T}f_{i_{j}}\log_{2}(\frac{f_{i_{j}}}{P_{i}}) \end{array}$$

where n is the length of the DNA segment, j denotes a position in the DNA segment or the PWM, *i*_*j*_ denotes the base (A,T,C,G) of position j, *W*_*j*(*ij*)_ is the weight of position j for the DNA segment, *C*_*j*_ is the information content of position j for the DNA segment, *f*_*ij*_ is the frequency of base i occurred in position j for the PWM pattern, *P*_*i*_ is the observation probability of base i in background sequences. When an instance was evaluated, scores of the Watson and Crick strands were calculated respectively, and the higher one was assigned to the instance.

### Structure feature of a DNA segment

For a DNA segment, its structure feature was calculated through an empirical formula (Equation 2) proposed by Ponomarenko and his colleagues [9,10].

(2) $$\text{score}=\frac{1}{n-1}\sum_{j=1}^{n-1}x(b_{j}b_{j+1})$$

where n is the length of the DNA segment, j denotes a position of the segment, x(b_j_b_j+1_) are empirical values of 16 binucleotides combination at position j/j + 1 for transcription factor binding sites. For each conformational and physicochemical attribute, its x(b_j_b_j+1_) values were listed in Additional file 4. Based on Equation 2, for a DNA segment, a structure feature vector was built to represent the TFBS from 38 conformational and physicochemical attributes. Detailed information of these 38 attributes was provided in Additional file 4.

### Evolution feature of a DNA segment

In 2005, Xie and his colleagues [19] presented 174 conserved regulatory motifs [see Additional file 5 through alignment of several mammalian genomes. In our work, the evolution feature of a DNA segment was generated through comparing to those motifs. In practice, a conservation score of a motif was assigned to a DNA segment when it was similar to the motif (with a similarity threshold 0.95). If a DNA segment was similar to several motifs, the maximal conservation score of those motifs was assigned to the segment. If a segment was not similar to any motif, 0 was assigned to the segment (Equation 3).

(3) $$\text{evolution}\;\text{feature}=\{\begin{array}{l} 0\\ \text{the}\;\text{maximal}\;\text{conservation}\;\text{score}\;\text{of}\;\text{similar}\;\text{motifs} \end{array}$$

### Construction of the sequence model, the structure model, the evolution model, and the control model

Given a TF and its 10 DNA target sets (each set included positive and negative instances), first, three scores were calculated for each instance according to the three features. Then three TFBS identification models (named the sequence model, the structure model, and the evolution model) were constructed respectively based on these three features. In practice, the C4.5 algorithm [20,21] was utilized to build those TFBS identification models, in which the positive and negative instances with feature information were used as the input and a decision tree model was generated as the output. At the same time, the Match 2.0 method [22,23] was utilized as the control model, since it was adopted by the TRANSFAC database to measure the similarity of DNA segments to a PWM pattern.

### Construction of the hybrid model

After using the sequence, the structure, and the evolution feature separately to establish TFBS identification models, an integrated strategy was employed to inspect the complementarities of the three features. First, scores were calculated for each feature. As a result, each instance in a DNA target set was presented with 40 attributes, in which 2 attributes depicted the sequence and evolution feature respectively, and the other 38 attributes stood for the structure feature. In practice, we first combined the 40 attributes of the sequence, structure, and evolution feature, and then delivered positive and negative instances with 40 attributes to the C4.5 algorithm [20,21]. Wherein, attribute selection was carried out to remove redundant attributes using a correlation-based filter method with default parameters [24]. At last, a decision tree model, contained the three features, was constructed.

### Evaluation of different models

Given a TF, 5 models (the control model, the sequence model, the structure model, the evolution model, and the hybrid model) were built for each DNA instance set of this TF separately. In practice, a 10-fold cross validation test was used to assess the performance of each model. The test was operated as follows: (1) split an instance set into 10 fractions; (2) selected one as the test set and made the remaining 9 fractions as the training sets; (3) computed the following four statistical measurements for the subsequent analysis: (a) the true positive (TP), (b) the false positive (FP), (c) the true negative (TN), and (d) the false negative (FN). The true positive and the true negative were the correct recognition of TFBSs and non-TFBS items respectively. A false positive occurred when a non-TFBS item was predicted as a TFBS one. Similarly, a false negative occurred when a TFBS item was predicted as a non-TFBS one; (4) calculated the sensitivity, specificity, and accuracy through Equation 4; (5) repeated step (2), (3), and (4), while each fraction was chosen as the test set in turn.

(4) $$\left\{ \begin{array}{l} \text{sensitivity}=\frac{\text{TP}}{\text{TP}+\text{FN}}\\ \text{specificity}=\frac{\text{TN}}{\text{TN}+\text{FP}}\\ \text{accuracy}=\frac{\text{TP}+\text{TN}}{\text{TP}+\text{TN}+\text{FP}+\text{FN}} \end{array}\right.$$

After that, in order to further evaluate the performance of models, the receiver operating characteristic curves were constructed for the 5 different models, and the area under curve (AUC) was used as a statistic measurement to assess the power of each model to distinguish TFBSs.

## Results

### Performances of different models

**Figure 1:**
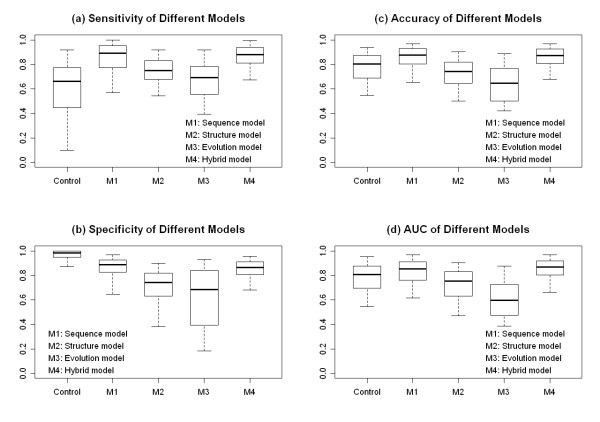
**Performance comparison of different models for 309 TF-TFBSs (with PWM information).** Panel(**a**)-(**d**): boxplots of 5 models (the control model, the sequence model, the structure model, the evolution model, and the hybrid model) for sensitivity, specificity, accuracy and AUC measurement. For a boxplot, the 5 whiskers from bottom to top denote the 5^th^, 25^th^, 50^th^, 75^th^, and 95^th^ percentile respectively.

**Figure 2:**
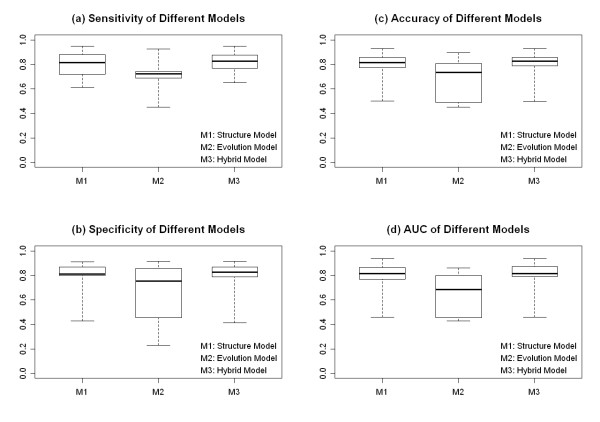
**Performance comparison of different models for 17 TF-TFBSs (without PWM information).** Panel(**a**)-(**d**): boxplot of 3 models (the structure model, the evolution model, and the hybrid model) for sensitivity, specificity, accuracy and AUC measurement. For a boxplot, the 5 whiskers from bottom to top denote the 5^th^, 25^th^, 50^th^, 75^th^, and 95^th^ percentile respectively.

10-fold cross validation tests were executed for each TF-TFBS model in dataset 1(with PWM) and dataset 2 (without PWM). Detailed results of the 10-fold cross validation test were included in the Additional file 6. Since the control model and the sequence model required PWM information, performance of these two models on dataset 2 was not presented. Detailed results of AUC measurement were listed in Additional file 7. Figure 1 showed different models’ sensitivity, specificity, accuracy, and AUC distribution in dataset 1. While Figure 2 showed those distributions in dataset 2. Table 2 and 3 summarized the mean and standard deviation of model performance for dataset 1 and 2 respectively.

**Table 2:** Performance of different models for 309 TF-TFBSs (with PWM information)

	**Control Model**		**Sequence Model**		**Structure Model**		**Evolution Model**		**Hybrid Model**	
	**Mean**	**SD**	**Mean**	**SD**	**Mean**	**SD**	**Mean**	**SD**	**Mean**	**SD**
Sensitivity	0.588	0.247	0.852	0.137	0.746	0.117	0.675	0.156	0.865	0.103
Specificity	0.965	0.052	0.859	0.105	0.710	0.152	0.616	0.256	0.848	0.088
Accuracy	0.776	0.122	0.856	0.103	0.728	0.123	0.645	0.153	0.856	0.091
AUC	0.783	0.124	0.829	0.116	0.726	0.136	0.607	0.154	0.852	0.095

**Table 3:** Performance of different models for 17 TF-TFBSs (without PWM information)

	**Control Model**		**Sequence Model**		**Structure Model**		**Evolution Model**		**Hybrid Model**	
	**Mean**	**SD**	**Mean**	**SD**	**Mean**	**SD**	**Mean**	**SD**	**Mean**	**SD**
Sensitivity	NA	NA	NA	NA	0.802	0.115	0.723	0.142	0.816	0.106
Specificity	NA	NA	NA	NA	0.788	0.157	0.655	0.252	0.788	0.165
Accuracy	NA	NA	NA	NA	0.795	0.129	0.689	0.170	0.802	0.127
AUC	NA	NA	NA	NA	0.787	0.144	0.658	0.168	0.799	0.140

Results for dataset 1 were shown in Figure 1. The interval between the 25^th^ and the 75^th^ percentile was also adopted as a model performance measurement. For sensitivity, the intervals of the 5 models (the control model, the sequence model, the structure model, the evolution model, and the hybrid model) were (0.447-0.773), (0.774-0.955), (0.676-0.830), (0.556-0.786), and (0.810-0.938) respectively. For positive instances, sensitivity results demonstrated that: (1) the sequence model had the best performance among the three single feature models; (2) the hybrid model was comparable to the best single feature model (the sequence model) and better than the control model. For specificity, interval values of the 5 models were (0.950-1.000), (0.828-0.928), (0.632-0.818), (0.393-0.842), and (0.808-0.910) respectively. For negative instances, specificity results indicated that: (1) the sequence model was the best one in three single feature models; (2) the hybrid model was comparable to the best single feature model (the sequence model) and worse than the control model. The accuracy values of the 5 models were (0.690-0.873), (0.804-0.930), (0.646-0.818), (0.502-0.768), and (0.806-0.925) respectively. When both positive and negative instances were considered, the accuracy results showed that: (1) among single feature models, the sequence model outperformed the other two for TFBS recognition; (2) the hybrid model was comparable to the best single feature model (the sequence model) and surpassed the control one. For AUC measurement, corresponding values of the 5 models were (0.696-0.877), (0.760-0.913), (0.630-0.831), (0.476-0.726), and (0.804-0.919) respectively. Conclusions hinted by the accuracy measurement were reinforced by the AUC results.

Results for dataset 2 were shown in Figure 2. For sensitivity, interval values of the structure model, evolution model and hybrid model were (0.718-0.879), (0.690-0.741), and (0.771-0.877) respectively. While for specificity, accuracy, and AUC measurement, corresponding values were [(0.800-0.868),(0.455-0.857),(0.790-0.868)], [(0.775-0.857),(0.490-0.809),(0.788-0.856)], and [(0.769-0.866),(0.455-0.802),(0.791-0.872)] respectively. Results of dataset 2 implied that without PWM information: (1) the structure model was better than the evolution model for TFBS recognition; (2) performance of the hybrid model was comparable to the best single feature model (the structure model) for identifying TFBS.

In order to compare the 5 models more directly, the mean of performance was calculated. Table 2 showed the mean values of model performance in dataset 1. In terms of accuracy, when the hybrid model was compared with the control model and the three single feature models, TFBS identification success rate improved 8.0%, 0.0%, 12.8%, and 21.1% respectively. In terms of AUC, corresponding increments were 6.9%, 2.3%, 12.6%, and 24.5% respectively. Those results suggested, again, that considering both positive and negative instances, performance of the hybrid model was comparable to the best single feature model and surpassed the control one. Table 3 showed the mean values of model performance in dataset 2. When the hybrid model was compared with the structure model, the increased values of accuracy and AUC were 0.7% and 11.3% respectively. When the hybrid model was compared with the evolution model, the increase was 1.2% and 14.1% for accuracy and AUC respectively. According to the results of dataset 2, a conclusion similar to dataset 1’s was made, that the hybrid model was comparable to the best single feature model and outperformed the control one. In addition, as shown in Table 2 and 3, the standard deviation of the hybrid model was smaller than other models’ in most cases, which meant that the hybrid model was more robust and balanced than other models.

**Figure 3:**
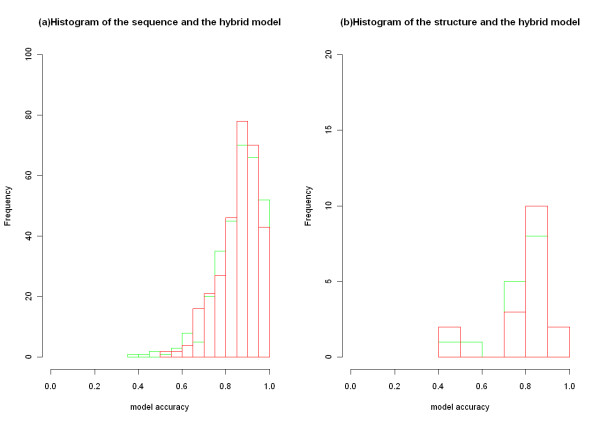
**Distribution of accuracy measurement for different models.** Panel (**a**): the histogram of the sequence model and the hybrid model. The green and red rectangle represents the former and the latter’s accuracy frequency respectively. Panel (**b**): the histogram of the structure model and the hybrid model. The green and red rectangle represents the former and the latter’s accuracy frequency respectively.

In order to survey power of the hybrid model further, we investigated frequency distribution of accuracy measurement for the hybrid model and the best single feature model in the two datasets (Figure 3). In dataset 1, the hybrid model was compared with the sequence model. While in dataset 2, the hybrid model and the structure model were compared. As shown in Figure 3, for accuracy, values of the hybrid model were more concentrated in high score region than the single feature model. That outcome demonstrated that the hybrid model was more robust than the single feature model.

### Correspondence between TFs and TFBSs

In the previous section, capability of the sequence, structure, and evolution feature to denote TFBSs were surveyed respectively through constructing TFBS identification models. In this section, biological characters of the relationship between TFs and TFBSs were investigated for better comprehending transcriptional regulation. In practice, we inspected TF-TFBS correspondence in terms of sequence, structure, and evolution to explore their relationships.

### Inspecting correspondence between TFs and TFBSs in sequence level

In sequence level, correspondence inspection was operated as follows: (1) 270 TFs (with sequences) out of 326 TFs were clustered through the BLASTCLUST algorithm [25], which could categorize sequences according to their similarity. In practice, for TF clustering, the parameter of length coverage threshold (−L) was changed from 0.60 to 0.95, with 0.05 as the step size, and the parameter of identity percentage (−S) was changed from 60 to 95, with 5 as the step size. (2) Simultaneously, corresponding TFBSs of those 270 TFs were also clustered through the BLASTCLUST algorithm, where TFBS length coverage threshold (−L) was set to 0.90 (required by the BLASTCLUST algorithm due to TFBSs’ short length), and TFBS identity percentage (−S) was changed from 60 to 95, with 5 as the step size. (3) Clustering outcomes of TFs and TFBSs were recorded separately, and then for each TFBS cluster, its items were transformed to their TF names according to TF-TFBS interaction pairs. Subsequently, matched clusters between TFs and TFBSs were checked. A TF cluster was regarded as matching with a TFBS cluster when one of below criteria held: (a) over 90% items of a TF cluster were contained in a TFBS cluster; (b) the intersection rate (Equation 5) between a TF and a TFBS cluster was over two-thirds. Results of the inspection were summarized in Table 4.

(5) $$\left\{ \begin{array}{l} \text{intersecting}\;\text{rate}=\frac{\text{intersection}\;\text{of}\;a\;\text{TF}\;\text{and}\;a\;\text{TFBS}\;\text{cluster}}{\text{union}\;\text{of}\;a\;\text{TF}\;\text{and}\;a\;\text{TFBS}\;\text{cluster}\;}\\ \text{TF}\;\text{clusters}\;\text{match}\;\text{rate}=\frac{\text{matching}\;\text{TF}\;\text{clusters}}{\text{TF}\;\text{clusters}} \end{array}\right.$$

**Table 4:** Matching results of TF and TFBS clusters based on sequence information

**BLASTCLUST (parameter)**		**clusters**		**Matching clusters**	**match rate**	**fisher test**	
**TF**	**TFBS**	**TF**	**TFBS**	**TF-TFBS**	**TF cluster**	**odds ratio**	**p value (<)**
-L 0.60 -S 60	-L 0.90 -S 60	62	233	39	0.629	123.973	2.2e-16
-L 0.65 -S 65	-L 0.90 -S 65	62	233	40	0.645	133.192	2.2e-16
-L 0.70 -S 70	-L 0.90 -S 70	60	233	40	0.667	141.485	2.2e-16
-L 0.75 -S 75	-L 0.90 -S 75	58	233	39	0.672	139.486	2.2e-16
-L 0.80 -S 80	-L 0.90 -S 80	58	233	40	0.690	151.705	2.2e-16
-L 0.85 -S 85	-L 0.90 -S 85	54	233	37	0.685	136.013	2.2e-16
-L 0.90 -S 90	-L 0.90 -S 90	43	233	27	0.628	79.789	2.2e-16
-L 0.95 -S 95	-L 0.90 -S 95	36	233	25	0.694	88.648	2.2e-16

As shown in Table 4, for TF clustering, when the length coverage threshold and identity percentage increased, cluster number dropped from 62 to 36, which meant TF clustering outcome was sensitive to these two parameters. In terms of TFBSs, when the identity percentage increased, the cluster number of TFBS was not altered. Since sequences of TFBSs were degenerated to some extent, it was not surprising that their clustering outcome was not sensitive to the sequence parameter. The match rate of TF-TFBS clusters was always over 60%, which demonstrated that most TF clusters could be found matched TFBS ones in all conditions. That is to say, to some extent, when some TFs were categorized into a cluster due to their similar sequences, their corresponding TFBSs were also classified into a cluster by sequence similarity. In another word, if some TFs’ sequences were similar, their TFBSs’ sequences were most probably similar as well. Those results suggested that to some degree, there existed correspondence in the sequence level between TFs and TFBSs.

### Inspecting correspondence between TFs and TFBSs in structure level

In structure level, correspondence inspection was executed as following: (1) 270 TFs (with sequences) out of 326 TFs were categorized into four classes (basic-TFs, zinc-TFs, helix-TFs, beta-TFs) according to their structure information [15,16]. (2) Frequency of 38 attributes for structure feature was recorded during the TFBS recognition model construction. Meanwhile, a confidence interval, based on the 75^th^ quantile of attribute frequency, was generated through a 10,000-replication bootstrapping. Then significant attributes, with frequencies over median of the interval, were selected for subsequent process. As a result, 5 (the 27^th^, 30^th^, 32^th^, 33^th^, and 34^th^ attributes) out of the 38 attributes were chosen, and TFBSs of the 270 TFs were encoded with a 5-dimension vector. (3) Expectation-Maximization (EM) algorithm was employed to evaluate class number of TFBSs, and then the number was delivered to K-means cluster algorithm as an initial parameter for TFBS classification. (4) For each TFBS class, its items were transformed to their TF names according to TF-TFBS interaction pairs. Then mapping status between TF and TFBS classes was inspected with similar criteria used in the previous section (inspecting correspondence in sequence level). In practice, mapping status was defined as Yes when over 90% items of a TF class were found in a TFBS class. The mapping results of four TF classes were summarized in Table 5.

**Table 5:** Mapping results of TF classes based on structure information

**TF class**	**items in class**	**mapped items**	**mapping rate**	**mapping status**
basic-TF	56	52	0.929	Yes
beta-TF	42	39	0.929	Yes
helix-TF	93	84	0.903	Yes
zinc-TF	79	75	0.949	Yes

As shown in Table 5, for each TF class, in terms of class-level mapping rate, the numbers were no less than 90%, which suggested that every TF class found a matched TFBS class. That is to say, according to structure information, when some TFs were grouped into a class, their corresponding TFBSs were most likely categorized into a class as well. Therefore, we thought that in structure level, correspondence between TFs and TFBSs did exist as well.

### Inspecting correspondence between TFs and TFBSs in evolution level

In evolution level, correspondence inspection was carried out as belows: (1) Homolog information of 270 TFs (with sequences) was collected from the InParanoid database, which contained eukaryotic ortholog groups [26,27]. Then each TF was assigned a conservation score based on the number of its orthologs. In practice, a TF obtained higher score when it had more orthologous genes. (2) Simultaneously, for each TF, conservation of its DNA targets was assessed through their evolution feature during model construction for TFBS identification. In practice, the mean value of evolution feature for a TF’s DNA target was assigned as its corresponding TFBSs’ conservation score. (3) Correspondence between TFs and TFBSs was inspected through surveying correlation of conservation score between TFs and their DNA targets. Detailed information about conservation score of TFs and their DNA targets was listed in Table 6.

**Table 6:** Conservation scores of 270 TFs and their corresponding TFBSs

**TF_id**	**TF_score**	**TFBS_score**	**TF_id**	**TF_score**	**TFBS_score**	**TF_id**	**TF_score**	**TFBS_score**
T00035	0.510	22.689	T02068	0.229	37.695	T01581	0.281	22.469
T00036	0.323	53.477	T02256	0.000	19.975	T01649	0.490	21.440
T00040	0.156	17.661	T02336	0.458	18.020	T01675	0.479	16.642
T00045	0.417	24.431	T02338	0.635	43.785	T01710	0.312	19.878
T00100	0.000	8.600	T02513	0.510	19.355	T01737	0.396	19.207
T00105	0.250	23.436	T02689	0.573	11.751	T01788	0.000	42.591
T00112	0.000	26.139	T02758	0.000	24.052	T01806	0.281	9.876
T00113	0.281	31.947	T02769	0.406	31.273	T01828	0.396	15.416
T00123	0.323	37.325	T02905	0.417	29.244	T01836	0.552	38.128
T00133	0.531	40.426	T03828	0.000	30.425	T01862	0.365	27.131
T00137	0.000	22.547	T03978	0.344	16.819	T01863	0.365	27.131
T00140	0.469	80.516	T04076	0.333	20.941	T01873	0.406	19.462
T00149	0.417	24.994	T04096	0.406	41.469	T01888	0.365	20.913
T00163	0.500	36.319	T04139	0.396	32.720	T01944	0.240	26.854
T00167	0.615	40.211	T04169	0.292	20.608	T01964	0.000	36.555
T00168	0.312	12.591	T04255	0.229	9.579	T02016	0.396	11.973
T00204	0.000	43.157	T04292	0.458	32.288	T02057	0.333	10.183
T00207	0.000	23.891	T04323	0.469	30.400	T02072	0.406	5.871
T00241	0.458	35.887	T04337	0.417	8.588	T02083	0.000	22.700
T00250	0.000	38.958	T04345	0.281	17.885	T02142	0.146	22.509
T00311	0.000	21.446	T04362	0.344	16.418	T02251	0.292	40.375
T00330	0.250	19.700	T04651	0.219	24.345	T02327	0.271	81.746
T00331	0.438	25.084	T04673	0.000	16.867	T02344	0.521	22.483
T00337	0.000	11.789	T04674	0.000	14.984	T02349	0.417	11.981
T00368	0.208	25.903	T04675	0.240	10.487	T02361	0.000	26.080
T00423	0.208	18.856	T04682	0.000	19.150	T02450	0.396	12.598
T00490	0.000	42.650	T04683	0.521	26.489	T02529	0.250	31.583
T00525	0.354	37.525	T04684	0.521	29.356	T02532	0.000	9.291
T00529	0.542	5.670	T04728	0.938	2.200	T02772	0.323	33.126
T00539	0.000	23.310	T04734	0.448	33.173	T02983	0.156	12.375
T00581	0.156	16.629	T04742	0.417	29.900	T03388	0.000	17.450
T00594	0.406	18.237	T05040	0.417	10.977	T03389	0.604	18.979
T00625	0.000	30.061	T05887	0.208	12.650	T03461	0.490	12.826
T00630	0.417	9.392	T05990	0.167	9.142	T04176	0.427	22.757
T00641	0.417	28.149	T06429	0.333	19.271	T04203	0.458	24.991
T00646	0.312	25.800	T08251	0.479	18.206	T04347	0.271	24.125
T00647	0.312	27.887	T08292	0.188	19.371	T04368	0.406	12.612
T00671	0.271	10.249	T08300	0.240	25.765	T04446	0.458	1.591
T00719	0.000	32.340	T00017	0.167	18.741	T04668	0.073	13.053
T00721	0.000	24.300	T00018	0.000	8.178	T04669	0.562	24.492
T00759	0.604	44.722	T00104	0.260	14.491	T04670	0.615	28.587
T00764	0.354	19.933	T00111	0.365	38.400	T04671	0.604	28.089
T00794	1.000	18.030	T00131	0.542	37.864	T04811	0.000	14.562
T00851	0.000	30.779	T00138	0.000	10.669	T04849	0.354	28.104
T00857	0.490	23.217	T00152	0.000	13.035	T05012	0.167	51.522
T00874	0.448	48.698	T00244	0.490	32.023	T05840	0.625	48.409
T00878	0.448	58.069	T00273	0.615	13.368	T05943	0.417	22.448
T00885	0.396	15.250	T00278	0.385	19.934	T06029	0.396	20.694
T00899	0.417	34.042	T00377	0.490	23.438	T06585	0.438	24.253
T00900	0.417	29.490	T00378	0.458	5.421	T06593	0.198	32.085
T00902	0.000	30.319	T00402	0.250	11.518	T08231	0.490	14.017
T00915	0.427	15.628	T00422	0.167	13.413	T08291	0.344	18.350
T00929	0.469	16.593	T00425	0.167	15.035	T00042	0.146	10.674
T00968	0.490	28.308	T00437	0.000	42.280	T00108	0.000	17.824
T00997	0.073	23.077	T00454	0.438	27.733	T00109	0.000	7.500
T01005	0.792	28.543	T00505	0.771	27.210	T00124	0.312	42.912
T01009	0.792	33.484	T00526	0.312	43.133	T00132	0.531	33.659
T01042	0.750	18.733	T00528	0.219	38.886	T00164	0.510	38.254
T01071	0.500	14.414	T00595	0.406	22.510	T00183	0.354	21.642
T01122	0.000	11.302	T00644	0.365	24.060	T00258	0.167	20.208
T01313	0.219	27.573	T00648	0.312	19.429	T00333	0.229	24.129
T01345	0.542	27.789	T00651	0.156	22.926	T00369	0.219	23.012
T01346	0.000	34.134	T00677	0.333	12.273	T00371	0.000	20.978
T01427	0.656	20.220	T00680	0.000	33.917	T00372	0.458	23.436
T01428	0.208	32.483	T00681	0.000	24.983	T00424	0.177	16.045
T01462	0.260	37.833	T00684	0.000	43.609	T00459	0.219	14.155
T01468	0.302	15.041	T00694	0.250	16.028	T00535	0.375	30.000
T01481	0.562	20.503	T00702	0.208	35.793	T00599	0.375	40.421
T01493	0.260	27.513	T00752	0.604	53.710	T00691	0.000	22.778
T01527	0.458	29.300	T00765	0.365	12.164	T00754	0.583	44.405
T01528	0.448	29.592	T00859	0.490	19.629	T00853	0.000	23.131
T01542	0.510	30.648	T00877	0.396	45.574	T00856	0.479	16.747
T01553	0.542	25.917	T00930	0.448	24.136	T01040	0.479	14.921
T01580	0.292	18.325	T00989	0.469	35.417	T01049	0.531	19.961
T01599	0.375	19.287	T01112	0.000	17.350	T01050	0.271	20.600
T01607	0.448	40.100	T01147	0.448	29.662	T01349	0.458	38.760
T01673	0.677	23.779	T01201	0.396	6.845	T01562	0.531	13.891
T01795	0.417	13.877	T01211	0.219	19.882	T01921	0.219	23.153
T01804	0.604	45.070	T01311	0.469	14.534	T02115	0.396	47.292
T01823	0.000	15.839	T01331	0.531	33.655	T02288	0.000	27.853
T01839	0.417	28.932	T01332	0.479	33.209	T02290	0.417	17.006
T01840	0.417	29.820	T01429	0.302	25.000	T02716	0.479	11.258
T01853	0.500	23.999	T01441	0.156	62.155	T02815	0.427	1.021
T01920	0.000	13.056	T01445	0.469	71.685	T03257	0.000	15.213
T01948	0.240	28.606	T01483	0.365	21.781	T03258	0.000	15.307
T01950	0.208	24.200	T01526	0.469	14.751	T03458	0.490	8.353
T01951	0.208	24.200	T01543	0.500	26.750	T04297	0.448	17.736
T01973	0.188	11.157	T01554	0.542	17.850	T04761	0.521	20.942
T01975	0.000	24.591	T01574	0.260	26.200	T05026	0.000	7.374
T02054	0.406	15.031	T01579	0.531	23.187	T05137	0.365	10.673

A spearman’s rank test was used to investigate the correlation between TFs and TFBSs. As a result, the coefficient of TFs and TFBSs was 0.122 (p = 0.023 < 0.05, one side test), which meant there was positive correlation between transcription factors and their DNA targets to some degree. Those results suggested when a TF was conserved, its TFBSs were likely conserved. In other words, in terms of evolution, there exists correspondence between TFs and their TFBSs.

## Discussion

In this work, we first evaluated the power of sequence, structure, and evolution feature to describe properties of transcription factor binding sites through constructing TFBS identification model. For TF datasets with PWM information, TFBS identification accuracy of the three single feature models achieved 86%, 73%, and 65% for the sequence, structure and evolution model respectively. Given no PWM information, accuracy of the structure and the evolution feature were about 80% and 69%. Those results demonstrate: (1) these features do have fairly well capability to capture TFBSs; (2) among the three features, the sequence feature is most impactful for depicting TFBS binding preference. It is noteworthy that prior PWM information is required when computing the sequence feature. In contrast, the structure and the evolution feature don’t need much prior information when they are applied to TFBS recognition. Thus, the structure and the evolution feature are more suitable than the sequence one for *ab inito* TFBS recognition in a certain degree.

A hybrid model was built to survey the complementarities of the three features. According to the outcomes of sensitivity, specificity, accuracy, and AUC measurement, performance of the hybrid model exceeds the control one and is comparable to the best single feature model. Moreover, the hybrid model has fairly well performance not only in TF sets having PWM information (dataset 1) but also in TF sets with low conserved TFBSs (dataset 2). Powerful capability of the hybrid model can be explained by following two reasons: (1) In terms of biological character, the sequence feature presents similarity of an DNA sequence to a PWM pattern; the structure feature contains conformational and physicochemical attributes, which are thought to be closely related to TFBS binding; the evolution feature depicts conservation degree of a DNA segment. The three features offer properties of TFBSs in various biologic aspects, so combining these features can describe TFBS binding preference more comprehensively. (2) In terms of string context, for a DNA segment, the sequence feature gives contribution of each nucleotide to a valid pattern (PWM pattern); the structure feature is correlative to dinucleotide distribution, which reflects relationship of joint nucleotides; the evolution feature considers conservation of a DNA segment as a whole. In methodology, integrated model is more effectively using string context than the single feature model, so it is not surprising that the hybrid model has better performance for TFBS recognition. In summary, investigation results illustrates: (1) there are complementarities over the three biological features to some extent; (2) strategy of combining different features is good to TFBS identification.

After investigating competence of the sequence, structure, and evolution feature to distinguish TFBSs, we investigated the correspondence in those features’ levels to explore the interaction mechanism between TFs and TFBSs. Results of correspondence inspection make clear that TFs are reciprocal with TFBSs: (1) in sequence level, when some TFs’ sequences are similar, their corresponding TFBSs’ sequences are also similar. In general, when some proteins’ sequences are similar, they are believed to have analogous functions. TFs are pivotal proteins of transcriptional regulation, and their most important functions are binding with TFBSs to regulate expression of downstream target genes. Hence, it is reasonable when some TFs having similar sequences, sequences of their TFBSs are similar as well. Those reciprocal phenomena of TFs and TFBSs in sequence level are functional reflection of interactions between them; (2) in structure level, when some TFs are grouped into a class, it is most probably that their TFBSs are categorized into a class as well. When some TFs belong to a class, they generally have analogous structure domain. It is well known that interactions between TFs and TFBSs are determined by structure domains of the former and fold conformation of the latter. When some TFs are clustered into a class, they interact with analogous TFBSs. Analogous TFBSs are usually having similar fold conformation. Therefore, it is not surprising that we can observe structure correspondence between TF and TFBS. Those results are directly mapping at structure aspect for interactions between TFs and TFBSs; (3) in evolution level, when a TF is conserved, its corresponding TFBSs are likely to have low mutation rates. In another words, TFs and their TFBSs have consistent mutation trends in evolution. Considering the opposite situation, a TF is conserved which indicates it has low mutation rate. But its TFBSs are more active and have a high mutation rate. When those TFBSs’ sequences are mutated and their fold conformations are changed. They will not be bound by the original TF, which means interactions between the TF and its DNA targets are eliminated. Thus TFs and their TFBSs should have coherent trends in evolution so as to maintain interactions between them. According to coherence between TFs and TFBSs at sequence, structure, and evolution aspect, we deem that, to a certain degree, TFs and TFBSs have co-evolved in order to keep their physical binding and maintain their regulatory functions, which is consistent with reports of Yang’s work [28].

## Conclusions

In this work, we gave an insight into biological characters of interactions between transcription factors and their DNA targets. Our results show that the sequence, structure, and evolution features do have powerful performance not only in TFBS recognition, but also in TF-TFBS interaction description. Besides, it is a reasonable strategy to combine the three features for capturing TFBSs. Furthermore, interesting finding of correspondence inspection between TFs and TFBSs makes solid contribution to transcriptional regulation: On one hand, coherence between TFs and TFBSs in sequence, structure, and evolution level gives aid to people for interpreting TFBS binding preference; On the other hand, the reciprocal phenomena of TFs and TFBSs at sequence, structure, and evolution aspect provide useful information for the research of interactions between proteins and DNAs. In summary, results of our work widen the knowledge of interactions between transcription factors and their binding sites, which will help us further investigate transcriptional regulation and explore binding mechanisms between proteins and DNAs.

## Competing interests

The authors declare that they have no competing interests.

## Authors’ contributions

GYZ collected datasets, carried out experiments, and drafted the manuscript. QL and GHD help to collect datasets. CCW and YXL directed the whole research work and revised the manuscript. All authors read and approved the manuscript.

## Supplementary Material

Additional file 1 Information of 326 transcription factors. Click here for file

Additional file 2 Sequences of 270 transcription factors. Click here for file

Additional file 3 Classification of 270 transcription factors (with sequences). Click here for file

Additional file 4 Information of 38 conformational and physicochemical attributes. Click here for file

Additional file 5 Information of 174 conservative motifs. Click here for file

Additional file 6 Results of performance inspection for dataset1 (TF-TFBS with PWM information) and dataset2 (TF-TFBS without PWM information). Click here for file

Additional file 7 Results of AUC measure for dataset1 (TF-TFBS with PWM information) and dataset2 (TF-TFBS without PWM information). Click here for file
