# Supplementary material for: Towards biological characters of interactions between transcription factors and their DNA targets in mammals
Source: BMC Genomics. 2012 Aug 13;13:388. doi: 10.1186/1471-2164-13-388 (PMC3472306; doi:10.1186/1471-2164-13-388)
Supplement: Additional file 2 — Sequences of 270 transcription factors. [file 1471-2164-13-388-S2.pdf]

>T00167
MKFKLHVNSARQYKDLWNMSDDKPFLCTAPGCGQRFTNEDHLAVHKHKHEMTLKFGPARNDSVIVADQ
TPTPTRFLKNCEEVGLFNELASPFENEFKKASEDDIKKMPLDLSPLATPIIRSKIEEPSVVETTHQDSPLPHPE
STTSDEKEVPLAQTAQPTSAIVRPASLQVPNVLLTSSDSSVIIQQAVPSPTSSTVITQAPSSNRPIVPVPGPFPL
LLHLPSGQTMPVAIPASITSSNVHVPAAVPLVRPVTMVPSVPGIPGPSSPQPVQSEAKMRLKAALTQQHPPV
TNGDTVKGHGSGLVRTQSEESRPQSLQQPATSTTETPASPAHTTPQTQSTSGRRRRAANEDPDEKRRKFLE
RNRAAASRCRQKRKVWVQSLEKKAEDLSSLNGQLQSEVTLLRNEVAQLKQLLLAHKDCPVTAMQKKSG
YHTADKDDSSEDISVPSSPHTEAIQHSSVSTSNGVSSTSKAEAVATSVLTQMADQSTEPALSQIVMAPSSQS
QPSGS
>T00968
MEDSHKSTTSETAPQPGSAVQGAHISHIAQQVSSLSESEESQDSSDSIGSSQKAHGILARRPSYRKILKDLSS
EDTRGRKGDGENSGVSAAVTSMSVPTPIYQTSSGQYIAIAPNGALQLASPGTDGVQGLQTLTMTNSGSTQ
QGTTILQYAQTSDGQQILVPSNQVVVQTASGDMQTYQIRTTPSATSLPQTVVMTSPVTLTSQTTKTDDPQL
KREIRLMKNREAARECRRKKKEYVKCLENRVAVLENQNKTLIEELKTLKDLYSNKSV
>T01071
MEKMSRPLPLNPTFIPPPYGVLRSLLENPLKLPLHHEDAFSKDKDKEKKLDDESNSPTVPQSAFLGPTLWD
KTLPYDGDTFQLEYMDLEEFLSENGIPPSPSQHDHSPHPPGLQPASSAAPSVMDLSSRASAPLHPGIPSPNC
MQSPIRPGQLLPANRNTPSPIDPDTIQVPVGYEPDPADLALSSIPGQEMFDPRKRKFSEEELKPQPMIKKAR
KVFIPDDLKDDKYWARRRKNNMAAKRSRDARRLKENQIAIRASFLEKENSALRQEVADLRKELGKCKNI
LAKYEARHGPL
>T01481
MDEQPRLMHSHAGVGMAGHPGLSQHLQDGAGGTEGEGGRKQDIGDILQQIMTITDQSLDEAQARKHAL
NCHRMKPALFNVLCEIKEKTVLSIRGAQEEEPTDPQLMRLDNMLLAEGVAGPEKGGGSAAAAAAAASG
GAGSDNSVEHSDYRAKLSQIRQIYHTELEKYEQACNEFTTHVMNLLREQSRTRPISPKEIERMVSIIHRKFS
SIQMQLKQSTCEAVMILRSRFLDARRKRRNFNKQATEILNEYFYSHLSNPYPSEEAKEELAKKCGITVSQV
SNWFGNKRIRYKKNIGKFQEEANIYAAKTAVTATNVSAHGSQANSPSTPNSAGSSSSFNMSNSGDLFMSVQ
SLNGDSYQGAQVGANVQSQVDTLRHVISQTGGYSDGLAASQMYSPQGISANGGWQDATTPSSVTSPTEG
PGSVHSDTSN
>T01607
MAAAEPASSGQQAPAGQGQGQRPPPQPPQAQAPQPPPPPQLGGAGGGSSRHEKSLGLLTTKFVSLLQEAK
DGVLDLKAAADTLAVRQKRRIYDITNVLEGIDLIEKKSKNSIQWKGVGAGCNTKEVIDRLRYLKAEIEDLE
LKERELDQQKLWLQQSIKNVMDDSINNRFSYVTHEDICNCFNGDTLLAIQAPSGTQLEVPIPEMGQNGQK
KYQINLKSHSGPIHVLLINKESSSSKPVVFPVPPPDDLTQPSSQSLTPVTPQKSSMATQNLPEQHVSERSQAL
QQTSATDISSAGSISGDIIDELMSSDVFPLLRLSPTPADDYNFNLDDNEGVCDLFDVQILNY
>T01975
MEPIDLSIPKNFRKGDKDLATPSEXKKPEEEAGSSEQPSPCPAPGPSLPVTLGPSGILESPMAPAPAATPEPPA
QPLQGPVQLAVPIYSSALVSSPPLVGSSALLSGTALLRPLRPKPPLLLPKPPVTEELPPLASIAQIISSVSSAPTL
LKTKVADPGPASTGSNTTASDSLGGSVPKAATTATPAATTSPKESSEPPAPASSPEAASPTEQGPARTSKKRG
RKRGMRSRPRANSGGVDLDSSGEFASIEKMLATTDTNKFSPFLQTAEDNTQDEVAGAPADHHGPSDEEQG
SPPEDKLLRAKRNSYTNCLQKITCPHCPRVFPWASSLQRHXLTHTDSQSDAETAAAXGEVLDLTSRDREQP
SEGATELRQVAGDAPVEQATAETASPVHREEHGRGESHEPEEEHGTEESTGDADGGRGRVEQPEPGPGLR
HQAHGLQAGGGRRRGRPGGAASQEQKLACDTCGKSFKFLGTLSRHRKAHGRQEPKDEKGDGATTAEEG
PSPAPEQEEKPPETPAEVVESAPGAGEAPAEKLAEETEGPSDGESAAEKRSSEKSDDDKKPKTDSPKSVASK
ADKRKKVCSVCNKRFWSLQDLTRHMRSHTGERPYKCQTCERTFTLKHSLVRHQRIHQKARHAKHHGKD

SDKEERGEEDSENESTHSGNNAVSENEAELAPNASNHMAVTRSRKEGLASATKDCSHREEKVTAGWPSEP
GQGDLNPESPAALGQDLLEPRSKRPAHPILATADGASQHVGME
>T02769
MDYSYDEDLDELCPVCGDKVSGYHYGLLTCESCKGFFKRTVQNNKHYTCTESQSCKIDKTQRNRCPFCR
FQKCLTVGMRLEAVRADRMRGGRNKFGPMYKRDRALKQQKKAQIRANGFKLETGPPMGVPPPPPPAPD
YVLPPSLHGPEPKGLAAGPPAGPLGDFGAPALPMAVPGAHGPLAGYLYPAFPGRAIKSEYPEPYASPPQPGL
PYGYPEPFSGGPNVPELILQLLQLEPDEDQVRARILGCLQEPTKSRPDQPAAFGLLCRMADQTFISIVDWAR
RCMVFKELEVADQMTLLQNCWSELLVFDHIYRQVQHGKEGSILLVTGQEVELTTVATQAGSLLHSLVLRA
QELVLQLLALQLDRQEFVCLKFIILFSLDLKFLNNHILVKDAQEKANAALLDYTLCHYPHCGDKFQQLLLC
LVEVRALSMQAKEYLYHKHLGNEMPRNNLLIEMLQAKQT
>T04076
MKGKKGIVAASGSETEDEDSMDIPLDLSSSAGSGKRRRRGNLPKESVQILRDWLYEHRYNAYPSEQEKAL
LSQQTHLSTLQVCNWFINARRRLLPDMLRKDGKDPNQFTISRRGAKISETSSVESVMGIKNFMPALEETPF
HSCTAGPNPTLGRPLSPKPSSPGSVLARPSVICHTTVTALKDVPFSLCQSVGVGQNTDIQQIAAKNFTDTSL
MYPEDTCKSGPSTNTQSGLFNTPPPTPPDLNQDFSGFQLLVDVALKRAAEMELQAKLTA
>T04337
MSLTNTKTGFSVKDILDLPDTNDEEGSVAEGPEEENEGPEPAKRAGPLGQGALDAVQSLPLKNPFYDSSDN
PYTRWLASTEGLQYSLHGLAAGAPPQDSSSKSPEPSADESPDNDKETPGGGGDAGKKRKKRRVLFSKAQT
YELERRFRQQRYLSAPEREHLASLIRLTPTQVKIWFQNHRYKMKRARAEKGMEVTPLPSPRRVAVPVLVR
DGKPCHALKAQDLAAATFQAGIPFSAYSAQSLQHMQYNAQYSSASTPQYPTAHPLVQAQQWTW
>T04728
MPRIMIKGGVWRNTEDEILKAAVMKYGKNQWSRIASLLHRKSAKQCKARWYEWLDPSIKKTEWSREEE
EKLLHLAKLMPTQWRTIAPIIGRTAAQCLEHYEFLLDKAAQRDNEEETTDDPRKLKPGEIDPNPETKPARP
DPIDMDEDELEMLSEARARLANTQGKKAKRKAREKQLEEARRLAALQKRRELRAAGIEIQKKRKRKRGV
DYNAEIPFEKKPALGFYDTSEENYQALDADFRKLRQQDLDGELRSEKEGRDRKKDKQHLKRKKESDLPS
AILQTSGVSEFTKKRSKLVLPAPQISDAELQEVVKVGQASEIARQTAEESGITNSASSTLLSEYNVTNNSVAL
RTPRTPASQDRILQEAQNLMALTNVDTPLKGGLNTPLHESDFSGVTPQRQVVQTPNTVLSTPFRTPSNGAE
GLTPRSGTTPKPVINSTPGRTPLRDKLNINPEDGMADYSDPSYVKQMERESREHLRLGLLGLPAPKNDFEIV
LPENAEKELEEREIDDTYIEDAADVDARKQAIRDAERVKEMKRMHKAVQKDLPRPSEVNETILRPLNVEP
PLTDLQKSEELIKKEMITMLHYDLLHHPYEPSGNKKGKTVGFGTNNSEHITYLEHNPYEKFSKEELKKAQ
DVLVQEMEVVKQGMSHGELSSEAYNQVWEECYSQVLYLPGQSRYTRANLASKKDRIESLEKRLEINRGH
MTTEAKRAAKMEKKMKILLGGYQSRAMGLMKQLNDLWDQIEQAHLELRTFEELKKHEDSAIPRRLECL
KEDVQRQQEREKELQHRYADLLLEKETLKSKF
>T04734
MGSQPPLGSPLSREEGEAPPPAPASEGRRRSRRVRLRGSCRHRPSFLGCRELAASAPARPAPASSEIMASAA
KEFKMDNFSPKAGTSKLQQTVPADASPDSKCPICLDRFDNVSYLDRCLHKFCFRCVQEWSKNKAECPLCK
QPFDSIFHSVRAEDDFKEYVLRPSYNGSFVTPDRRFRYRTTLTRERNASVYSPSGPVNRRTTTPPDSGVLFE
GLGISTRPRDVEIPQFMRQIAVRRPTTADERSLRKIQEQDIINFRRTLYRAGARVRNIEDGGRYRDISAEFFRR
NPACLHRLVPWLKRELTVLFGAHGSLVNIVQHIIMSNVTRYDLESQAFVSDLRPFLLNRTEHFIHEFISFARS
PFNMAAFDQHANYDCPAPSYEEGSHSDSSVITISPDEAETQELDINVATVSQAPWDDETPGPSYSSSEQVH
VTMSSLLNTSDSSDEELVTGGATSQIQGVQTNDDLNNDSDDSSDNCVIVGFVKPLAERTPELVELSSDSED
LGSYEKMETVKTQEQEQSYSSGDSDVSRCSSPHSVLGKDEQINKGHCDSSTRIKSKKEEKRSTSLSSPRNL
NSSVRGDRVYSPYNHRHRKRGRSRSSDSRSQSRSGHDQKNHRKHHGKKRMKSKRSRSRESSRPRGRRDK
KRSRTRDSSWSRRSQTLSLSSESTSRSRSRSSDHGKRRSRSRNRDRYYLRNNYGSRYKWEYTYYSRNKDR

DGYESSYRRRTLSRAHYSRQSSSPEFRVQSFSERTNARKKNNHSERKYYYYERHRSRSLSSNRSRTASTGT
DRVRNEKPGGKRKYKTRHLEGTNEVAQPSREFASKAKDSHYQKSSSKLDGNYKNESDTFSDSRSSDRETK
HKRRKRKTRSLSVEIVYEGKATDTTKHHKKKKKHKKKHKKHHGDNASRSPVVITIDSDSDKDSEVKED
TECDNSGPQDPLQNEFLAPSLEPFETKDVVTIEAEFGVLDKECDIATLSNNLNNANKTVDNIPPLAASVEQT
LDVREESTFVSDLENQPSNIVSLQTEPSRQLPSPRTSLMSVCLGRDCDMS
>T04742
MGEPAGVAGTMESPFSPGLFHRLDEDWDSALFAELGYFTDTDELQLEAANETYENNFDNLDFDLDLLPW
ESDIWDINNQICTVKDIKAEPQPLSPASSSYSVSSPRSVDSYSSTQHVPEELDLSSSSQMSPLSLYGENSNSLS
SPEPLKEDKPVTGSRNKTENGLTPKKKIQVNSKPSIQPKPLLLPAAPKTQTNSSVPAKTIIIQTVPTLMPLAK
QQPIISLQPAPTKGQTVLLSQPTVVQLQAPGVLPSAQPVLAVAGGVTQLPNHVVNVVPAPSANSPVNGKLS
VTKPVLQSTMRNVGSDIAVLRRQQRMIKNRESACQSRKKKKEYMLGLEARLKAALSENEQLKKENGTLK
RQLDEVVSENQRLKVPSPKRRVVCVMIVLAFIILNYGPMSMLEQDSRRMNPSVGPANQRRHLLGFSAKEA
QDTSDGIIQKNSYRYDHSVSNDKALMVLTEEPLLYIPPPPCQPLINTTESLRLNHELRGWVHRHEVERTKSR
RMTNNQQKTRILQGVVEQGSNSQLMAVQYTETTSSISRNSGSELQVYYASPRSYQDFFEAIRRRGDTFYV
VSFRRDHLLLPATTHNKTTRPKMSIVLPAININENVINGQDYEVMMQIDCQVMDTRILHIKSSSVPPYLRDQ
QRNQTNTFFGSPPAATEATHVVSTIPESLQ
>T00131
MTAKMETTFYDDALNASFLQSESGAYGYSNPKILKQSMTLNLADPVGSLKPHLRAKNSDLLTSPDVGLLK
LASPELERLIIQSSNGHITTTPTPTQFLCPKNVTDEQEGFAEGFVRALAELHSQNTLPSVTSAAQPVSGAGM
VAPAVASVAGAGGGGGYSASLHSEPPVYANLSNFNPGALSSGGGAPSYGAAGLAFPSQPQQQQQPPQPPH
HLPQQIPVQHPRLQALKEEPQTVPEMPGETPPLSPIDMESQERIKAERKRMRNRIAASKCRKRKLERIARLE
EKVKTLKAQNSELASTANMLREQVAQLKQKVMNHVNSGCQLMLTQQLQTF
>T00152
MAWALKLPLADEVIESGLVQDFDASLSGIGQELGAGAYSMSDVLALPIFKQEESSLPPDNENEILPFQYVLC
AATSPAVKLHDETLTYLNQGQSYEIRMLDNRKLGELPELNGKLVKSIFRVVFHDRRLQYTEHQQLEGWRW
NRPGDRILDIDIPMSVGVIDPRANPTQLNTVEFLWDPSKRTSVFIQVHCISTEFTMRKHGGEKGVPFRVQID
TFKENGNGEYTEHLHSASCQIKVFKPKGADRKQKIDREKMEKRTPHEKEKYQPSYETTILTECSPWPEITY
VNNSPSPGFNSSHSSFSLGEGNGSPNHQPEPPPPVTDNLLPTTTPQEAQQWLHRNRFSTFTRLFTNFSGADL
LKLTRDDVIQICGPADGIRLFNALKGRMVRPRLTIYVCQESLQLREQQPQPQPQPQKQEDGDSNGTFFVYH
AIYLEELTAVELTEKIAQLFSISPHQISQIYKQGPTGIHVVISDEMVQNFQEEACFILDTMEAETSDSYHVILK
>T00273
MKSEEDPHEPMAPDIHEERQHRCEDCDQLFESKAELADHQKFPCSTPHSAFSMVEEDLQQNLESESDLREI
HGNQDCKECDRVFPDLQSLEKHMLSHTEEREYKCDQCPKAFNWKSNLIRHQMSHDSGKHYECENCAKV
FTDPSNLQRHIRSQHVGARAHACPECGKTFATSSGLKQHKHIHSSVKPFICEVCHKSYTQFSNLCRHKRMH
ADCRTQIKCKDCGQMFSTTSSLNKHRRFCEGKNHFAAGGFFGQGISLPGTPAMDKTSMVNMSHANPGLA
DYFGTNRHPAGLTFPTAPGFSFSFPGLFPSGLYHRPPLIPASPPVKGLSSTEQSNKCQSPLLTHPQILPATQDIL
KALSKHPPVGDNKPVELLPERSSEERPLEKISDQSESSDLDDVSTPSGSDLETTSGSDLESDLESDKEKCKE
NGKMFKDKVSPLQNLASITNKKEHNNHSVFSASVEEQSAVSGAVNDSIKAIASIAEKYFGSTGLVGLQDKK
VGALPYPSMFPLPFFPAFSQSMYPFPDRDLRSLPLKMEPQSPSEVKKLQKGSSESPFDLTTKRKDEKPLTSG
PSKPSGTPATSQDQPLDLSMGSRGRASGTKLTEPRKNHVFGEKKGSNMDTRPSSDGSLQHARPTPFFMDPI
YRVEKRKLTDPLEALKEKYLRPSPGFLFHPQMSAIENMAEKLESFSALKPEASELLQSVPSMFSFRAPPNTL
PENLLRKGKERYTCRYCGKIFPRSANLTRHLRTHTGEQPYRCKYCDRSFSISSNLQRHVRNIHNKEKPFKC
HLCDRCFGQQTNLDRHLKKHENGNMSGTATSSPHSELESAGAILDDKEDAYFTEIRNFIGNSNHGSQSPRN
MEERMNGSHFKDKKALATSQNSDLLDDEEVEDEVLLDEEDEDNDIPGKPRKELGVTRLDEEIPEDDYEEA

GALEMSCKASPVRYKEEDYKSGLSALDHIRHFTDSLKMREMEENQYTDAELSSISSSHVPEELKQTLHRK
SKSQAYAMMLSLSDKDSLHPTSHSSSNVWHSMARAAAESSAIQSISHV
>T00422
MPITRMRMRPWLEMQINSNQIPGLIWINKEEMIFQIPWKHAAKHGWDINKDACLFRSWAIHTGRYKAGE
KEPDPKTWKANFRCAMNSLPDIEEVKDQSRNKGSSAVRVYRMLPPLTRNQRKERKSKSSRDTKSKTKRK
LCGDVSPDTFSDGLSSSTLPDDHSSYTTQGYLGQDLDMERDITPALSPCVVSSSLSEWHMQMDIIPDSTTD
LYNLQVSPMPSTSEAATDEDEEGKIAEDLMKLFEQSEWQPTHIDGKGYLLNEPGTQLSSVYGDFSCKEEPE
IDSPRGDIGIGIQHVFTEMKNMDSIMWMDSLLGNSVRLPPSIQAIPCAP
>T00437
METPFYGEEALSGLAAGASSVAGATGAPGGGGFAPPGRAFPGAPPTSSMLKKDALTLSLAEQGAAGLKPG
SATAPSALRPDGAPDGLLASPDLGLLKLASPELERLIIQSNGLVTTTPTSTQFLYPKVAASEEQEFAEGFVKA
LEDLHKQSQLGAATAATSGAPAPPAPADLAATPGATETPVYANLSSFAGGAGPPGGAATVAFAAEPVPFPPP
PGALGPPPPPHPPRLAALKDEPQTVPDVPSFGDSPPLSPIDMDTQERIKAERKRLRNRIAASKCRKRKLERIS
RLEEKVKTLKSQNTELASTASLLREQVAQLKQKVLSHVNSGCQLLPQHQVPAY
>T00526
MELLSPPLRDIDLTGPDGSLCSFETADDFYDDPCFDSPDLRFFEDLDPRLVHVGALLKPEEHAHFSTAVHPG
PGAREDEHVRAPSGHHQAGRCLLWACKACKRKTTNADRRKAATMRERRRLSKVNEAFETLKRCTSSNP
NQRLPKVEILRNAIRYIEGLQALLRDQDAAPPGAAAFYAPGPLPPGRGSEHYSGDSDASSPRSNCSDGMM
DYSGPPSGPRRQNGYDTAYYSEAVRESRPGKSAAVSSLDCLSSIVERISTDSPAAPALLLADAPPESPPGPPE
GASLSDTEQGTQTPSPDAAPQCPAGSNPNAIYQVL
>T00528
MELYETSPYFYQEPHFYDGENYLPVHLQGFEPPGYERTELSLSPEARGPLEEKGLGTPEHCPGQCLPWACK
VCKRKSVSVDRRRAATLREKRRLKKVNEAFEALKRSTLLNPNQRLPKVEILRSAIQYIERLQALLSSLNQE
ERDLRYRGGGGPQPMVPSECNSHSASCSPEWGNALEFGPNPGDHLLAADPTDAHNLHSLTSIVDSITVED
MSVAFPDETMPN
>T00595
MDDLFPLIFPSEPAQASGPYVEIIEQPKQRGMRFRYKCEGRSAGSIPGERSTDTTKTHPTIKINGYTGPGTVR
ISLVTKDPPHRPHPHELVGKDCRDGYYEADLCPDRSIHSFQNLGIQCVKKRDLEQAISQRIQTNNNPFHVPI
EEQRGDYDLNAVRLCFQVTVRDPAGRPLLLTPVLSHPIFDNRAPNTAELKICRVNRNSGSCLGGDEIFLLCD
KVQKEDIEVYFTGPGWEARGSFSQADVHRQVAIVFRTPPYADPSLQAPVRVSMQLRRPSDRELSEPMEFQ
YLPDTDDRHRIEEKRKRTYETFKSIMKKSPFNGPTEPRPPTRRIAVPTRNSTSVPKPAPQPYTFPASLSTINFD
EFSPMLLPSGQISNQALALAPSSAPVLAQTMVPSSAMVPLAQPPAPAPVLTPGPPQSLSAPVPKSTQAGEGT
LSEALLHLQFDADEDLGALLGNSTDPGVFTDLASVDNSEFQQLLNQGVSMSHSTAEPMLMEYPEAITRLV
TGSQRPPDPAPTPLGTSGLPNGLSGDEDFSSIADMDFSALLSQISS
>T00677
MEQTYGEVNQLGGVFVNGRPLPNAIRLRIVELAQLGIRPCDISRQLRVSHGCVSKILARYNETGSILPGAIG
GSKPRVTTPNVVKHIRDYKQGDPGIFAWEIRDRLLADGVCDKYNVPSVSSISRILRNKIGSLAQPGPYEASK
QPPPQPALPYNHIYQYPYPSPVSPTGTKMGTHPGVPGSAGHVSIPRSWPSAHSVSNILGIRTFMEQTGALTG
SEGAAYSPKMEDWAGVNRAAFPTSPAVNGLEKPALEADIKYTQLASSLSAVGGFLPACAYPASNQQGVYS
APAAGYLSPGPPWPPAQAPPLTPHGAGVAVHGGELAAAMTFKHREGTDRKPPSPGGKATDALGSLHGLPIP
ASTS
>T00680
MTTLAGAVPRMMRPGPGQNYPRSGFPLEVSTPLGQGRVNQLGGVFINGRPLPNHIRHKIVEMAHHGIRPC
VISRQLRVSHGCVSKILCRYQETGSIRPGAIGGSKPKQVTTPDVEKKIEEYKRENPGMFSWEIRDKLLKDAV

CDRNTVPSVSSISRILRSKFGKGEEEEADLERKEAEESEKKAKHSIDGILSERASAPQSDEGSDIDSEPDLPL
KRKQRRSRTTFTAEQLEELERAFERTHYPDIYTREELAQRAKLTEARVQVWFSNRRARWRKQAGANQLM
AFNHLIPGGFPPTAMPTLPTYQLSEHSYQPTSIPQAVSDPSSTVHRPQPLPPSTVHQSTIPSNADSSSAYCLPS
TRHGFSSYTDSFVPPSGPSNPMNPTIGNGLSPQVMGLLTNHGGVPHQPQTDYALSPLTGGLEPTTTVSASCS
QRLEHMKNVDSLPTSQPYCPPTYSTAGYSMDPVTGYQYGQYGQSKPWTF
>T00684
MTKSSNHNCLLRPENKPGLWGPGAQAASLRPSPATLVVSSPGHAEHPPAAPAQTPGPQVSASARGPGPVA
GGSGRMERRMKGGYLDQRVPYTFCSKSPGNGSLGEALMVPQGKLMDPGSLPPSDSEDLFQDLSHFQETW
LAEAQVPDSDEQFVPDFHSENLAFHSPTTRIKKEPQSPRTDPALSCSRKPPLPYHHGEQCLYSRQIAIKSPAP
GAPGQSPLQPFSRAEQQQSLLRASSSSQSHPGHGYLGEHSSVFQQPVDMCHSFTSPQGGGREPLPAPYQHQ
LSEPCPPYPQQNFKQEYHDPLYEQAGQPASSQGGVSGHRYPGAGVVIKQERTDFAYDSDVPGCASMYLHP
EGFSGPSPGDGVMGYGYEKSLRPFPDDVCIVPKKFEGDIKQEGIGAFREGPPYQRRGALQLWQFLVALLD
DPTNAHFIAWTGRGMEFKLIEPEEVARLWGIQKNRPAMNYDKLSRSLRYYYEKGIMQKVAGERYVYKFV
CEPEALFSLAFPDNQRPALKAEFDRPVSEEDTVPLSHLDESPAYLPELTGPAPPFGHRGGYSY
>T00765
MLPSQAGAAAALGRGSALGGNLNRTPTGRPGGGGGTRGANGGRVPGNGAGLGQSRLEREAAAAAAPTA
GALYSGSEGDSESGEEEELGAERRGLKRSLSEMELGVVVGGPEAAAAAAGGYGPVSGAVSGAKPGKKTR
GRVKIKMEFIDNKLRRYTTFSKRKTGIMKKAYELSTLTGTQVLLLVASETGHVYTFATRKLQPMITSETGK
ALIQTCLNSPDSPPRSDPTTDQRMSATGFEEPDLTYQVSESDSSGETKDTLKPAFTVTNLPGTTSTIQTAPST
STTMQVSSGPSFPITNYLAPVSASVSPSAVSSANGTVLKSTGSGPVSSGGLMQLPTSFTLMPGGAVAQQVP
VQAIHVHQAPQQASPSRDSSTDLTQTSSSGTVTLPATIMTSSVPTTVGGHMMYPSPHAVMYAPTSGLADGS
LTVLNAFSQAPSTMQVSHSQVQEPGGVPQVFLTAPSGTVQIPVSAVQLHQMAVIGQQAGSSSNLTELQVVN
LDATHSTKSE
>T00930
MPQLSGGGGGGDPELCATDEMIPFKDEGDPQKEKIFAEISHPEEEGDLADIKSSLVNESEIIPASNGHEVVRQ
APSSQEPYHDKAREHPDEGKHPDGGLYNKGPSYSSYSGYIMMPNMNSDPYMSNGSLSPPIPRTSNKVPVV
QPSHAVHPLTPLITYSDEHFSPGSHPSHIPSDVNSKQGMSRHPPAPEIPTFYPLSPGGVGQITPPIGWQGQPV
YPITGGFRQPYPSSLSGDTSMSRFSHHMIPGPPGPHTTGIPHPAIVTPQVKQEHPHTDSDLMHVKPQHEQRK
EQEPKRPHIKKPLNAFMLYMKEMRANVVAECTLKESAAINQILGRRWHALSREEQAKYYELARKERQLH
MQLYPGWSARDNYGKKKKRKREKLQESTSGTGPRMTAAYI
>T01112
MFGIQESIQRSGSSMKEEPLGSGMNAVRTWMQGAGVLDANTAAQSGVGLARAHFEKQPPSNLRKSNFFH
FVLALYDRQGQPVEIERTAFVGFVEKEKEANSEKTNNGIHYRLQLLYSNGIRTEQDFYVRLIDSMTKQAIV
YEGQDKNPEMCRVLLTHEIMCSRCCDKKSCGNRNETPSDPVIIDRFFLKFFLKCNQNCLKNAGNPRDMRR
FQVVVSTTVNVDGHVLAVSDNMFVHNNSKHGRRARRLDPSEGTPSYLEHATPCIKAISPSEGWTTGGATV
IIIGDNFFDGLQVIFGTMLVWSELITPHAIRVQTPPRHIPGVVEVTLSYKSKQFCKGTPGRFIYTALNEPTIDY
GFQRLQKVIPRHPGDPERLPKEVILKRAADLVEALYGMPHNNQEIILKRAADIAEALYSVPRNHNQLPALA
NTSVHAGMMGVNSFSGQLAVNVSEASQATNQGFTRNSSSVSPHGYVPSTTPQQTNYNSVTTSMNGYGSA
AMSNLGGSPTFLNGSAANSPYAIVPSSPTMASSTSLPSNCSSSSGIFSFSPANMVSAVKQKSAFAPVVRPQTS
PPPTCTSTNGNSLQAISGMIVPPM
>T01311
MTMESGADNQQSGDAAVTEAENQQMTVQAQPQIATLAQVSMPAAHATSSAPTVTLVQLPNGQTVQVHG
VIQAAQPSVIQSPQVQTVQISTIAESEDSQESVDSVTDSQKRREILSRRPSYRKILNDLSSDAPGVPRIEEEKS
EEETSAPAITTVTVPTPIYQTSSGQYIAITQGGAIQLANNGTDGVQGLQTLTMTNAAATQPGTTILQYAQTT

DGQQILVPSNQVVVQAASGDVQTYQIRTAPTSTIAPGVVMASSPALPTQPAEEAARKREVRLMKNREAAR
ECRRKKKEYVKCLENRVAVLENQNKTLIEELKALKDLYCHKSD
>T01445
MPSCTASTMPGMICKNPDLEFDSLQPCLHPDEDDFHFGGPDSTPPGEDIWKKFELLPTPPLSPSRAFPEHSP
EPSNWATEMLLPEADLWGNPAEEDAFGLGGLGGLTPNPVILQDCMWSGFSAREKLERAVNEKLQHGHGP
PGVSSACSAPGVGASSPGGRALGGSSSASHTGATLPTDLSHPAAECVDPAVVFPFPVNKRESASVPAAPTSA
PATSAAVTSVSVPATAPVAAPARAGGRPASSGGTKALSTSGEDTLSDSDDEDDEEEDEEEEIDVVTVEKRRS
SSNNKAVTTFTITVRPKTSALGLGRAQLGELILKRCVPIHQQHNYAAPSPYVESEDAPPQKKIKSEASPRPL
KSVVPAKAKSLSPRNSDSEDSERRRNHNILERQRRNDLRSSFLTLRDHVPELVKNEKAAKVVILKKATEYV
HALQANEHQLLLEKEKLQARQQQLLKKIEHARTC
>T01543
MAVAPAGGQHAPALEALLGAGALRLLDSSQIVIISTAPDVGAPQLPAAPPTGPRDSDVLLFATPQAPRPAPS
APRPALGRPPVKRRLDLETDHQYLAGSSGPFRGRGRHPGKGVKSPGEKSRYETSLNLTTKRFLELLSRSAD
GVVDLNWAAEVLKVQKRRIYDITNVLEGIQLIAKKSKNHIQWLGSHTMVGIGKRLEGLTQDLQQLQESEQ
QLDHLMHICTTQLQLLSEDSDTQRLAYVTCQDLRSIADPAEQMVIVIKAPPETQLQAVDSSETFQISLKSKQ
GPIDVFLCPEESADGISPGKTSCQETSSGEDRTADSGPAGPPPSPPSTSPALDPSQSLLGLEQEAVLPRMGHLR
VPMEEDQLSPLVAADSLLEHVKEDFSGLLPGEFISLSPPHEALDYHFGLEEGEGIRDLFDCDFGDLTPLDF
>T01554
MLEMLEYSHYQVQTHLENPTKYHIQQAQRHQVKQYLSTTLANKHASQVLSSPCPNQPGDHAMPPVPGSS
APNSPMAMLTLNSNCEKEAFYKFEEQSRAESECPGMNTHSRASCMQMDDVIDDIISLESSYNEEILGLMDP
ALQMANTLPVSGNLIDLYSNQGLPPPGLTISNSCPANLPNIKRELTACIFPTESEARALAKERQKKDNHNLIE
RRRRFNINDRIKELGTLIPKSNDPDMRWNKGTILKASVDYIRKLQREQQRAKDLENRQKKLEHANRHLLL
RVQELEMQARAHGLSLIPSTGLCSPDLVNRIIKQEPVLENCSQELVQHQADLTCTTTLDLTDGTITFTNNLG
TMPESSPAYSIPRKMGSNLEDILMDDALSPVGVTDPLLSSVSPGASKTSSRRSSMSAEETEHAC
>T01710
MATTGALGNYYVDSFLLGADAADELGAGRYAPGTLGQPPRQAAALAEHPDFSPCSFQSKAAVFGASWNP
VHAAGANAVPAAVYHHHHHPYVHPQAPVAAAAPDGRYMRSWLEPTPGALSFAGLPSSRPYGIKPEPLSAR
RGDCPTLDTHTLSLTDYACGSPPVDREKQPSEGAFSENNAENESGGDKPPIDPNNPAANWLHARSTRKKR
CPYTKHQTLELEKEFLFNMYLTRDRRYEVARLLNLTERQVKIWFQNRRMKMKKINKDRAKDE
>T01788
RAGAPSALSPNYDAGLHGLQSKMEDRLDEAIHVLRSHAVGTASDLHGLLPGHGALTTSFTGPMSLGGRH
AGLVGGSHPEEGLTSGASLLHNHASLPSQPSSLPDLSQRPPDSYSGLGRAGTTAGASEIKREEKEDEEIASV
ADAEEDKKDLKVPRTRTSSTDEVLSLEEKDLRDRERRMANNARERVRVRDINEAFRELGRMCQLHLKSD
KAQTKLLILQQAVQVILGLEQQVRERNLNPKAACLKRREEEKVSGVVGDPQLALSAAHPGLGEAHNPAG
HL
>T01806
MTAMEESQSDISLELPLSQETFSGLWKLLPPEDILPSPHCMDDLLLPQDVEEFFEGPSEALRVSGAPAAQDP
VTETPGPVAPAPATPWPLSSFVPSQKTYQGNYGFHLGFLQSGTAKSVMCTYSPPLNKLFCQLAKTCPVQL
WVSATPPAGSRVRAMAIYKKSQHMTEVVRRCPHHERCSDGDGLAPPQHLIRVEGNLYPEYLEDRQTFRHS
VVVPYEPPEAGSEYTTIHYKYMCNSSCMGGMNRRPILTIITLEDSSGNLLGRDSFEVRVCACPGRDRRTEE
ENFRKKEVLCPELPPGSAKRALPTCTSASPPQKKKPLDGEYFTLKIRGRKRFEMFRELNEALELKDAHATE
ESGDSRAHSSYLKTKKGQSTSRHKKTMVKKVGPDSD
>T01836
MMETELKPPGPQQASGGGGGGGKPTAAATGGNQKNSPDRVKRPMNAFMVWSRGQRRKMAQENPKMH

NSEISKRLGAEWKLLSETEKRPFIDEAKRLRALHMKEHPDYKYRPRRKTKTLMKKDKYTLPGGLLAPGG
NSMASGVGVGAGLGGGLNQRMDSYAHMNGWSNGSYSMMQEQLGYPQHPGLNAHGAAQMQPMHRYV
VSALQYNSMTSSQTYMNGSPTYSMSISQQGTPGMALGSMGSVVKSEASSSPPVVTSSSHSRAPCQAGDLR
DMISMYLPGAEVPEPAAPSRLHMAQLILCGPVPGTAKYGTLPLSHM
>T01873
MATAASNHYSLLTSSASIVHAEPPGGMQQGAGGYREAQSLVQGDYGALQSNGHPLSHAHQWITALSHGG
GGGGGGGGGGGGGGGGGGGDGSPWSTSPLGQPDIKPSVVVQQGGRGDELHGPGALQQQHQQQQQQQ
QQQQQQQQQQQQQQQRPPHLVHHAANHHPGPGAWRSAAAAAHLPPSMGASNGGLLYSQPSFTVNGML
GAGGQPAGLHHHGLRDAHDEPHHADHHPHPHSHPHQQPPPPPPPQGPPGHPGAHHDPHSDEDTPTSDDL
EQFAKQFKQRRIKLGFTQADVGLALGTLYGNVFSQTTICRFEALQLSFKNMCKLKPLLNKWLEEADSSSG
SPTSIDKIAAQGRKRKKRTSIEVSVKGALESHFLKCPKPSAQEITSLADSLQLEKEVVRVWFCNRRQKEKR
MTPPGGTLPGAEDVYGGSRDTPPHHGVQTPVQ
>T01888
MPGISSQILTNAQGQVIGALPWVVNSASVATPAPAQSLQVQAVTPQLLLNAQGQVIATLASSPLPQPVAVRK
PNTPESPAKSEVQPIQPTQAVPQPAVILTSPTPALKPSAATPIPITCSETPTVSQLVSKPHTPSLDEDGINLEEIR
EFAKNFKIRRLSLGLTQTQVGQALTATEGPAYSQSAICRFEKLDITPKSAQKLKPVLEKWLMEAELRNQEG
QQNLMEFVGGEPSKKRKRRTSFTPQAIEALNAYFEKNPLPTGQEITEIAKELNYDREVVRVWFCNRRQTL
KNTSKLNVFQIP
>T02016
MEEQQPEPKSQRDSGLGAVAAAAPSGLSLSLSPGASGSSGSDGDSVPVSPQPAPPSPPAAPCLPPLAHHPHL
PPHPPPPPPPPPPPPQHLAAPAHQPQPAAQLHRTTNFFIDNILRPDFGCKKEQPLPQLLVASAAAGGGAAAG
GGSRVERDRGQTGAGRDPVHSLGTRASGAASLLCAPDANCGPPDGSQPATAVGAGASKAGNPAAAAAAA
AAAAAAAVAAAAAAAAASKPSDSGGGSGGNAGSPGAQGAKFPEHNPAILLMGSANGGPVVKTDSQQPLVW
PAWVYCTRYSDRPSSGPRTRKLKKKKNEKEDKRPRTAFTAEQLQRLKAEFQANRYITEQRRQTLAQELSL
NESQIKIWFQNKRAKIKKATGIKNGLALHLMAQGLYNHSTTTVQDKDESE
>T02532
MYQSLAMAANHGPPPGAYEAGGPGAFMHSAGAASSPVYVPTPRVPSSVLGLSYLQGGGSAAAAGTTSG
GSSGAGPSGAGPGTQQGSPGWSQAGAEGAAYTPPPVSPRFSFPGTTGSLAAAAAAAAAAREAAAYGSGGG
AAGAGLAGREQYGRPGFAGSYSSPYPAYMADVGASWAAAAAAASAGPFDSPVLHSLPGRANPGRHPNLD
MFDDFSEGRECVNCGAMSTPLWRRDGTGHYLCNACGLYHKMNGINRPLIKPQRRLSASRRVGLSCANCQ
TTTTTLWRRNAEGEPVCNACGLYMKLHGVPRPLAMRKEGIQTRKRKPKNLNKSKTPAGPAGETLPPSSGA
SSGNSSNATSSSSSSEEMRPIKTEPGLSSHYGHSSSMSQTSVCVRPRALHPSSAVCSKLSPQGYASPVTQTSQ
ASSKQDSWNSLVLADSHGDIITA
>T03389
MAQRYDDLPHYGGMDGVGIPSTMYGDPHAARSMQPVHHLNHGPPLHSHQYPHTAHTNAMAPSMGSSV
NDALKRDKDAIYGHPLFPLLALIFEKCELATCTPREPGVAGGDVCSSESFNEDIAVFAKQIRAEKPLFSSNPE
LDNLMIQAIQVLRFHLLELEKVHELCDNFCHRYISCLKGKMPIDLVIDDREGGSKSDSEDVTRSANLTDQP
SWNRDHDDTASTRSGGTPGPSSGGHTSHSGDNSSEQGDGLDNSVASPSTGDDDDPDKDKKRHKKRGIFP
KVATNIMRAWLFQHLTHPYPSEEQKKQLAQDTGLTILQVNNWFINARRRIVQPMIDQSNRAVSQGTPYNP
DGQPMGGFVMDGQQHMGIRAPGLQSMPGEYVARGGPMGVSMGQPSYTQAQMPPHPAQLRHGPPMHTY
IPGHPHHPAVMMHGGQPHPGMPMSASSPSVLNTGDPTMSAQVMDIHAQ
>T04347
MADTDEGFGLARTPLEPDSKDRSCDSKPESALGAPSKSPSSPQAAFTQQGMEGIKVFLHERELWLKFHEV
GTEMIITKAGRRMFPSYKVKVTGLNPKTKYILLMDIVPADDHRYKFADNKWSVTGKAEPAMPGRLYVHP

DSPATGAHWMRQLVSFQKLKLTNNHLDPFGHIILNSMHKYQPRLHIVKADENNGFGSKNTAFCTHVFPET
AFIAVTSYQNHKITQLKIENNPFAKGFRGSDDLELHRMSRMQSKEYPVVPRSTVRHKVTSNHSPFSSETRA
LSTSSNLGSQYQCENGVSGPSQDLLPPPNPYPLAQEHSQIYHCTKRKDEECSSTEHPYKKPYMETSPSEED
TFYRSGYPQQQGLSTSYRTESAQRQACMYASSAPPSEPVPSLEDISCNTWPSMPSYSSCTVTTVQPMDRLP
YQHFSAHFTSGPLVPRLAGMANHGSPQLGEGMFQHQTSVAHQPVVRQCGPQTGLQSPGGLQPPEFLYTH
GVPRTLSPHQYHSVHGVGMVPEWSENS
>T04446
MKSPVSLLLDERPAHKDGPTEASQCSCAHVTVGIPISFPLAGWSQSFPHMSLSFLGALGFPLCKPPSELPLV
LHSLRASAGGEMSLSRWVSKASPRGSSLKHTAEAPQTCTHSSKIAYTLVPNGAVRPLLAWSYSIPPSAPGIE
NLGKQPADQGHQNAKVDFHTQGSGQRASPRPQGALEGAAGFALSQVGDLAFPRFEIPAQRFALPAHYLER
SPAWWYPYTLTPAGGHLPRPEASEKALLRDSSPASGTDRDSPEPLLKADPDHKELDSKSPDEIILEESDSEE
GKKEGEAVPGAAGTTVGATTATPGSEDWKAGAESPEKKPACRKKKTRTVFSRSQVFQLESTFDMKRYLSS
SERAGLAASLHLTETQVKIWFQNRRNKWKRQLAAELEAANLSHAAAQRIVRVPILYHENSAAEGAAAAA
GAPVPVSQPLLTFPHPVYYSHPVVSSVPLLRPV
>T04671
MTMLLDGGPQFPGLGVGSFGAPRHHEMPNREPAGMGLNPFGDSTHAAAAAAAAAAAFKLSPATAHDLSSG
QSSAFTPQGSGYANALGHHHHHHHHHHASQVPTYARRASAAFNSTRDFLFRQRGSGLSEAASGGGQHGL
FAGSASSLHAPAGIPEPPSYLLFPGLHEQGAGHPSPTGHVDNNQVHLGLRGELFGRADPYRPVASPRTDPY
AASAQFPNYSPMNMNMGVNVAAHHGPGAFFRYMRQPIKQELSCKWIEEAQLSRPKKSCDRTFSTMHELV
THVTMEHVGGPEQNNHVCYWEECPREGKSFKAKYKLVNHIRVHTGEKPFPCPFPGCGKIFARSENLKIHK
RTHTGEKPFKCEFEGCDRRFANSSDRKKHMHVHTSDKPYICKVCDKSYTHPSSLRKHMKVHESQGSDSSP
AASSGYESSTPPAIASANSKDTTKTPSAVQTSTSHNPGLPPNFNEWYV
>T04811
MMQESGSETKSNGSAIQNGSSGGNHLLECGALRDTRSNGEAPAVDLGAADLAHVQQQQQALQVARQL
LLQQQQQQQQQQQQQQQQQQQQQQQQQQQQQQQQQQQQVSGLKSPKRNDKQPALQVPVSVAMMTP
QVITPQQMQQILQQQVLSPQQLQVLLQQQQALMLQQQLQEFYKKQQEQLQLQLLQQQHAGKQPKEQQV
ATQQLAFQQQLLQMQQLQQQHLLSLQRQGLLTIQPGQPALPLQPLAQGMIPTELQQLWKEVTSAHTAEET
TSSNHSSLDLTSTCVSSSAPSKSSLIMNPHASTNGQLSVHTPKRESLSHEEHPHSHPLYGHGVCKWPGCEAV
CDDFPAFLKHLNSEHALDDRSTAQCRVQMQVVQQLELQLAKDKERLQAMMTHLHVKSTEPKAAPQPLN
LVSSVTLSKSASEASPQSLPHTPTTPTAPLTPVTQGPSVITTTSMHTVGPIRRRYSDKYNVPISSADIAQNQEF
YKNAEVRPPFTYASLIRQAILESPEKQLTLNEIYNWFTRMFAYFRRNAATWKNAVRHNLSLHKCFVRVENV
KGAVWTVDEVEFQKRRPQKISGNPSLIKNMQSSHAYCTPLNAALQASMAENSIPLYTTASMGNPTLGSLA
SAIREELNGAMEHTNSNESDSSPGRSPMQAVHPIHVKEEPLDPEEAEGPLSLVTTANHSPDFDHDRDYEDE
PVNEDME
>T08231
MESGTVLLESKSSPLNLLHEMHELRLLGHLCDVTVSIENQGVHEDFMAHKAVLAATSKFFKEVFLNEKSA
DGTRTNVYLNEVQAVDFASFLEFVYTAKVRVEEDRVQQMLEVAEKLKCLDLSETCLQLKKQMLESVLLE
LQNFSESQEVEASSGPQVSVTPSSKASVPGEDAHSNGLVDSSDYPIERLGNGLSPETPSKKCKEKLDKKKD
VAKPPFPKIRRASGRLAGKKVFVEIPKKKYTRRLREQQKSAEEAAENDKCPQDQSPDNERMETEPAAKSE
ACPASVELEESLQKVEGEKEEEEGKDGEEKKKSNFQCTVCDKAFLYEKSFLKHIKYHHGVATEVVYRCDT
CGQTFANRCNLKSHQRHVHSSERHFPCEMCAKKFKRKKDVKRHVLQVHEGGGERHRCGQCGKGLSSKT
ALRLHERTHTGDKPYGCTKCDAKFSQPSALKTHLRVHTGERPFVCDECGARFTQNHMLIYHKRCHTGER
PFMCETCGKSFASKEYLKHHNRIHTGSKPFKCEVCLRTFAQRNSLYQHIKVHTGERPYCCDQCGKQFTQV
NALQRHHRIHTGEKPYMCNACGRTFTDKSTLRRHTSIHDKNTPWKSFLVIVDGSPKNDEGHKTEQPDDEY

ASPKLSDRLLSFGENSHFNNLLEVQGNVPAVQENSSTGAACKAVVSQDALLTTSISALGELTPQAVSMPAH
LPSLTNME
>T00109
MSAALFSLDSPARGAPWPTEPAAFYEPGRVGKPGRGPEPGDLGEPGSTTPAMYDDESAIDFSAYIDSMAAV
PTLELCHDEIFADLFNSNHKAAGAGSLELLQGGPTRPPGVGSIARGPLKREPDWGDGDAPGSLLPAQVAVC
AQTVVSLAAAAQPTPPTSPEPPRGSPGPSLAPGPVREKGAGKRGPDRGSPEYRQRRERNNIAVRKSRDKA
KRRNQEMQQKLVELSAENEKLHQRVEQLTRDLASLRQFFKELPSPPFLPPTGTDCR
>T00124
MMFSGFNADYEASSSRCSSASPAGDSLSYYHSPADSFSSMGSPVNTQDFCADLSVSSANFIPTVTAISTSPD
LQWLVQPTLVSSVAPSQTRAPHPYGLPTPSTGAYARAGVVKTMSGGRAQSIGRRGKVEQLSPEEEEKRRIR
RERNKMAAAKCRNRRRELTDTLQAETDQLEDEKSALQTEIANLLKEKEKLEFILAAHRPACKIPNDLGFPE
EMSVTSLDLTGGLPEATTPESEEAFTLPLLNDPEPKPSLEPVKNISNMELKAEPFDDFLFPASSRPSGSETARS
VPDVDLSGSFYAADWEPLHSSSLGMGPMVTELEPLCTPVVTCTPSCTTYTSSFVFTYPEADSFPSCAAAHR
KGSSSNEPSSDSLSSPTLLAL
>T00132
MTAKMETTFYDDALNASFLQSESGAYGYSNPKILKQSMTLNLADPVGNLKPHLRAKNSDLLTSPDVGLL
KLASPELERLIIQSSNGHITTTPTPTQFLCPKNVTDEQEGFAEGFVRALAELHSQNTLPSVTSAAQPVSGAG
MVAPAVASVAGAGGGGGYSASLHSEPPVYANLSNFNPGALSSGGGAPSYGATGLAFPSQPQQQQPPQPP
HHLPQQIPVQHPRLQALKEEPQTVPEMPGETPPLSPIDMESQERIKAERKRMRNRIAASKCRKRKLERIAR
LEEKVKTLKAQNSELASTANMLREQVAQLKQKVMNHVNSGCQLMLTQQLQTF
>T00164
MTMDSGADNQQSGDAAVTEAESQQMTVQAQPQIATLAQVSMPAAHATSSAPTVTLVQLPNGQTVQVHG
VIQAAQPSVIQSPQVQTVQSSCKDLKRLFSGTQISTIAESEDSQESVDSVTDSQKRREILSRRPSYRKILNDL
SSDAPGVPRIEEEKSEEETSAPAITTVTVPTPIYQTSSGQYIAITQGGAIQLANNGTDGVQGLQTLTMTNAA
ATQPGTTILQYAQTTDGQQILVPSNQVVVQAASGDVQTYQIRTAPTSTIAPGVVMASSPALPTQPAEEAAR
KREVRLMKNREAARECRRKKKEYVKCLENRVAVLENQNKTLIEELKALKDLYCHKSD
>T00183
MARPLSDRTPGPLLLGGPAGAPPGGGALLGLRSLLQGNSKPKEPASCLLKEKERKATLPSAPVPGPVLETA
GPADAPTGAVSGGGSPRGRSGPVAGPSLFAPLLWERTLPFGDVEYVDLDAFLLEHGLPPSPPPPGGLSPAPS
PARTPAPSPGPGSCSSSSPRSSPGHAPARATLGAAGGHRAGLTSRDTPSPVDPDTVEVLMTFEPDPADLALS
SIPGHETFDPRRHRFSEEELKPQPIMKKARKVQVPEEQKDEKYWSRRYKNNEAAKRSRDARRLKENQISV
RAAFLEKENALLRQEVVAVRQELSHYRAVLSRYQAQHGTL
>T00258
MTMTLHTKASGMALLHQIQGNELEPLNRPQLKMPMERALGEVYVDNSKPAVFNYPEGAAYEFNAAAAA
AAAGASAPVYGQSSITYGPGSEAAAFGANSLGAFPQLNSVSPSPLMLLHPPPHVSPFLHPHGHQVPYYLEN
EPSAYAVRDTGPPAFYRSNSDNRRQNGRERLSSSSEKGNMIMESAKETRYCAVCNDYASGYHYGVWSCEG
CKAFFKRSIQGHNDYMCPATNQCTIDKNRRKSCQACRLRKCYEVGMMKGGIRKDRRGGRMLKHKRQRD
DLEGRNEMGTSGDMRAANLWPSPLVIKHTKKNSPALSLTADQMVSALLDAEPPLIYSEYDPSRPFSEASM
MGLLTNLADRELVHMINWAKRVPGFGDLNLHDQVHLLECAWLEILMIGLVWRSMEHPGKLLFAPNLLLD
RNQGKCVEGMVEIFDMLLATSSRFRMMNLQGEEFVCLKSIILLNSGVYTFLSSTLKSLEEKDHIHRVLDKI
NDTLIHLMAKAGLTLQQQHRRLAQLLLILSHIRHMSNKGMEHLYNMKCKNVVPLYDLLLEMLDAHRLH
APASRMGVPPEEPSQSQLTTTSSTSAHSLQTYYIPPEAEGFPNTI
>T00424
MPITRMRMRPWLEMQINSNQIPGLSWINKEEMIFQIPWKHAALHGWDINKDACLFRSWAIHTGRYKAGE

KEPDPKTWKANFRCAMNSLPDIEEVKDQSRNKGSSAVRVYRMLPPLTKNQRKERKSKSSRDTKSKTKRK
LCGDSSPDTLSDGLSSSTLPDDHSSYTAQGYLGQDLDMDRDITPALSPCVVSSSLSEWHMQMDIMPDSTT
DLYNLQVSPMPSTSEAATDEDEEGKLPEDIMKLFEQSEWQPTHVDGKGYLLNEPGAQLSTVYGDFSCKEE
PEIDSPGGDIEIGIQRVFTEMKNMDPVMWMDTLLGNSTRPPSIQAIPCAP
>T00691
MSCQPFTSADTFIPLNSDASAALPLRMHHSAAEGLPASNHATNVMSTVPSILSLIQTPKCLHTYFSMTTMG
NTATGLHYSVPSCHYGNQPSTYGVMAGTLTPCLYKFPDHTLSHGFPPLHQPLLAEDPTASEFKQELRRKSK
LVEEPIDMDSPEIRELEQFANEFKVRRIKLGYTQTNVGEALAAVHGSEFSQTTICRFENLQLSFKNACKLKA
ILSKWLEEAEQVGALYNEKVGANERKRKRRTTISIAAKDALERHFGEHSKPSSQEIMRMAEELNLEKEVV
RVWFCNRRQREKRVKTSLNQSLFSISKEHLECR
>T00754
MSDQDHSMDEVTAVKIEKGVGGNNGGSGNGGGAAFSQTRSSSTGSSSSSGGGGGQESQPSPLALLAATCS
RIESPNENENSNNSQGPSQSGGTGELDLTATQLSQGANGWQIISSSSGATPTSKEQSGNSTNGSNGSESSKN
RTVSGGQYVVAATPNLQNQQVLTGLPGVMPNIQYQVIPQFQTVDGQQLQFAATGAQVQQDGSGQIQIIPG
ANQQIITNRGSGGNIIAAMPNLLQQAVPLQGLANNVLSGQTQYVTNVPVALNGNITLLPVNSVSAATLTPS
SQAGTISSSGSQESGSQPVTSGTAISSASLVSSQASSSSFFTNANSYSTTTTTSNMGIMNFTSSGSSGTSSQGQ
TSQRVGGLQGSDSLNIQQNQTSGGSLQGSQQKEGEQSQQTQQQQILIQPQLVQGGQALQALQAAPLSGQT
FTTQAISQETLQNLQLQAVQNSGPIIIRTPTVGPNGQVSWQTLQLQNLQVQNPQAQTITLAPMQGVSLGQT
SSSNTTLTPIASAASIPAGTVTVNAAQLSSMPGLQTINLSALGTSGIQVHQLPGLPLAIANTPGDHGAQLGL
HGPGGDGIHDETAGGEEGENSPDPQPQAGRRTRREACTCPYCKDSEGRGSGDPGKKKQHICHIQGCGKV
YGKTSHLRAHLRWHTGERPFMCNWSYCGKRFTRSDELQRHKRTHTGEKKFACPECPKRFMRSDHLSKHI
KTHQNKKGGPGVALSVGTLPLDSGAGSEGSGTATPSALITTNMVAMEAICPEGIARLANSGINVMQVTELQ
SINISGNGF
>T01040
MKEEPLGSGMNAVRTWMQGAGVLDANTAAQSGVGLARAHFEKQPPSNLRKSNFFHFVLALYDRQGQPV
EIERTAFVGFVEKEKEANSEKTNNGIHYRLQLLYSNGIRTEQDFYVRLIDSMTKQAIVYEGQDKNPEMCRV
LLTHEIMCSRCCDKKSCGNRNETPSDPVIIDRFFLKFFLKCNQNCLKNAGNPRDMRRFQVVVSTTVNVDG
HVLAVSDNMFVHNNSKHGRRARRLDPSEAATPCIKAISPSEGWTTGGATVIIIGDNFFDGLQVIFGTMLVW
SELITPHAIRVQTPPRHIPGVVEVTLSYKSKQFCKGTPGRFIYTALNEPTIDYGFQRLQKVIPRHPGDPERLP
KEVILKRAADLVEALYGMPHNNQEIILKRAADIAEALYSVPRNHNQLPALANTSVHAGMMGVNSFSGQLA
VNVSEASQATNQGFTRNSSSVSPHGYVPSTTPQQTNYNSVTTSMNGYGSAAMSNLGGSPTFLNGSAANSP
YAIVPSSPTMASSTSLPSNCSSSSGIFSFSPANMVSAVKQKSAFAPVVRPQTSPPPTCTSTNGNSLQAISGMIV
PPM
>T01562
MDCTFEDMLQLINNQDSDFPGLFDAPYAGGETGDTGPSSPGASSPESFSSPASLGSSLEAFLGGPKVTPAPL
SPPPSAPTAVKMYPSVPPFSPGPGIKEEPVPLTILQPPAPQPSPGTLLPPSFPPPPVQLSPAPVLGYSSLPSGFSG
TLPGNTQQTPSSLPLGSTPGISPTPLHTQVQSSAAQQPPPASAAPRMSTVASQIQQVPVVLQPHFIKADSLLL
TAVKTDTGATMKTAGINTLAPGTAVQAGPLQTLVSGGTILATVPLVVDTDKLPIHRLAAGGKALGSAQSRG
EKRTAHNAIEKRYRSSINDKIVELKDLVVGTEAKLNKSAVLRKAIDYIRFLQHSNQKLKQENLTLRSAHKS
KSLKDLVSACGSGGGTDVSMEGMKPEVVETLTPPPSDAGSPSQSSPLSLGSRGSSSGGSDSEPDSPAFEDN
QVKAQRLPSHSRGMLDRSRLALCVLVFLCLTCNPLASLFGWGILTPSDASGVHRSSGRSMLEAESRDGSN
WTQWLLPPLVWLANGLLVLACLALLFVYGEPVTRPHSGPAVHFWRHRKQADLDLARGDFAQAAQQLWL
ALQALGRPLPTSNLDLACSLLWNLVRHLLQRLWVGRWLAGQAGGLQRDYRLRKDARASARDAAVVYH
KLHQLHAMGKYTGGHLVASNLALSALNLAECAGDAISMATLAEIYVAAALRVKTSLPRALHFLTRFFLSS

ARQACLAQSGAVPLAMQWLCHPVGHRFFVDGDWAVHGAPQESLYSMAGNPVDPLAQVTRLFCEHLLER
ALNCIAQPSPGAADGDREFSDALGYLQLLNSCSDAVGAPACSFSVSSSMATTTGTDPVAKWWASLTAVVIH
WLRRDEEAAERLYPLVEHIPQVLQETERPLPRAALYSFKAARALLDHRKVESSPASLAICEKASGYLRDSL
ASTSTASSIDKAMQLLLCDLLLVARTSLWRRQQSAASAQGAHGTSNGPQASALELRGFQHDLSSLRRLA
>T02115
QAAFGDHNIQYQFRTESNGGQVTYRVVQVTDGQLDGQGDTAGAVSVVSTAAFAGGQQAVTQVGVDGAA
QRPGPAAASVPTGPAAPFPLAVIQNPFSNGGSPAAEAVSGEARFAYFPASSVGDTTAVSVQTTDQSLQAGGQ
FYVMMTPQDVLQTGTQRTIAPRTHPYSPKIDGTRTPRDERRRAQHNEVERRRRDKINNWIVQLSKIIPDCH
ADNSKTGASKGGILSKACDYIRELRQTNQRMQETFKEAERLQMDNELLRQQIEELKNENALLRAQLQQH
NLEMVGESTRQ
>T02716
MYQSLAMAANHGPPPGAYEAGGPGAFMHSAGAASSPVYVPTPRVPSSVLGLSYLQGGGSGAASGATSGG
SSGAGPSGAGPGTQQGSPGWSQAGAEGAAYTPPPVSPRFSFPGTTGSLAAAAAAAAAREAAAYSSSGGA
AGAGLAGREQYGRPGFAGSYSSPYPAYMADVGASWAAAAAAASAGPFDSPVLHSLPGRANPARHPNLDM
FDDFSEGRECVNCGAMSTPLWRRDGTGHYLCNACGLYHKMNGINRPLIKPQRRLSASRRVGLSCANCQT
TTTTLWRRNAEGEPVCNACGLYMKLHGVPRPLAMRKEGIQTRKRKPKNLNKSKTPAGPPGESLPPSSGAS
SNSSNATSSSSSSEEMRPIKTEPGLSSHYGHSSSMSQTFSTVSGHGSSIHPVLSALKLSPQGYPSPVTQTSQA
SSKQDSWNSLVLADSHGDIITA
>T02815
MIGDGCDLGLGEEEGGTGLPYPCQFCDKSFIRLSYLKRHEQIHSDKLPFKCTFCSRLFKHKRSRDRHIKLH
TGDKKYHCHECEAAFSRRDHLKIHLKTHSSSKPFKCSVCKRGFSSTSSLQSHMQAHKKNKEHLAKSEKEA
KKDDFMCDYCEDTFSQTEELEKHVLTLHPQLSEKADLQCIHCPEVFVDESTLLAHIHQAHANQKHKCPM
CPEQFSSVEGVYCHLDSHRQPDSSNHSVSPDPVLGSVASMSSATPDSTPDPVLGSVASMSSATPDSSASVER
GSTPDSTLKPLRGQKKMRDDGQSWSKVVYSCPYCSKRDFTSLAVLEIHLKTIHADKPQQSHTCQICLDSM
PTLYNLNEHVRKLHKSHAYPVMQFGNISAFHCNYCPEMFADINSLQEHIRVSHCGPNANPPDGNNAFFCN
QCSMGFLTESSLTEHIQQAHCSVGSTKLESPVIQPTQSFMEVYSCPYCTNSPIFGSILKLTKHIKENHKNIPLA
HSKKSKAEQSPVSSDVEVSSPKRQRLSGSANSISNGEYPCNQCDLKFSNFESFQTHLKLHLELLLRKQACP
QCKEDFDSQESLLQHLTVHYMTTSTHYVCESCDKQFSSVDDLQKHLLDMHTFVLYHCTLCQEVFDSKVSI
QVHLAVKHSNEKKMYRCTACNWDFRKEADLQVHVKHSHLGNPAKAHKCIFCGETFSTEVELQCHITTHS
KKYNCRFCSKAFHAVLLLEKHLREKHCVFDPAAENGTANGVPPTSTKKAEPADLQGMLLKNPEAPNSHE
ASEDDVDASEPMYGCDICGAAYTMEVLLQNHRLRDHNIRPGEDDGSRKKAEFIKGSHKCNVCSRTFFSEN
GLREHLQTHRGPAKHYMCPICGERFPSLLTLTEHKVTHSKSLDTGTCRICKMPLQSEEEFIEHCQMHPDLR
NSLTGFRCVVCMQTVTSTLELKIHGTFHMQKLAGSSAASSPNGQGLQKLYKCALCLKEFRSKQDLVRLDV
NGLPYGLCAGCMARSANGQVGGLAPPEPADRPCAGLRCPECNVKFESAEDLESHMQVDHRDLTPETSGP
RKGAQTSPVPRKKTYQCIKCQMTFENEREIQIHVANHMIEEGINHECKLCNQMFDSPAKLLCHLIEHSFEG
MGGTFKCPVCFTVFVQANKLQQHIFAVHGQEDKIYDCSQCPQKFFFQTELQNHTMSQHAQ
>T03257
MNAQLTMEAIGELHGVSHEPVPAPADLLGGSPHARSSVGHRGSHLPPAHPRSMGMASLLDGGSGGSDYH
HHHRAPEHSLAGPLHPTMTMACETPPGMSMPTTYTTLTPLQPLPPISTVSDKFPHHHHHHHHHHHHPHHHQ
RLAGNVSGSFTLMRDERGLASMNNLYTPYHKDVAGMGQSLSPLSGSGLGSIHNSQQGLPHYAHPGAAMP
TDKMLTPNDFEAHHPAMLGRHGEQHLTPTSAGMVPINGLPPHHPHAHLNAQGHGQLLGTAREPNPSVTG
AQVSNGSNSGQMEEINTKEVAQRITTELKRYSIPQAIFAQRVLCRSQGTLSDLLRNPKPWSKLKSGRETFRR
MWKWLQEPEFQRMSALRLAACKRKEQEHGKDRGNTPKKPRLVFTDVQRRTLHAIFKENKRPSKELQITIS
QQLGLELSTVSNFFMNARRRSLDKWQDEGSSNSGNSSSSSSTCTKA

>T03258
MNAQLTMEAIGELHGVSHEPVPAPADLLGGSPHARSSVGHRGSHLPPAHPRSMGMASLLDGGSGGSDYH
HHHRAPEHSLAGPLHPTMTMACETPPGMSMPTTYTTLTPLQPLPPISTVSDKFPHHHHHHHHHHHPHHHQ
RLAGNVSGSFTLMRDERGLASMNNLYTPYHKDVAGMGQSLSPLSGSGLGSIHNSQQGLPHYAHPGAAMP
TDKMLTPNDFEAHHPAMLGRHGEQHLTPTSAGMVPINGLPPHHPHAHLNAQGHGQLLGTAREPNPSVTG
AQVSNGSNSGQMEEINTKEVAQRITTELKRYSIPQAIFAQRVLCRSQGTLSDLLRNPKPWSKLKSGRETFRR
MWKWLQEPEFQRMSALRLAESAMGGSVPSLRITSGGPQLSVPPLPACKRKEQEHGKDRGNTPKKPRLVF
TDVQRRTLHAIFKENKRPSKELQITISQQLGLELSTVSNFFMNARRRSLDKWQDEGSSNSGNSSSSSSTCTK
A
>T04297
MLAVGAMEGPRQSAFLLSSPPLAAHLHSMAEMKTPLYPAAYPPLPTGPPSSSSSSSSSSSPSPPLGAHNPGGL
KPPAAGGLSSLGSPPQQLSAATPHGINDILSRPSMPVASGAALPSASPSGSSSSSSSSASATSASAAAAAAAA
AAAAAASSPAGLLAGLPRFSSLSPPPPPPGLYFSPSAAAVAAVGRYPKPLAELPGRTPIFWPGVMQSPPWRD
ARLACTPHQGSILLDKDGKRKHTRPTFSGQQIFALEKTFEQTKYLAGPERARLAYSLGMTESQVKVWFQN
RRTKWRKKHAAEMATAKKKQDSETERLKGTSENEEDDDDYNKPLDPNSDDEKITQLLKKHKSSGGSLLL
HASEAEGSS
>T05137
MEESHFNSNPYFWPSIPTVSGQIENTMFINKMKDQLLPEKGCGLAPPHYPTLLTVPASVSLSSGISMDTESK
SEQLTPHSQASVTQNITVVPVPSTGLMTAGVSCSQRWRREGSQSRGPGLVITSPSGSLVTTASSAQTFPISTP
MIVSALPPGSQALQVVPDLSKKVASTLTEEGGRSGGGGGTVAPPKPPRGRKKKRMLESGLPEMNDPYVLA
PGDDDDHQKDGKTYRSEGNCGTGNGQSLGLMDSVPGSTTNLLCDPGCRMCSLTFYSKSEMQIHSKSHTE
TKPHKCPHCSKTFANSSYLAQHIRIHSGAKPYSCNFCEKSFRQLSHLQQHTRIHTGDRPYKCAHPGCEKAF
TQLSNLQSHRRQHNKDKPFKCHNCHRAYTDAASLEAHLSTHTVKHAKVYTCTICSRAYTSETYLMKHMR
KHNPPDLQQQVQAAAAAAAVAQAQAQAQAQAQAQAQAQAQAQAQAQASQASQQQQQQPPPPQP
PHFQSPGAAPQGGGGGDSNQNPPPQCSFDLTPYKPAEHHKDICLTVTTSTIQVEHWPSS
>T00035
MLWKLTDNIKYEDCEDRHDGTSNGTARLPQLGTVGQSPYTSAPPLSHTPNADFQPPYFPPPYQPIYPQSQD
PYSHVNDPYSLNPLHAQPQPQHPGWPGQRQSQESGLLHTHRGLPHQLSGLDPRRDYRRHEDLLHGPHAL
SSGLGDLSIHSLPHAIEEVPHVEDPGINIPDQTVIKKGPVSLSKSNSNAVSAIPINKDNLFGGVVNPNEVFCS
VPGRLSLLSSTSKYKVTVAEVQRRLSPPECLNASLLGGVLRRAKSKNGGRSLREKLDKIGLNLPAGRRKA
ANVTLLTSLVEGEAVHLARDFGYVCETEFPAKAVAEFLNRQHSDPNEQVTRKNMLLATKQICKEFTDLLA
QDRSPLGNSRPNPILEPGIQSCLTHFNLISHGFGSPAVCAAVTALQNYLTEALKAMDKMYLSNNPNSHTDN
NAKSSDKEEKHRK
>T00036
RKTEKEVIGGLCSLANIPLTPETQRDQERRIRREIANSNERRRMQSINAGFQSLKTLIPHTDGEKLSKAAILQ
QTAEYIFSLEQEKTRLLQQNTQLKRFIQELSGSSPKRRRAEDKDEGIGSPDIWEDEKAEDLRREMIELRQQL
DKERSVRMMLEEQVRSLEAHMYPEKLKVIAQQVQLQQQQEQVRLLHQEKLEREQQQLRTQLLPPPAPTH
HPTVIVPAPPPPPSHHINVVTMGPSSVINSVSTSRQNLDTIVQAIQHIEGTQEKQELEEEQRRAVIVKPVRSCP
EAPTSDTASDSEASDSDAMDQSREEPSGDGELP
>T00040
MEVQLGLGRVYPRPPSKTYRGAFQNLFQSVREVIQNPGPRHPEAASAAPPGASLLLLQQQQQQQQQQQ
QQQQQQQQETSPRQQQQQQGEDGSPQAHRRGPTGYLVLDEEQQPSQPQSALECHPERGCVPEPGAAVA
ASKGLPQQLPAPPDEDDSAAPSTLSLLGPTFPGLSSCSADLKDILSEASTMQLLQQQQQEAVSEGSSSGRAR
EASGAPTSSKDNYLGGTSTISDNAKELCKAVSVSMGLGVEALEHLSPGEQLRGDCMYAPLLGVPPAVRPT

PCAPLAECKGSLLDDSAGKSTEDTAEYSPFKGGYTKGLEGESLGCSGSAAAGSSGTLELPSTLSLYKSGAL
DEAAAYQSRDYYNFPLALAGPPPPPPPPHPHARIKLENPLDYGSAWAAAAAQCRYGDLASLHGAGAAGP
GSGSPSAAASSSWHTLFTAEEGQLYGPCGGGGGGGGGGGGGGGGGGGGGGEAGAVAPYGYTRPPQG
LAGQESDFTAPDVWYPGGMVSRVPYPSPTCVKSEMGPWMDSYSGPYGDMRLETARDHVLPIDYYFPPQK
TCLICGDEASGCHYGALTCGSCKVFFKRAAEGKQKYLCASRNDCTIDKFRRKNCPSCRLRKCYEAGMTL
GARKLKKLGNLKLQEEGEASSTTSPTEETTQKLTVSHIEGYECQPIFLNVLEAIEPGVVCAGHDNNQPDSF
AALLSSLNELGERQLVHVVKWAKALPGFRNLHVDDQMAVIQYSWMGLMVFAMGWRSFTNVNSRMLYF
APDLVFNEYRMHKSRMYSQCVRMRHLSQEFGWLQITPQEFLCMKALLLFSIIPVDGLKNQKFFDELRMN
YIKELDRIIACKRKNPTSCSRRFYQLTKLLDSVQPIARELHQFTFDLLIKSHMVSVDFPEMMAEIISVQVPKI
LSGKVKPIYFHTQ
>T00045
MAMVVSTWRDPQDEVPGSQGSQASQAPPVPGPPPGAPHTPQTPGQGGPASTPAQTAAGGQGGPGGPGSD
KQQQQQHIECVVCGDKSSGKHYGQFTCEGCKSFFKRSVRRNLSYTCRANRNCPIDQHHRNQCQYCRLKK
CLKVGMRREAVQRGRMPPTQPTHGQFALTNGDPLNCHSYLSGYISLLLRAEPYPTSRFGSQCMQPNNIMG
IENICELAARMLFSAVEWARNIPFFPDLQITDQVALLRLTWSELFVLNAAQCSMPLHVAPLLAAAGLHASP
MSADRVVAFMDHIRIFQEQVEKLKALHVDSAEYSCLKAIVLFTSDACGLSDVAHVESLQEKSQCALEEYV
RSQYPNQPTRFGKLLLRLPSLRTVSSSVIEQLFFVRLVGKTPIETLIRDMLLSGSSFNWPYMAIQ
>T00100
MLCVRGARLKRELDATATVLANRQDESEQSRKRLIEQSREFKKNTPEDLRKQVAPLLKSFQGEIDALSKRS
KEAEAAFLNVYKRLIDVPDPVPALDLGQQLQLKVQRLHDIETENQKLRETLEEYNKEFAEVKNQEVTIKA
LKEKIREYEQTLKNQAETIALEKEQKLQNDFAEKERKLQETQMSTTSKLEEAEHKVQSLQTALEKTRTELF
DLKTKYDEETTAKADEIEMIMTDLERANQRAEVAQREAETLREQLSSANHSLQLASQIQKAPDVEQAIEVL
TRSSLEVELAAKEREIAQLVEDVQRLQASLTKLRENSASQISQLEQQLSAKNSTLKQLEEKLKGQADYEEV
KKELNILKSMEFAPSEGAGTQDAAKPLEVLLLEKNRSLQSENAALRISNSDLSGSARRKGKDQPESRRPGS
LPAPPPSQLPRNPGEQASNTNGTHQFSPAGLSQDFFSSSLASPSLPLASTGKFALNSLLQRQLMQSFYSKAM
QEAGSTSMIFSTGPYSTNSISSQSPLQQSPDVNGMAPSPSQSESAGSVSEGEEMDTAEIARQVKEQLIKHNI
GQRIFGHYVLGLSQGSVSEILARPKPWNKLTVRGKEPFHKMKQFLSDEQNILALRSIQGRQRENPGQSLNR
LFQEVPKRRNGSEGNITTRIRASETGSDEAIKSILEQAKRELQVQKTAEPAQPSSASGSGNSDEPIRSILQQA
RREMEAQQAALDPALKQAPLSQSDITILTPKLLSTSPMPTVSSYPPLAISLKKPSAAPEAGASALPNPPALKK
EAQDAPGLDPQGAADCAQGVLRQVKNEVGRSGAWKDHWWSAVQPERRNAASSEEAKARETGGGKEK
GSGGSGGGSQPRAERSQLQGPSSSEYWKEWPSAESPYSQSSELSLTGASRSETPQNSPLPSSPIVPMSKPTK
PSVPPLTPEQYEVYMYQEVDTIELTRQVKEKLAKNGICQRIFGEKVLGLSQGSVSDMLSRPKPWSKLTQKG
REPFIRMQLWLNGELGQGVLPVQGQQQGPVLHSVTSLQDPLQQGCVSSESTPKTSASCSPAPESPMSSSES
VKSLTELVQQPCPPIEASKDSKPPEPSDPPASDSQPTTPLPLSGHSALSIQELVAMSPELDTYGITKRVKEVLT
DNNLGQRLFGETILGLTQGSVSDLLARPKPWHKLSLKGREPFVRMQLWLNDPNNVEKLMDMKRMEKKA
YMKRRHSSVSDSQPCEPPSVGTEYSQGASPQPQHQLKKPRVVLAPEEKEALKRAYQQKPYPSPKTIEDLAT
QLNLKTSTVINWFHNYRSRIRRELFIEEIQAGSQGQAGASDSPSARSGRAAPSSEGDSCDGVEATEGPGSA
DTEEPKSQGEAEREEVPRPAEQTEPPPSGTPGPDDARDDDHEGGPVEGPGPLPSPASATATAAPAAPEDAAT
SAAAAPGEGPAAPTSAPPPSNSSSSSAPRRPSSLQSLFGLPEAAGARDSRDNPLRKKKAANLNSIIHRLEKA
ASREEPIEWEF
>T00123
MMFSGFNADYEASSSRCSSASPAGDSLSYYHSPADSFSSMGSPVNAQDFCTDLAVSSANFIPTVTAISTSPD
LQWLVQPALVSSVAPSQTRAPHPFGVPAPSAGAYSRAGVVKTMTGGRAQSIGRRGKVEQLSPEEEEKRRIR
RERNKMAAAKCRNRRRELTDTLQAETDQLEDEKSALQTEIANLLKEKEKLEFILAAHRPACKIPDDLGFPE

EMSVASLDLTGGLPEVATPESEEAFTLPLLNDPEPKPSVEPVKSISSMELKTEPFDDFLFPASSRPSGSETARS
VPDMDLSGSFYAADWEPLHSGSLGMGPMATELEPLCTPVVTCTPSCTAYTSSFVFTYPEADSFPSCAAAHR
KGSSSNEPSSDSLSSPTLLAL
>T00133
MTAKMETTFYDDALNASFLPSESGPYGYSNPKILKQSMTLNLADPVGSLKPHLRAKNSDLLTSPDVGLLK
LASPELERLIIQSSNGHITTTPTPTQFLCPKNVTDEQEGFAEGFVRALAELHSQNTLPSVTSAAQPVNGAGM
VAPAVASVAGGSGSGGFSASLHSEPPVYANLSNFNPGALSSGGGAPSYGAAGLAFPAQPQQQQQPPHHLPQ
QMPVQHPRLQALKEEPQTVPEMPGETPPLSPIDMESQERIKAERKRMRNRIAASKCRKRKLERIARLEEKV
KTLKAQNSELASTANMLREQVAQLKQKVMNHVNSGCQLMLTQQLQTF
>T00140
MPLNVSFTNRNYDLDYDSVQPYFYCDEEENFYQQQQQSELQPPAPSEDIWKKFELLPTPPLSPSRRSGLCS
PSYVAVTPFSLRGDNDGGGGSFSTADQLEMVTELLGGDMVNQSFICDPDDETFIKNIIIQDCMWSGFSAAA
KLVSEKLASYQAARKDSGSPNPARGHSVCSTSSLYLQDLSAAASECIDPSVVFPYPLNDSSSPKSCASQDSS
AFSPSSDSLLSSTESSPQGSPEPLVLHEETPPTTSSDSEEEQEDEEEIDVVSVEKRQAPGKRSESGSPSAGGHS
KPPHSPLVLKRCHVSTHQHNYAAPPSTRKDYPAAKRVKLDSVRVLRQISNNRKCTSPRSSDTEENVKRRTH
NVLERQRRNELKRSFFALRDQIPELENNEKAPKVVILKKATAYILSVQAEEQKLISEEDLLRKRREQLKHKL
EQLRNSCA
>T00149
MAMVVSSWRDPQDDVAGGNPGGPNPAAQAARGGGGGAGEQQQQAGSGAPHTPQTPGQPGAPATPGTA
GDKGQGPPGSGQSQQHIECVVCGDKSSGKHYGQFTCEGCKSFFKRSVRRNLTYTCRANRNCPIDQHHRN
QCQYCRLKKCLKVGMRREAVQRGRMPPTQPNPGQYALTNGDPLNGHCYLSGYISLLLRAEPYPTSRYGS
QCMQPNNIMGIENICELAARLLFSAVEWARNIPFFPDLQITDQVSLLRLTWSELFVLNAAQCSMPLHVAPLL
AAAGLHASPMSADRVVAFMDHIRIFQEQVEKLKALHVDSAEYSCLKAIVLFTSDACGLSDAAHIESLQEK
SQCALEEYVRSQYPNQPSRFGKLLLRLPSLRTVSSSVIEQLFFVRLVGKTPIETLIRDMLLSGSSFNWPYMSI
QCS
>T00163
MTMESGAENQQSGDAAVTEAENQQMTVQAQPQIATLAQVSMPAAHATSSAPTVTLVQLPNGQTVQVHG
VIQAAQPSVIQSPQVQTVQSSCKDLKRLFSGTQISTIAESEDSQESVDSVTDSQKRREILSRRPSYRKILNDL
SSDAPGVPRIEEEKSEEETSAPAITTVTVPTPIYQTSSGQYIAITQGGAIQLANNGTDGVQGLQTLTMTNAA
ATQPGTTILQYAQTTDGQQILVPSNQVVVQAASGDVQTYQIRTAPTSTIAPGVVMASSPALPTQPAEEAAR
KREVRLMKNREAARECRRKKKEYVKCLENRVAVLENQNKTLIEELKALKDLYCHKSD
>T00168
MASGAYNPYIEIIEQPRQRGMRFRYKCEGRSAGSIPGEHSTDNNRTYPSIQIMNYYGKGKVRITLVTKNDP
YKPHPHDLVGKDCRDGYYEAEFGQERRPLFFQNLGIRCVKKKEVKEAIITRIKAGINPFNVPEKQLNDIED
CDLNVVRLCFQVFLPDEHGNLTTALPPVVSNPIYDNRAPNTAELRICRVNKNCGSVRGGDEIFLLCDKVQK
DDIEVRFVLNDWEAKGIFSQADVHRQVAIVFKTPPYCKAITEPVTVKMQLRRPSDQEVSESMDFRYLPDE
KDTYGNKAKKQKTTLLFQKLCQDHVETGFRHVDQDGLELLTSGDPPTLASQSAGITVNFPERPRPGLLGSI
GEGRYFKKEPNLFSHDAVVREMPTGVSSQAESYYPSPGPISSGLSHHASMAPLPSSSWSSVAHPTPRSGNT
NPLSSFSTRTLPSNSQGIPPFLRIPVGNDLNASNACIYNNADDIVGMEASSMPSADLYGISDPNMLSNCSVN
MMTTSSDSMGETDNPRLLSMNLENPSCNSVLDPRDLRQLHQMSSSSMSAGANSNTTVFVSQSDAFEGSD
FSCADNSMINESGPSNSTNPNSHGFVQDSQYSGIGSMQNEQLSDSFPYEFFQV
>T00204
MNQPQRMAPVGTDKELSDLLDFSMMFPLPVTNGKGRPASLAGAQFGGSGLEDRPSSGSWGSGDQSSSSF
DPSRTFSEGTHFTESHSSLSSSTFLGPGLGGKSGERGAYASFGRDAGVGGLTQAGFLSGELALNSPGPLSPS

GMKGTSQYYPSYSGSSRRRAADGSLDTQPKKVRKVPPGLPSSVYPPSSGEDYGRDATAYPSAKTPSSTYPA
PFYVADGSLHPSAELWSPPGQAGFGPMLGGGSSPLPLPPGSGPVGSSGSSSTFGGLHQHERMGYQLHGAE
VNGGLPSASSFSSAPGATYGGVSSHTPPVSGADSLLGSRGTTAGSSGDALGKALASIYSPDHSSNNFSSSPS
TPVGSPQGLAGTSQWPRAGAPGALSPSYDGGLHGLQSKIEDHLDEAIHVLRSHAVGTAGDMHTLLPGHG
ALASGFTGPMSLGGRHAGLVGGSHPEDGLAGSTSLMHNHAALPSQPGTLPDLSRPPDSYSGLGRAGATAA
ASEIKREEKEDEENTSAADHSEEEKKELKAPRARTSPDEDEDDLLPPEQKAEREKERRVANNARERLRVRD
INEAFKELGRMCQLHLNSEKPQTKLLILHQAVSVILNLEQQVRERNLNPKAACLKRREEEKVSGVVGDPQ
MVLSAPHPGLSEAHNPAGHM
>T00241
MAAAKAEMQLMSPLQISDPFGSFPHSPTMDNYPKLEEMMLLSNGAPQFLGAAGAPEGSGSNSSSSSSGGG
GGGGGGSNSSSSSSTFNPQADTGEQPYEHLTAESFPDISLNNEKVLVETSYPSQTTRLPPITYTGRFSLEPAP
NSGNTLWPEPLFSLVSGLVSMTNPPASSSSAPSPAASSASASQSPPLSCAVPSNDSSPIYSAAPTFPTPNTDIFP
EPQSQAFPGSAGTALQYPPPAYPAAKGGFQVPMIPDYLFPQQQGDLGLGTPDQKPFQGLESRTQQPSLTPLS
TIKAFATQSGSQDLKALNTSYQSQLIKPSRMRKYPNRPSKTPPHERPYACPVESCDRRFSRSDELTRHIRIHT
GQKPFQCRICMRNFSRSDHLTTHIRTHTGEKPFACDICGRKFARSDERKRHTKIHLRQKDKKADKSVVASS
ATSSLSSYPSPVATSYPSPVTTSYPSPATTSYPSPVPTSFSSPGSSTYPSPVHSGFPSPSVATTYSSVPPAFPAQV
SSFPSSAVTNSFSASTGLSDMTATFSPRTIEIC
>T00423
MPITWMRMRPWLEMQINSNQIPGLIWINKEEMILEIPWKHAAKHGWDINKDACLFRSWAIHTGRYKAGE
KEPDPKTWKANFRCAMNSLPDIEEVKDQSRNKGSSAVRVYRMLPPLTKNQRKERKSKSSRDAKSKAKRK
SCGDSSPDTFSDGLSSSTLPDDHSSYTVPGYMQDLEVEQALTPALSPCAVSSTLPDWHIPVEVVPDSTSDLY
NFQVSPMPSISEATTDEDEEGKLPEDIMKLLEQSEWQPTNVDGKGYLLNEPGVQPTSVYGDFSCKEEPEID
SPGGDIGLSLQRVFTDLKNMDATWLDSLLTPVRLPSIQAIPCAP
>T00490
MFPVFPCTLLAPPFPVLGLDSRGVGGLMNSFPPPQGHAQNPLQVGAELQSRFFASQGCAQSPFQAAPAPPP
TPQAPAAEPLQVDLLPVLAAAQESAAAAAAAAAAAAAVAAAPPAPAAASTVDTAALKQPPAPPPPPPPVS
APAAEAAPPASAATIAAAAATAVVAPTSTVAVAPVASALEKKTKSKGPYICALCAKEFKNGYNLRRHEAIHT
GAKAGRVPSGAMKMPTMVPLSLLSVPQLSGAGGGGGEAGAGGGAAAVAAGGVVTTTASGKRIRKNHAC
EMCGKAFRDVYHLNRHKLSHSDEKPYQCPVCQQRFKRKDRMSYHVRSHDGAVHKPYNCSHCGKSFSRP
DHLNSHVRQVHSTERPFKCEKCEAAFATKDRLRAHTVRHEEKVPCHVCGKMLSSAYISDHMKVHSQGPH
HVCELCNKGTGEVCPMAAAAAAAAAAAAAAVAAPPTAVGSLSGAEGVPVSSQPLPSQPW
>T00525
MELLSPPLRDVDLTAPDGSLCSFATTDDFYDDPCFDSPDLRFFEDLDPRLMHVGALLKPEEHSHFPAAVHP
APGAREDEHVRAPSGHHQAGRCLLWACKACKRKTTNADRRKAATMRERRRLSKVNEAFETLKRCTSSN
PNQRLPKVEILRNAIRYIEGLQALLRDQDAAPPGAAAFYAPGPLPPGRGGEHYSGDSDASSPRSNCSDGM
MDYSGPPSGARRRNCYEGAYYNEAPSEPRPGKSAAVSSLDYLSSIVERISTESPAAPALLLADVPSESPPRR
QEAAAPSEGESSGDPTQSPDAAPQCPAGANPNPIYQVL
>T00594
MDELFPLIFPAEPAQASGPYVEIIEQPKQRGMRFRYKCEGRSAGSIPGERSTDTTKTHPTIKINGYTGPGTVR
ISLVTKDPPHRPHPHELVGKDCRDGFYEAELCPDRCIHSFQNLGIQCVKKRDLEQAISQRIQTNNNPFQVPI
EEQRGDYDLNAVRLCFQVTVRDPSGRPLRLPPVLPHPIFDNRAPNTAELKICRVNRNSGSCLGGDEIFLLCD
KVQKEDIEVYFTGPGWEARGSFSQADVHRQVAIVFRTPPYADPSLQAPVRVSMQLRRPSDRELSEPMEFQ
YLPDTDDRHRIEEKRKRTYETFKSIMKKSPFSGPTDPRPPPRRIAVPSRSSASVPKPAPQPYPFTSSLSTINYD
EFPTMVFPSGQISQASALAPAPPQVLPQAPAPAPAPAMVSALAQAPAPVPVLAPGPPQAVAPPAPKPTQAGE

GTLSEALLQLQFDDEDLGALLGNSTDPAVFTDLASVDNSEFQQLLNQGIPVAPHTTEPMLMEYPEAITRLV
TGAQRPPDPAPAPLGAPGLPNGLLSGDEDFSSIADMDFSALLSQISS
>T00630
MATAASNHYSLLTSSASIVHAEPPGAMQQGAGGYREAQSLVQGDYGALQSNGHPLSHAHQWITALSHGG
GGGGGGGGGGGGGGGGGGGDGSPWSTSPLGQPDIKPSVVVQQGGRGDELHGPGALQQQHQQQQQQQQ
QQQQQQQQQQQQQRPPHLVHHAANHHPGPGAWRTAAAAAHLPPSMGASNGGLLYSQPSFTVNGMLGA
GGQPAGLHHHGLRDAHDEPHHADHHPHPHSHPHQQPPPPPPPQGPPGHPGAHHDPHSDEDTPTSDDLEQF
AKQFKQRRIKLGFTQADVGLALGTLYGNVFSQTTICRFEALQLSFKNMCKLKPLLNKWLEEADSSSGSPT
SIDKIAAQGRKRKKRTSIEVSVKGALESHFLKCPKPSAQEITSLADSLQLEKEVVRVWFCNRRQKEKRMTP
PGGTLPGAEDVYGGSRDTPPHHGVQTPVQ
>T00671
MEEPQSDPSVEPPLSQETFSDLWKLLPENNVLSPLPSQAMDDLMLSPDDIEQWFTEDPGPDEAPRMPEAAP
PVAPAPAAPTPAAPAPAPSWPLSSSVPSQKTYQGSYGFRLGFLHSGTAKSVTCTYSPALNKMFCQLAKTCP
VQLWVDSTPPPGTRVRAMAIYKQSQHMTEVVRRCPHHERCSDSDGLAPPQHLIRVEGNLRVEYLDDRNT
FRHSVVVPYEPPEVGSDCTTIHYNYMCNSSCMGGMNRRPILTIITLEDSSGNLLGRNSFEVRVCACPGRDR
RTEEENLRKKGEPHHELPPGSTKRALPNNTSSSPQPKKKPLDGEYFTLQIRGRERFEMFRELNEALELKDA
QAGKEPGGSRAHSSHLKSKKGQSTSRHKKLMFKTEGPDSD
>T00719
MASNSSSCPTPGGGHLNGYPVPPYAFFFPPMLGGLSPPGALTTLQHQLPVSGYSTPSPATIETQSSSSEEIVPS
PPSPPPLPRIYKPCFVCQDKSSGYHYGVSACEGCKGFFRRSIQKNMVYTCHRDKNCIINKVTRNRCQYCRL
QKCFEVGMSKESVRNDRNKKKKEVPKPECSESYTLTPEVGELIEKVRKAHQETFPALCQLGKYTTNNSSE
QRVSLDIDLWDKFSELSTKCIIKTVEFAKQLPGFTTLTIADQITLLKAACLDILILRICTRYTPEQDTMTFSDG
LTLNRTQMHNAGFGPLTDLVFAFANQLLPLEMDDAETGLLSAICLICGDRQDLEQPDRVDMLQEPLLEAL
KVYVRKRRPSRPHMFPKMLMKITDLRSISAKGAERVITLKMEIPGSMPPLIQEMLENSEGLDTLSGQPGGG
GRDGGGLAPPPGSCSPSLSPSSNRSSPATHSP
>T00721
MFDCMDVLSVSPGQILDFYTASPSSCMLQEKALKACFSGLTQTEWQHRHTAQSIETQSTSSEELVPSPPSPL
PPPRVYKPCFVCQDKSSGYHYGVSACEGCKGFFRRSIQKNMIYTCHRDKNCVINKVTRNRCQYCRLQKC
FEVGMSKESVRNDRNKKKKETSKQECTESYEMTAELDDLTEKIRKAHQETFPSLCQLGKYTTNSSADHRV
RLDLGLWDKFSELATKCIIKIVEFAKRLPGFTGLTIADQITLLKAACLDILILRICTRYTPEQDTMTFSDGLTL
NRTQMHNAGFGPLTDLVFTFANQLLPLEMDDTETGLLSAICLICGDRQDLEEPTKVDKLQEPLLEALKIYIR
KRRPSKPHMFPKILMKITDLRSISAKGAERVITLKMEIPGSMPPLIQEMLENSEGHEPLTPSSSGNTAEHSPSI
SPSSVENSGVSQSPLVQ
>T00764
MLPTQAGAAAALGRGSALGGSLNRTPTGRPGGGGGTRGANGGRVPGNGAGLGPGRLEREAAAAAATTP
APTAGALYSGSEGDSESGEEEELGAERRGLKRSLSEMEIGMVVGGPEASAAATGGYGPVSGAVSGAKPGK
KTRGRVKIKMEFIDNKLRRYTTFSKRKTGIMKKAYELSTLTGTQVLLLVASETGHVYTFATRKLQPMITSET
GKALIQTCLNSPDSPPRSDPTTDQRMSATGFEETDLTYQVSESDSSGETKDTLKPAFTVTNLPGTTSTIQTAP
STSTTMQVSSGPSFPITNYLAPVSASVSPSAVSSANGTVLKSTGSGPVSSGGLMQLPTSFTLMPGGAVAQQV
PVQAIQVHQAPQQASPSRDSSTDLTQTSSSGTVTLPATIMTSSVPTTVGGHMMYPSPHAVMYAPTSGLGDG
SLTVLNAFSQAPSTMQVSHSQVQEPGGVPQVFLTASSGTVQIPVSAVQLHQMAVIGQQAGSSSNLTELQVV
NLDTAHSTKSE
>T00794
MDQNNSLPPYAQGLASPQGAMTPGIPLFSPMMPYGTGLTPQPIQNTNSLSILEEQQRQQQQQQQQQQQQQ

QQQQQQQQQQQQQQQQQQQQQQQQQAVAAAAVQQSTSQQATQGTSGQAPQLFHSQTLTTAPLPGTTPL
YPSPMTPMTPITPATPASESSGIVPQLQNIVSTVNLGCKLDLKTIALRARNAEYNPKRFAAVIMRIREPRTTA
LIFSSGKMVCTGAKSEEQSRLAARKYARVVQKLGFPAKFLDFKIQNMVGSCDVKFPIRLEGLVLTHQQFSS
YEPELFPGLIYRMIKPRIVLLIFVSGKVVLTGAKVRAEIYEAFENIYPILKGFRKTT
>T00857
MSMSPKHTTPFSVSDILSPLEESYKKVGMEGGGLGAPLAAYRQGQAAPPTAAMQQHAVGHHGAVTAAYH
MTAAGVPQLSHSAVGGYCNGNLGNMSELPPYQDTMRNSASGPGWYGANPDPRFPAISRFMGPASGMNM
SGMGGLGSLGDVSKNMAPLPSAPRRKRRVLFSQAQVYELERRFKQQKYLSAPEREHLASMIHLTPTQVKI
WFQNHRYKMKRQAKDKAAQQQLQQDSGGGGGGGTGCPQQQQAQQQSPRRVAVPVLVKDGKPCQAG
APAPGAASLQGHAQQQAQHQAQAAQAAAAAISVGSGGAGLGAHPGHQPGSAGQSPDLAHHAASPAALQ
GQVSSLSHLNSSGSDYGTMSCSTLLYGRTW
>T00878
MDMLDPGLDPAASATAAAAASHDKGPEAEEGVELQEGGDGPGAEEQTAVAITSVQQAAFGDHNIQYQFR
TETNGGQVTYRVVQVTDGQLDGQGDTAGAVSVVSTAAFAGGQQAVTQVGVDGAAQRPGPAAASVPPGP
AAPFPLAVIQNPFSNGGSPAAEAVSGEARFAYFPASSVGDTTAVSVQTTDQSLQAGGQFYVMMTPQDVLQT
GTQRTIAPRTHPYSPKIDGTRTPRDERRRAQHNEVERRRRDKINNWIVQLSKIIPDCNADNSKTGASKGGIL
SKACDYIRELRQTNQRMQETFKEAERLQMDNELLRQQIEELKNENALLRAQLQQHNLEMVGEGTRQ
>T00885
MEAMAASTSLPDPGDFDRNVPRICGVCGDRATGFHFNAMTCEGCKGFFRRSMKRKALFTCPFNGDCRIT
KDNRRHCQACRLKRCVDIGMMKEFILTDEEVQRKREMILKRKEEEALKDSLRPKLSEEQQRIIAILLDAHH
KTYDPTYSDFCQFRPPVRVNDGGGSHPSRPNSRHTPSFSGDSSSSCSDHCITSSDMMDSSSFSNLDLSEEDS
DDPSVTLELSQLSMLPHLADLVSYSIQKVIGFAKMIPGFRDLTSEDQIVLLKSSAIEVIMLRSNESFTMDDMS
WTCGNQDYKYRVSDVTKAGHSLELIEPLIKFQVGLKKLNLHEEEHVLLMAICIVSPDRPGVQDAALIEAIQ
DRLSNTLQTYIRCRHPPPGSHLLYAKMIQKLADLRSLNEEHSKQYRCLSFQPECSMKLTPLVLEVFGNEIS
>T00929
MKMDMEDADMTLWTEAEFEEKCTYIVNDHPWDSGADGGTSVQAEASLPRNLLFKYATNSEEVIGVMSK
EYIPKGTRFGPLIGEIYTNDTVPKNANRKYFWRIYSRGELHHFIDGFNEEKSNWMRYVNPAHSPREQNLAA
CQNGMNIYFYTIKPIPANQELLVWYCRDFAERLHYPYPGELTMMNLTQTQSSLKQPSTEKNELCPKNVPK
REYSVKEILKLDSNPSKGKDLYRSNISPLTSEKDLDDFRRRGSPEMPFYPRVVYPIRAPLPEDFLKASLAYGI
ERPTYITRSPIPSSTTPSPSARSSPDQSLKSSSPHSSPGNTVSPVGPGSQEHRDSYAYLNASYGTEGLGSYPGY
APLPHLPPAFIPSYNAHYPKFLLPPYGMNCNGLSAVSSMNGINNFGLFPRLCPVYSNLLGGGSLPHPMLNP
TSLPSSLPSDGARRLLQPEHPREVLVPAPHSAFSFTGAAASMKDKACSPTSGSPTAGTAATAEHVVQPKATS
AAMAAPSSDEAMNLIKNKRNMTGYKTLPYPLKKQNGKIKYECNVCAKTFGQLSNLKVHLRVHSGERPF
KCQTCNKGFTQLAHLQKHYLVHTGEKPHECQVCHKRFSSTSNLKTHLRLHSGEKPYQCKVCPAKFTQFV
HLKLHKRLHTRERPHKCSQCHKNYIHLCSLKVHLKGNCAAAPAPGLPLEDLTRINEEIEKFDISDNADRLE
DVEDDISVISVVEKEILAVVRKEKEETGLKVSLQRNMGNGLLSSGCSLYESSDLPLMKLPPSNPLPLVPVKV
KQETVEPMDP
>T00997
MQSYASAMLSVFNSDDYSPAVQENIPALRRSSSFLCTESCNSKYQCETGENSKGNVQDRVKRPMNAFIVW
SRDQRRKMALENPRMRNSEISKQLGYQWKMLTEAEKWPFFQEAQKLQAMHREKYPNYKYRPRRKAKM
LPKNCSLLPADPASVLCSEVQLDNRLYRDDCTKATHSRMEHQLGHLPPINAASSPQQRDRYSHWTKL
>T01009
MGRKKIQITRIMDERNRQVTFTKRKFGLMKKAYELSVLCDCEIALIIFNSSNKLFQYASTDMDKVLLKYTE
YNEPHESRTNSDIVEALNKKEHRGCDSPDPDTSYVLTPHTEEKYKKINEEFDNMMRNHKIAPGLPPQNFS

MSVTVPVTSPNALSYTNPGSSLVSPSLAASSTLTDSSMLSPPQTTLHRNVSPGAPQRPPSTGNAGGMLSTT
DLTVPNGAGSSPVGNGFVNSRASPNLIGATGANSLGKVMPTKSPPPPGGGNLGMNSRKPDLRVVIPPSSKG
MMPPLNTQRISSSQATQPLATPVVSVTTPSLPPQGLVYSAMPTAYNTDYSLTSADLSALQGFNSPGMLSLG
QVSAWQQHHLGQAALSSLVAGGQLSQGSNLSINTNQNISIKSEPISPPRDRMTPSGFQQQQQQQQPPPPP
QPQPQPPQPQPRQEMGRSPVDSLSSSSSSYDGSDREDPRGDFHSPIVLGRPPNTEDRESPSVKRMRMDAW
VT
>T01122
MQNSHSGVNQLGGVFVNGRPLPDSTRQKIVELAHSGARPCDISRILQVSNGCVSKILGRYYETGSIRPRAIG
GSKPRVATPEVVSKIAQYKRECPSIFAWEIRDRLLSEGVCTNDNIPSVSSINRVLRNLASEKQQMGADGMY
DKLRMLNGQTGSWGTRPGWYPGTSVPGQPTQDGCQQQEGGGENTNSISSNGEDSDEAQMRLQLKRKLQ
RNRTSFTQEQIEALEKEFERTHYPDVFARERLAAKIDLPEARIQVWFSNRRAKWRREEKLRNQRRQASNTP
SHIPISSSFSTSVYQPIPQPTTPVSSFTSGSMLGLTDTALTNTYSALPPMPSFTMANNLPMQPPVPSQTSSYSC
MLPTSPSVNGRSYDTYTPPHMQTHMNSQPMGTSGTTSTGLISPGVSVPVQVPGSEPDMSQYWPRLQ
>T01313
MMLQHPGQVSASEVSASAIVPCLSPPGSLVFEDFANLTPFVKEELRFAIQNKHLCHRMSSALESVTVSDRPL
GVSITKAEVAPEEDERKKRRRERNKIAAAKCRNKKKEKTECLQKESEKLESVNAELKAQIEELKNEKQHL
IYMLNLHRPTCIVRAQNGRTPEDERNLFIQQIKEGTLQS
>T01462
MFRDFGEPGPSSGNGGGYGGPAQPPAAAQAAQQKFHLVPSINTMSGSQELQWMVQPHFLGPSSYPRPLTY
PQYSPPQPRPGVIRALGPPPGVRRRPCEQISPEEEERRRVRRERNKLAAAKCRNRRKELTDFLQAETDKLE
DEKSGLQREIEELQKQKERLELVLEAHRPICKIPEGAKEGDTGSTSGTSSPPAPCRPVPCISLSPGPVLEPEAL
HTPTLMTTPSLTPFTPSLVFTYPSTPEPCASAHRKSSSSSGDPSSDPLGSPTLLAL
>T01493
MAQWNQLQQLDTRYLEQLHQLYSDSFPMELRQFLAPWIESQDWAYAASKESHATLVFHNLLGEIDQQYS
RFLQESNVLYQHNLRRIKQFLQSRYLEKPMEIARIVARCLWEESRLLQTAATAAQQGGQANHPTAAVVTEK
QQMLEQHLQDVRKRVQDLEQKMKVVENLQDDFDFNYKTLKSQGDMQDLNGNNQSVTRQKMQQLEQM
LTALDQMRRSIVSELAGLLSAMEYVQKTLTDEELADWKRRQQIACIGGPPNICLDRLENWITSLAESQLQT
RQQIKKLEELHQKVSYKGDPIVQHRPMLEERIVELFRNLMKSAFVVERQPCMPMHPDRPLVIKTGVQFTT
KVRLLVKFPELNYQLKIKVCIDKDSGDVAALRGSRKFNILGTNTKVMNMEESNNGSLSAEFKHLTLREQR
CGNGGRANCDASLIVTEELHLITFETEVYHQGLKIDLETHSLSVVVISNICQMPNAWASILWYNMLTNNPK
NVNFFTKPPIGTWDQVAEVLSWQFSSTTKRGLSIEQLTTLAEKLLGPGVNYSGCQITWANFCKENMAGKG
FSYWVWLDNIIDLVKKYILALWNEGYIMGFISKERERAILSTKPPGTFLLRFSESSKEGGVTFTWVEKDISG
KTQIQSVEPYTKQQLNNMSFAEIIMGYKIMDATNILLSPLVYLYPDIPKEEAFGKYCRPESQEHPEADPGSA
APYLKTKFICVTPTTCSNTIDLPMSPRALDSLMQFGNNGEGAEPSAGGQFESLTFDMELTSECATSPM
>T01542
MALAGAPAGGPCAPALEALLGAGALRLLDSSQIVIISAAQDASAPPAPTGPAAPAAGPCDPDLLLFATPQAP
RPTPSAPRPALGRPPVKRRLDLETDHQYLAESSGPARGRGRHPGKGVKSPGEKSRYETSLNLTTKRFLELL
SHSADGVVDLNWAAEVLKVQKRRIYDITNVLEGIQLIAKKSKNHIQWLGSHTTVGVGGRLEGLTQDLRQ
LQESEQQLDHLMNICTTQLRLLSEDTDSQRLAYVTCQDLRSIADPAEQMVMVIKAPPETQLQAVDSSENFQ
ISLKSKQGPIDVFLCPEETVGGISPGKTPSQEVTSEEENRATDSATIVSPPPSSPPSSLTTDPSQSLLSLEQEPLL
SRMGSLRAPVDEDRLSPLVAADSLLEHVREDFSGLLPEEFISLSPPHEALDYHFGLEEGEGIRDLFDCDFGD
LTPLDF
>T01580
MSLWGLVSKMPPEKVQRLYVDFPQHLRHLLGDWLESQPWEFLVGSDAFCCNMASALLSDTVQHLQASV

GEQGEGSTILQHISTLESIYQRDPLKLVATFRQILQGEKKAVIEQFRHLPMPFHWKQEELKFKTGLRRLQHR
VGEIHLLREALQKGAEAGQVSLHSLIETPANGTGPSEALAMLLQETTGELEAAKALVLKRIQIWKRQQQL
AGNGAPFEESLAPLQERCESLVDIYSQLQQEVGAAGGELEPKTRASLTGRLDEVLRTLVTSCFLVEKQPPQ
VLKTQTKFQAGVRFLLGLRFLGAPAKPPLVRADMVTEKQARELSAPQGPGAGAESTGEIINNTVPLENSIP
GNCCSALFKNLLLKKIKRCERKGTESVTEEKCAVLFSASFTLGPGKLPIQLQALSLPLVVIVHGNQDNNAK
ATILWDNAFSEMDRVPFVVAERVPWEKMCETLNLKFMAEVGTNRGLLPEHFLFLAQKIFNDNSLSMEAFQ
HRSVSWSQFNKEILLGRGFTFWQWFDGVLDLTKRCLRSYWSDRLIIGFISKQYVTSLLLNEPDGTFLLRFS
DSEIGGITIAHVIRGQDGSPQIENIQPFSAKDLSIRSLGDRIRDLAQLKNLYPKKPKDEAFRSHYKPEQMGKD
GRGYVPATIKMTVERDQPLPTPELQMPTMVPSYDLGMAPDSSMSMQLGPDMVPQVYPPHSHSIPPYQGLS
PEESVNVLSAFQEPHLQMPPSLGQMSLPFDQPHPQGLLPCQPQEHAVSSPDPLLCSDVTMVEDSCLSQPVT
AFPQGTWIGEDIFPPLLPPTEQDLTKLLLEGQGESGGGSLGAQPLLQPSHYGQSGISMSHMDLRANPSW
>T01599
MEVNTSASEILYSAPPGDPLSTNYSLAPNTPINQNVSLHQASLGGCSQDFLLFSPEVESLPVASSSTLLPLAP
SNSTSLNSTFGSTNLTGLFFPPQLNGTANDTAGPELPDPLGGLLDEAMLDEISLMDLAIEEGFNPVQASQLE
EEFDSDSGLSLDSSHSPSSLSSSEGSSSSSSSSSSSSSSASSSASSSFSEEGAVGYSSDSETLDLEEAEGAVGYQ
PEYSKFCRMSYQDPAQLSCLPYLEHVGHNHTYNMAPSALDSADLPPPSALKKGSKEKQADFLDKQMSRD
EHRARAMKIPFTNDKIINLPVEEFNELLSKYQLSEAQLSLIRDIRRRGKNKMAAQNCRKRKLDTILNLERD
VEDLQRDKARLLREKVEFLRSLRQMKQKVQSLYQEVFGRLRDENGRPYSPSQYALQYAGDGSVLLIPRT
MADQQARRQERKPKDRRK
>T01795
MNSSSANITYASRKRRKPVQKTVKPIPAEGIKSNPSKRHRDRLNTELDRLASLLPFPQDVINKLDKLSVLRL
SVSYLRAKSFFDVALKSSPTERNGGQDNCRAANFREGLNLQEGEFLLQALNGFVLVVTTDALVFYASSTIQ
DYLGFQQSDVIHQSVYELIHTEDRAEFQRQLHWALNPSQCTESGQGIEEATGLPQTVVCYNPDQIPPENSP
LMERCFICRLRCLLDNSSGFLAMNFQGKLKYLHGQKKKGKDGSILPPQLALFAIATPLQPPSILEIRTKNFIF
RTKHKLDFTPIGCDAKGRIVLGYTEAELCTRGSGYQFIHAADMLYCAESHIRMIKTGESGMIVFRLLTKNN
RWTWVQSNARLLYKNGRPDYIIVTQRPLTDEEGTEHLRKRNTKLPFMFTTGEAVLYEATNPFPAIMDPLPL
RTKNGTSGKDSATTSTLSKDSLNPSSLLAAMMQQDESIYLYPASSTSSTAPFENNFFNESMNECRNWQDNT
APMGNDTILKHEQIDQPQDVNSFAGGHPGLFQDSKNSDLYSIMKNLGIDFEDIRHMQNEKFFRNDFSGEV
DFRDIDLTDEILTYVQDSLSKSPFIPSDYQQQQSLALNSSCMVQEHLHLEQQQQHHQKQVVVEPQQQLCQ
KMKHMQVNGMFENWNSNQFVPFNCPQQDPQQYNVFTDLHGISQEFPYKSEMDSMPYTQNFISCNQPVL
PQHSKCTELDYPMGSFEPSPYPTTSSLEDFVTCLQLPENQKHGLNPQSAIITPQTCYAGAVSMYQCQPEPQ
HTHVGQMQYNPVLPGQQAFLNKFQNGVFK
>T01823
MDMHCKADPFSAMHPGHGGVNQLGGVFVNGRPLPDVVRQRIVELAHQGVRPCDISRQLRVSHGCVSKIL
GRYYETGSIKPGVIGGSKPKVATPKVVDKIAEYKRQNPTMFAWEIRDRLLAEGICDNDTVPSVSSINRIIRTK
VQQPFHPTPDGAGTGVTAPGHTIVPSTASPPVSSASNDPVGSYSINGILGIPRSNGEKRKRDEVEVYTDPAHI
RGGGGLHLVWTLRDVSEGSVPNGDSQSGVDSLRKHLRADTFTQQQLEALDRVFERPSYPDVFQASEHIKS
EQGNEYSLPALTPGLDEVKSSLSASTNPELGSNVSGTQTYPVVTGRDMASTTLPGYPPHVPPTGQGSYPTS
TLAGMVPGSEFSGNPYSHPQYTAYNEAWRFSNPALLSSPYYYSAAPRSAPAARAAAYDRH
>T01973
NSGAPDPGGGCGSRDGRARPGGLSTLCSPTPGPSWSTTAPAPNFTTLPHLSPETPATKKSSRRRSGDSGSPA
PPHRGRPNTVMATQVMGQSSGGGGLFTSSGNIGMALPNDMYDLHDLSKAELAAPQLIMLANVALTGEVN
GSCCDYLVGEERQMAELMPVGDNNFSDSEEGEGLEESADIKGEPHGLENMELRSLELSVVEPQPVFEASG
APDIYSSNKDLPPETPGAEDKGKSSKTKPFRCKPCQYEAESEEQFVHHIRVHSAKKFFVEESAEKQAKARE

SGSSTAEEGDFSKGPIRCDRCGYNTNRYDHYTAHLKHHTRAGDNERVYKCIICTYTTVSEYHWRKHLRNH
FPRKVYTCGKCNYFSDRKNNYVQHVRTHTGERPYKCELCPYSSSQKTHLTRHMRTHSGEKPFKCDQCSY
VASNQHEVTRHARQVHNGPKPLNCPHCDYKTADRSNFKKHVELHVNPRQFNCPVCDYAASKKCNLQYH
FKSKHPTCPNKTMDVSKVKLKKTKKREADLPDNITNEKTEIEQTKIKGDVAGKKNEKSVKAEKRDVSKE
KKPSNNVSVIQVTTRTRKSVTEVKEMDVHTGSNSEKFSKTKKSKRKLEVDSHSLHGPVNDEESSTKKKKK
VESKSKNNSQEVPKGDSKVEENKKQNTCMKKSTKKKTLKNKSSKKSSKPSRNS
>T02068
MEGFPLVPPPSEDLVPYDTDLYQRQTHEYYPYLSSDGESHSDHYWDFHPHHVHSEFESFAENNFTELQSV
QPPQLQQLYRHMELEQMHVLDTPMVPPHPSLGHQVSYLPRMCLQYPSLSPAQPSSDEEEGERQSPPLEVS
DGEADGLEPGPGLLPGETGSKKKIRLYQFLLDLLRSGDMKDSIWWVDKDKGTFQFSSKHKEALAHRWGI
QKGNRKKMTYQKMARALRNYGKTGEVKKVKKKLTYQFSGEVLGRGGLAERRHPPH
>T02256
MRIPVDASTSRRFTPPSTALSPGKMSEALPLGAPDAGAALAGKLRSGDRSMVEVLADHPGELVRTDSPNF
LCSVLPTHWRCNKTLPIAFKVVALGDVPDGTLVTVMAGNDENYSAELRNATAAMKNQVARFNDLRFVG
RSGRGKSFTLTITVFTNPPQVATYHRAIKITVDGPREPRRHRQKLDDQTKPGSLSFSERLSELEQLRRTAMR
VSPHHPAPTPNPRASLNHSTAFNPQPQSQMQEEDTAPWRC
>T02336
MDLTKMGMIQLQNPSHPTGLLCKANQMRLAGTLCDVVIMVDSQEFHAHRTVLACTSKMFEILFHRNSQH
YTLDFLSPKTFQQILEYAYTATLQAKAEDLDDLLYAAEILEIEYLEEQCLKMLETIQASDDNDTEATMADG
GAEEEEDRKARYLKNIFISKHSSEESGYASVAGQSLPGPMVDQSPSVSTSFGLSAMSPTKAAVDSLMTIGQS
LLQGTLQPPAGPEEPTLAGGGRHPGVAEVKTEMMQVDEVPSQDSPGAAESSISGGMGDKVEERGKEGPG
TPTRSSVITSARELHYGREESAEQVPPPAEAGQAPTGRPEHPAPPPEKHLGIYSVLPNHKADAVLSMPSSVT
SGLHVQPALAVSMDFSTYGGLLPQGFIQRELFSKLGELAVGMKSESRTIGEQCSVCGVELPDNEAVEQHRK
LHSGMKTYGCELCGKRFLDSLRLRMHLLAHSAGAKAFVCDQCGAQFSKEDALETHRQTHTGTDMAVFC
LLCGKRFQAQSALQQHMEVHAGVRSYICSECNRTFPSHTALKRHLRSHTGDHPYECEFCGSCFRDESTLK
SHKRIHTGEKPYECNGCDKKFSLKHQLETHYRVHTGEKPFECKLCHQRSRDYSAMIKHLRTHNGASPYQC
TICTEYCPSLSSMQKHMKGHKPEEIPPDWRIEKTYLYLCYV
>T02338
MTAPEKPVKQEEMAALDVDSGGGGGGGGHGEYLQQQQHGNGAVAAAAAAQDTQPSPLALLAATCS
KIGPPSPGDDEEEAAAAAGAPAAAGATGDLASAQLGGAPNRWEVLSATPTTIKDEAGNLVQIPSAATSSGQ
YVLPLQNLQNQQIFSVAPGSDSSNGAVSSVQYQVIPQIQSADGQQVQIGFTGSSDNGGINQESSQIQIIPGSN
QTLLASGTPSANIQNLIPQTGQVQVQGVAIGGSSFPGQTQVVANVPLGLPGNITFVPINSVDLDSLGLSGSS
QTMTAGINADGHLINTGQAMDSSDNSERTGERVSPDINETNTDTDLFVPTSSSSQLPVTIDSTGILQQNTNS
LTTSSGQVHSSDLQGNYIQSPVSEETQAQNIQVSTAQPVVQHLQLQESQQPTSQAQIVQGITPQTIHGVQAS
GQNISQQALQNLQLQLNPGTFLIQAQTVTPSGQVTWQTFQVQGVQNLQNLQIQNTAAQQITLTPVQTLTL
GQVAAGGAFTSTPVSLSTGQLPNLQTVTVNSIDSAGIQLHPGENADSPADIRIKEEEPDPEEWQLSGDSTLN
TNDLTHLRVQVVDEEGDQQHQEGKRLRRVACTCPNCKEGGGRGTNLGKKKQHICHIPGCGKVYGKTSHL
RAHLRWHSGERPFVCNWMYCGKRFTRSDELQRHRRTHTGEKKFVCPECSKRFMRSDHLAKHIKTHQNK
KGIHSSSTVLASVEAARDDTLITAGGTTLILAKIQQGSVSGIGTVNTSATSNQDILTNTEIPLQLVTVSGNET
ME
>T02758
MDMADYSAALDPAYTTLEFENVQVLTMGNDTSPSEGTNLNAPNSLGVSALCAICGDRATGKHYGASSCD
GCKGFFRRSVRKNHMYSCRFSRQCVVDKDKRNQCRYCRLKKCFRAGMKKEAVQNERDRISTRRSSYED
SSLPSINALLQAEVLSRQITSPVSGINGDIRAKKIASIADVCESMKEQLLVLVEWAKYIPAFCELPLDDQVAL

LRAHAGEHLLLGATKRSMVFKDVLLLGNDYIVPRHCPELAEMSRVSIRILDELVLPFQELQIDDNEYAYLK
AIIFFDPDAKGLSDPGKIKRLRSQVQVSLEDYINDRQYDSRGRFGELLLLLPTLQSITWQMIEQIQFIKLFGM
AKIDNLLQEMLLGGSPSDAPHAHHPLHPHLMQEHMGTNVIVANTMPTHLSNGQMCEWPRPRGQAATPET
PQPSPPGASGSEPYKLLPGAVATIVKPLSAIPQPTITKQEVI

>T02905

MPQLSGGGGGGGGDPELCATDEMIPFKDEGDPQKEKIFAEISHPEEEGDLADIKSSLVNESEIIPASNGHEVA
RQAQTSQEPYHDKAREHPDDGKHPDGGLYNKGPSYSSYSGYIMMPNMNNDPYMSNGSLSPPIPRTSNKV
PVVQPSHAVHPLTPLITYSDEHFSPGSHPSHIPSDVNSKQGMSRHPPAPEIPTFYPLSPGGVGQITPPLGWQG
QPVYPITGGFRQPYPSSLSVDTSMSRFSHHMIPGPPGPHTTGIPHPAIVTPQVKQEHPHTDSDLMHVKPQHE
QRKEQEPKRPHIKKPLNAFMLYMKEMRANVVAECTLKESAAINQILGRRWHALSREEQAKYYELARKER
QLHMQLYPGWSARDNYGKKKKRKREKLQESASGTGPRMTAAYI

>T03828

MRLSKTLVDMDMADYSAALDPAYTTLEFENVQVLTMGNDLLPLRLARLRHPLRHHWSISGGVDSSPQGD
TSPSEGTNLNAPNSLGVSALCAICGDRATGKHYGASSCDGCKGFFRRSVRKNHMYSCRFSRQCVVDKDK
RNQCRYCRLKKCFRAGMKKEAVQNERDRISTRRSSYEDSSLPSINALLQAEVLSRQITSPVSGINGDIRAKK
IASIADVCESMKEQLLVLVEWAKYIPAFCELPLDDQVALLRAHAGEHLLLGATKRSMVFKDVLLLGNDYI
VPRHCPELAEMSRVSIRILDELVLPFQELQIDDNEYAYLKAIIFFDPDAKGLSDPGKIKRLRSQVQVSLEDYI
NDRQYDSRGRFGELLLLLPTLQSITWQMIEQIQFIKLFGMAKIDNLLQEMLLGGSPSDAPHAHHPLHPHLM
QEHMGTNVIVANTMPTHLSNGQMCEWPRPRGQAATPETPQPSPPGGSGSEPYKLLPGAVATIVKPLSAIPQ
PTITKQEVI

>T04096

MSSILPFTPPIVKRLLGWKKGEQNGQEEKWCEKAVKSLVKKLKKTGQLDELEKAITTQNVNTKCITIPRSL
DGRLQVSHRKGLPHVIYCRLWRWPDLHSHHELRAMELCEFAFNMKKDEVCVNPYHYQRVETPVLPPVLV
PRHTEIPAEFPPLDDYSHSIPENTNFPAGIEPQSNIPEVGTWAAQAGLTPPPGYLSEDGETSDHQMNHSMDA
GSPNLSPNPMSPAHNNLDLQPVTYCEPAFWCSISYYELNQRVGETFHASQPSMTVDGFTDPSNSERFCLGL
LSNVNRNAAVELTRRHIGRGVRLYYIGGEVFAECLSDSAIFVQSPNCNQRYGWHPATVCKIPPGCNLKIFNN
QEFAALLAQSVNQGFEAVYQLTRMCTIRMSFVKGWGAEYRRQTVTSTPCWIELHLNGPLQWLDKVLTQ
MGSPSIRCSSVS

>T04169

MASDLESSLTSIDWLPQLTLRATIEKLGSASQAGPPGSSRKCSPGSPTDPNATLSKDEAAVHQDGKPRYSYA
TLITYAINSSPAKKMTLSEIYRWICDNFPYYKNAGIGWKNSIRHNLSLNKCFRKVPRPRDDPGKGSYWTID
TCPDISRKRRHPPDDDLSQDSPEQEASKSPRGGVAGSGEASLPPEGNPQMSLQSPTSIASYSQGTGSVDGGA
VAAGASGRESAEGPPPLYNTNHDFKFSYSEINFQDLSWSFRNLYKSMLEKSSSSSQHGFSSLLGDIPPSNNY
YMYQQQQPPPPQQQQQQQQPPQPPPQQSQPQQQQAPAQGPSAVGGAPPLHTPSTDGCTPPGGKQAGAEG
YGPPPVMAMHPPPLQHGGYHPHQHHPHSHPAQQPPPPQPQAQGQAPINNTGFAFPSDWCSNIDSLKESFK
MVNRLNWSSIEQSQFSELMESLRQAEQKNWTLDQHHIANLCDSLNHFLTQTGHVPPQGGTHRPPAPARIA
DSCALTSGKQESAMSQVNSYGHPQAPHLYPGPSPMYPIPTQDSAGYNRPAHHMVPRPSVPPPGANEEIPDD
FDWDLIT

>T04292

MDNMSITNTPTSNDACLSIVHSLMCHRQGGESETFAKRAIESLVKKLKEKKDELDSLITAITTNGAHPSKC
VTIQRTLDGRLQVAGRKGFPHVIYARLWRWPDLHKNELKHVKYCQYAFDLKCDSVCVNPYHYERVVSPG
IDLSGLTLQSNAPSSMMVKDEYVHDFEGQPSLSTEGHSIQTIQHPPSNRASTETYSTPALLAPSESNATSTAN
FPNIPVASTSQPASILGGSHSEGLLQIASGPQPGQQQNGFTGQPATYHHNSTTTWTGSRTAPYTPNLPHHQN
GHLQHHPPMPPHPGHYWPVHNELAFQPPISNHPAPEYWCSIAYFEMDVQVGETFKVPSSCPIVTVDGYVD

PSGGDRFCLGQLSNVHRTEAIERARLHIGKGVQLECKGEGDVWVRCLSDHAVFVQSYYLDREAGRAPGD
AVHKIYPSAYIKVFDLRQCHRQMQQQAATAQAAAAAQAAAVAGNIPGPGSVGGIAPAISLSAAAGIGVDD
LRRLCILRMSFVKGWGPDYPRQSIKETPCWIEIHLHRALQLLDEVLHTMPIADPQPLD
>T04323
MFPSPALTPTPFSVKDILNLEQQQRSLAAAGELSARLEATLAPSSCMLAAFKPEAYAGPEAAAPGLPELRAE
LGRAPSPAKCASAFPAAPAFYPRAYSDPDPAKDPRAEKKELCALQKAVELEKTEADNAERPRARRRRKPR
VLFSQAQVYELERRFKQQRYLSAPERDQLASVLKLTSTQVKIWFQNRRYKCKRQRQDQTLELVGLPPPPP
PPARRIAVPVLVRDGKPCLGDSAPYAPAYGVGLNPYGYNAYPAYPGYGGAACSPGYSCTAAYPAGPSPAQP
ATAAANNNFVNFGVGDLNAVQSPGIPQSNSGVSTLHGIRAW
>T04362
MNGEEQYYAATQLYKDPCAFQRGPAPEFSASPPACLYMGRQPPPPPPHPFPGALGALEQGSPPDISPYEVPP
LADDPAVAHLHHHLPAQLALPHPPAGPFPEGAEPGVLEEPNRVQLPFPWMKSTKAHAWKGQWAGGAYAA
EPEENKRTRTAYTRAQLLELEKEFLFNKYISRPRRVELAVMLNLTERHIKIWFQNRRMKWKKEEDKKRGG
GTAVGGGGVAEPEQDCAVTSGEELLALPPPPPPGGAVPPAAPVAAREGRLPPGLSASPQPSSVAPRRPQEPR
>T04673
MGTPKPRILPWLVSQLDLGQLEGVAWVNKSRTRFRIPWKHGLRQDAQQEDFGIFQAWAEATGAYVPGRD
KPDLPTWKRNFRSALNRKEGLRLAEDRSKDPHDPHKIYEFVNSGVGDFSQPDTSPDTNGGGSTSDTQEDI
LDELLGNMVLAPLPDPGPPSLAVAPEPCPQPLRSPSLDNPTPFPNLGPSENPLKRLLVPGEEWEFEVTAFYR
GRQVFQQTISCPEGLRLVGSEVGDRTLPGWPVTLPDPGMSLTDRGVMSYVRHVLSCLGGGLALWRAGQ
WLWAQRLGHCHTYWAVSEELLPNSGHGPDGEVPKDKEGGVFDLGPFIVDLITFTEGSGRSPRYALWFCVG
ESWPQDQPWTKRLVMVKVVPTCLRALVEMARVGGASSLENTVDLHISNSHPLSLTSDQYKAYLQDLVEG
MDFQGPGES
>T04674
MALAPERAAPRVLFGEWLLGEISSGCYEGLQWLDEARTCFRVPWKHFARKDLSEADARIFKAWAVARGR
WPPSSRGGGPPPEAETAERAGWKTNFRCALRSTRRFVMLRDNSGDPADPHKVYALSRELCWREGPGTDQ
TEAEAPAAVPPPQGGPPGPFLAHTHAGLQAPGPLPAPAGDKGDLLLQAVQQSCLADHLLTASWGADPVPT
KAPGEGQEGLPLTGACAGGPGLPAGELYGWAVETTPSPGPQPAALTTGEAAAPESPHQAEPYLSPSPSACTA
VQEPSPGALDVTIMYKGRTVLQKVVGHPSCTFLYGPPDPAVRATDPQQVAFPSPAELPDQKQLRYTEELLR
HVAPGLHLELRGPQLWARRMGKCKVYWEVGGPPGSASPSTPACLLPRNCDTPIFDFRVFFQELVEFRARQ
RRGSPRYTIYLGFGQDLSAGRPKEKSLVLVKLEPWLCRVHLEGTQREGVSSLDSSSLSLCLSSANSLYDDIE
CFLMELEQPA
>T04675
KVSNEEKPKVAIGEECRADEQAFLVALYKYMKERKTPIERIPYLGFKQINLWTMFQAAQKLGGYETITARR
QWKHIYDELGGNPGSTSAATCTRRHYERLILPYERFIKGEEDKPLPPIKPRKQENSSQENENKTKVSGTKRI
KHEIPKSKKEKENAPKPQDAAEVSSEQEKEQETLISQKSIPEPLPAADMKKKIEGYQEFSAKPLASRVDPEK
DNETDQGSNSEKVAEEAGEKGPTPPLPSAPL
>T05990
MATDAALRRLLRLHRTEIAVAVDSAFPLLHALADHDVVPEDKFQETLHLKEKEGCPQAFHALLSWLLTQD
STAILDFWRVLFKDYNLERYGRLQPILDSFPKDVDLSQPRKGRKPPAVPKALVPPPRLPTKRKASEEARAA
APAALTPRGTASPGSQLKAKPPKKPESSAEQQRLPLGNGIQTMSASVQRAVAMSSGDVPGARGAVEGILIQ
QVFESGGSKKCIQVGGEFYTPSKFEDSGSGKNKARSSSGPKPLVRAKGAQGAAPGGGEARLGQQGSVPAP
LALPSDPQLHQKNEDECAVCRDGGELICCDGCPRAFHLACLSPPLREIPSGTWRCSSCLQATVQEVQPRAE
EPRPQEPPVETPLPPGLRSAGEEVRGPPGEPLAGMDTTLVYKHLPAPPSAAPLPGLDSSALHPLLCVGPEGQ
QNLAPGARCGVCGDGTDVLRCTHCAAAFHWRCHFPAGTSRPGTGLRCRSCSGDVTPAPVEGVLAPSPAR

LAPGPAKDDTASHEPALHRDDLESLLSEHTFDGILQWAIQSMARPAAPFPS

>T00017

MHRLLAWDAACLPPPPAAFRPMEVANFYYEPDCLAYGAKAARAAPRAPAAEPAIGEHERAIDFSPYLEPL

APAADFAAPAPAHHDFLSDLFADDYGAKPSKKPADYGYVSLGRAGAKAAPPACFPPPPPAALKAEPGFEPA

DCKRADDAPAMAAGFPFALRAYLGYQATPSGSSGSLSTSSSSSPPGTPSPADAKAAPAACFAGPPAAPAKA

KAKKTVDKLSDEYKMRRERNNIAVRKSRDKAKMRNLETQHKVLELTAENERLQKKVEQLSRELSTLRNL

FKQLPEPLLASAGHC

>T00018

MSSGANITYASRKRRKPVQKTVKPIPAEGIKSNPSKRHRDRLNTELDRLASLLPFPQDVINKLDKLSVLRLS

VSYLRAKSFFDVALKSTPADRNGGQDQCRAQIRDWQDLQEGEFLLQALNGFVLVVTADAFLFYASSTIQD

YLGFQQSDVIHQSVYELIHTEDRAEFQRHVHWALNPDSAQGVDEAHGPPQAAVYYTPDQLPPENASFME

RCFRCRLRCLLDNSSGFLAMNFQGRLKYLHGQNKKGKDGALLPPQLALFAIATPLQPPSILEIRTKNFIFRT

KHKLDFTPIGCDAKGQLILGYTEVELCTRGSGYQFIHAADILHCAESHIRMIKTGESGMTVFRLLAKHSRW

RWVQSNARLIYRNGRPDYIIATQRPLTDEEGREHLQKRSTSLPFMFATGEAVLYEISSPFSPIMDPLPIRTKSN

TSRKDWAPQSTPSKDSFHPSSLMSALIQQDESIYLCPPSSPALLDSHFLMGSVSKCGSWQDSFAAAGSEAA

LKHEQIGHAQDVNLALSGGPSELFPDNKNNDLYSIMRNLGIDFEDIRSMQNEEFFRTDSTAAGEVDFKDIDI

TDEILTYVQDSLNNSTLLNSACQQQPVTQHLSCMLQERLQLEQQQQLQQPPPQALEPQQQLCQMVCPQQ

DLGPKHTQINGTFASWNPTPPVSFNCPQQELKHYQLFSSLQGTAQEFPYKPEVDSVPYTQNFAPCNQPLLP

EHSKSVQLDFPGRDFEPSLHPTTSNLDFVSCLQVPENQSHGINSQSAMVSPQAYYAGAMSMYQCQPGPQR

TPVDQTQYSSEIPGSQAFLSKVQS

>T00111

MKAAVDLKPTLTIIKTEKVDLELFPSPDMECADVPLLTPSSKEMMSQALKATFSGFTKEQQRLGIPKDPRQ

WTETHVRDWVMWAVNEFSLKGVDFQKFCMSGAALCALGKECFLELAPDFVGDILWEHLEILQKEDVKP

YQVNGANPTYPESCYTSDYFISYGIEHAQCVPPSEFSEPSFITESYQTLHPISSEELLSLKYENDYPSVILQDP

LQTDTLQTDYFAIKQEVLTPDNMCLGRASRGKLGGQDSFESVESYDSCDRLTQSWSSQSSFNSLQRVPSYD

SFDYEDYPAALPNHKPKGTFKDYVRDRADLNKDKPVIPAAALAGYTGSGPIQLWQFLLELLTDKSCQSFIS

WTGDGWEFKLSDPDEVARRWGKRKNKPKMNYEKLSRGLRYYYDKNIIHKTAGKRYVYRFVCDLQSLLG

YTPEELHAMLDVKPDAD

>T00138

MARRPRHSIYSSDEDDEDIEMCDHDYDGLLPKSGKRHLGKTRWTREEDEKLKKLVEQNGTDDWKVIAN

YLPNRTDVQCQHRWQKVLNPELIKGPWTKEEDQRVIELVQKYGPKRWSVIAKHLKGRIGKQCRERWHNH

LNPEVKKTSWTEEEDRIIYQAHKRLGNRWAEIAKLLPGRTDNAIKNHWNSTMRRKVEQEGYLQEPSKAS

QTPVATSFQKNNHLMGFGHASPPSQLSPSGQSSVNSEYPYYHIAEAQNISSHVPYPVALHVNIVNVPQPAA

AAIQRHYNDEDPEKEKRIKELELLLMSTENELKGQQALPTQNHTCSYPGWHSTSIVDQTRPHGDSAPVSC

LGEHHATPSLPADPGSLPEESASPARCMIVHQGTILDNVKNLLEFAETLQFIDSFLNTSSNHESSGLDAPTLP

STPLIGHKLTPCRDQTVKTQKENSIFRTPAIKRSILESSPRTPTPFKHALAAQEIKYGPLKMLPQTPSHAVEDL

QDVIKRESDESGIVAEFQESGPPLLKKIKQEVESPTEKSGNFFCSNHWAENSLSTQLFSQASPVADAPNILTS

SVLMTPVSEDEDNVLKAFTVPKNRPLVGPLQPCSGAWEPASCGKTEDQMTASGPARKYVNAFSARTLVM

>T00244

MAAAKAEMQLMSPLQISDPFGSFPHSPTMDNYPKLEEMMLLSNGAPQFLGAAGTPEGSGGNSSSSTSSGG

GGGGGSNSGSSAFNPQGEPSEQPYEHLTTESFSDIALNNEKAMVETSYPSQTTRLPPITYTGRFSLEPAPNS

GNTLWPEPLFSLVSGLVSMTNPPTSSSSAPSPAASSSSSASQSPPLSCAVPSNDSSPIYSAAPTFPTPNTDIFPE

PQSQAFPGSAGTALQYPPPAYPATKGGFQVPMIPDYLFPQQQGDLSLGTPDQKPFQGLENRTQQPSLTPLST

IKAFATQSGSQDLKALNTTYQSQLIKPSRMRKYPNRPSKTPPHERPYACPVESCDRRFSRSDELTRHIRIHTG

QKPFQCRICMRNFSRSDHLTTHIRTHTGEKPFACDICGRKFARSDERKRHTKIHLRQKDKKADKSVVASPA
ASSLSSYPSPVATSYPSPATTSFPSPVPTSYSSPGSSTYPSPAHSGFPSPSVATTFASVPPAFPTQVSSFPSAGVS
SSFSTSTGLSDMTATFSPRTIEIC
>T00278
MASGDTLYIATDGSEMPAEIVELHEIEVETIPVETIETTVVGEEEEEDDDDEDGGGGDHGGGGGGHGHAG
HHHHHHHHHHHHPPMIALQPLVTDDPTQVHHHQEVILVQTREEVVGGDDSDGLRAEDGFEDQILIPVPAP
AGGDDDYIEQTLVTVAAAGKSGGGASSGGGRVKKGGGKKSGKKSYLGGGAGAAGGGGADPGNKKWE
QKQVQIKTLEGEFSVTMWSSDEKKDIDHETVVEEQIIGENSPPDYSEYMTGKKLPPGGIPGIDLSDPKQLA
EFARMKPRKIKEDDAPRTIACPHKGCTKMFRDNSAMRKHLHTHGPRVHVCAECGKAFVESSKLKRHQLV
HTGEKPFQCTFEGCGKRFSLDFNLRTHVRIHTGDRPYVCPFDGCNKKFAQSTNLKSHILTHAKAKNNQ
>T00377
MSSYFVNSFCGRYPNGPDYQLHNYGDHSSVSEQFRDSASMHSGRYGYGYNGMDLSVGRSGSGHFGSGE
RARSYAAGASAAPAEPRYSQPATSTHSPPPDPLPCSAVAPSPGSDSHHGGKNSLGNSSGASANAGSTHISSR
EGVGTASAAEEDAPASSEQAGAQSEPSPAPPAQPQIYPWMRKLHISHDNIGGPEGKRARTAYTRYQTLELE
KEFHFNRYLTRRRRIEIAHALCLSERQIKIWFQNRRMKWKKDNKLKSMSMAAAGGAFRP
>T00402
MCDRNGGRRLRQWLIEQIDSSMYPGLIWENDEKTMFRIPWKHAGKQDYNQEVDASIFKAWAVFKGKFKE
GDKAEPATWKTRLRCALNKSPDFEEVTDRSQLDISEPYKVYRIVPEEEQKCKLGVAPAGCMSEVPEMECG
RSEIEELIKEPSVDEYMGMTKRSPSPPEACRSQILPDWWVQQPSAGLPLVTGYAAYDTHHSAFSQMVISFY
YGGKLVGQATTTCLEGCRLSLSQPGLPKLYGPDGLEPVCFPTADTIPSERQRQVTRKLFGHLERGVLLHSN
RKGVFVKRLCQGRVFCSGNAVVCKGRPNKLERDEVVQVFDTNQFIRELQQFYATQSRLPDSRVVLCFGEE
FPDTVPLRSKLILVQVEQLYARQLVEEAGKSCGAGSLMPALEEPQPDQAFRMFPDICTSHQRPFFRENQQIT
V
>T00425
MPVERMRMRPWLEEQINSNTIPGLKWLNKEKKIFQIPWMHAARHGWDVEKDAPLFRNWAIHTGKHQPG
IDKPDPKTWKANFRCAMNSLPDIEEVKDRSIKKGNNAFRVYRMLPLSERPSKKGKKPKTEKEERVKHIKQ
EPVESSLGLSNGVSGFSPEYAVLTSAIKNEVDSTVNIIVVGQSHLDSNIEDQEIVTNPPDICQVVEVTTESDD
QPVSMSELYPLQISPVSSYAESETTDSVASDEENAEGRPHWRKRSIEGKQYLSNMGTRNTYLLPSMATFVT
SNKPDLQVTIKEDSCPMPYNSSWPPFTDLPLPAPVTPTPSSSRPDRETRASVIKKTSDITQARVKSC
>T00454
MNGVAGDGMINIDMTGEKRPLDLPYPSSFAPISAPRNQTFTYMGKFSIDPQYPGASCYPEGIINIVSAGILQ
GVTPPASTTASSSVTSASPNPLATGPLGVCTMSQTQPELDHLYSPPPPPPPYSGCTGDLYQDPSAFLSPPSTTS
TSSLAYQPPPSYPSPKPAMDPGLIPMIPDYPGFFPSPCQRDPHGAAGPDRKPFPCPLDSLRVPPPLTPLSTIRN
FTLGGPGAGVTGPGASGGGEGPRLPGSGSAAVTATPYNPHHLPLRPILRPRKYPNRPSKTPVHERPYPCPAE
GCDRRFSRSDELTRHIRIHTGHKPFQCRICMRNFSRSDHLTTHIRTHTGEKPFACDYCGRKFARSDERKRHT
KIHLRQKERKSSAPSAPPSAQSSASGPGGSQAGGSLCGNSAIGGPLASCTSRTRTP
>T00505
MGRKKIQITRIMDERNRQVTFTKRKFGLMKKAYELSVLCDCEIALIIFNSSNKLFQYASTDMDKVLLKYTE
YNEPHESRTNSDIVETLRKKGLNGCENPDADDYFEHSPLSEDRFIKLNEDSDFIFKRGPPGFPPQNFSMSVT
VPVTSPNPLSDTNPGSSLVSPSLAASSTLAETSMLSPPPATLHRNVSPGAPQRPPSTGSASGMLSTTDLTVPN
GAGNSPVGNGFVNSRASPNLIGNTGANSLGKVMPTKSPPPPGGGSLGMNSRKPDLRVAIPPSSKGMMPPL
SEEEELELNAQRISSSQATQPLATPVVSVTTPSLPPQGLVYSAMPTAYNTDYSLTSADLSALQGFTSPGMLS
LGQASAWQEHHLGQTTLSSLVAGGQLSQGSNLSINTNQNINIKSEPISPPRDRMTPSGFQHHHHHPQQQPPP
QPPQPQPRQEMGRSPVDSLSSSSSSYDGSDREDPRGDFHSPIVLGRPANTEDRESPSVKRMRMDTWVT

>T00681
MQNSHSGVNQLGGVFVNGRPLPDSTRQKIVELAHSGARPCDISRILQVSNGCVSKILGRYYETGSIRPRAIG
GSKPRVATPEVVSKIAQYKRECPSIFAWEIRDRLLSEGVCTNDNIPSVSSINRVLRNLASEKQQMGADGMY
DKLRMLNGQTGSWGTRPGWYPGTSVPGQPTQDGCQQQEGGGENTNSISSNGEDSDEAQMRLQLKRKLQ
RNRTSFTQEQIEALEKEFERTHYPDVFARERLAAKIDLPEARIQVWFSNRRAKWRREEKLRNQRRQASNTP
SHIPISSSFSTSVYQPIPQPTTPVSSFTSGSMLGRTDTALTNTYSALPPMPSFTMANNLPMQPPVPSQTSSYSC
MLPTSPSVNGRSYDTYTPPHMQTHMNSQPMGTSGTTSTGLISPGVSVPVQVPGSEPDMSQYWPRLQ
>T00694
MVDTESPICPLSPLEADDLESPLSEEFLQEMGNIQEISQSIGEESSGSFGFADYQYLGSCPGSEGSVITDTLSP
RSSPSSVSCPVIPASTDESPGSALNIECRICGDKASGYHYGVHACEGCKGFFRRTIRLKLVYDKCDRSCKIQ
KKNRNKCQYCRFHKCLSVGMSHNAIRFGRMPRSEKAKLKAEILTCEHDLKDSETADLKSLGKRIHEAYLK
NFNMNKVKARVILAGKTSNNPPFVIHDMETLCMAEKTLVAKMVANGVEDKEAEVRFFHCCQCMSVETV
TELTEFAKAIPGFANLDLNDQVTLLKYGVYEAIFTMLSSLMNKDGMLIAYGNGFITREFLKNLRKPFCDIM
EPKFDFAMKFNALELDDSDISLFVAAIICCGDRPGLLNIGYIEKLQEGIVHVLKLHLQSNHPDDTFLFPKLLQ
KMVDLRQLVTEHAQLVQVIKKTESDAALHPLLQEIYRDMY
>T00702
MEGFSLTAPPSDDLVTYDSELYQRPMHDYYSFVGSDGESHSDHYWDFSAHHVHNNEFENFPENHFTELQS
VQPPQLQQLYRHMELEQMHVLDTPMVPPHTGLSHQVSYMPRMCFPYQTLSPAHQQSSDEEEGERQSPPL
EVSDGEADGLEPGPGLLHGETGSKKKIRLYQFLLDLLRSGDMKDSIWWVDKDKGTFQFSSKHKEALAHR
WGIQKGNRKKMTYQKMARALRNYGKTGEVKKVKKKLTYQFSGEVLGRGGLAERRLPPH
>T00752
MSDQDHSMDEVTAVVKIEKDVGGNNGGSGNGGGAAFSQTRSSSTGSSSSSGGGGGQESQPSPLALLAATC
SRIESPNENSNNSQGPSQSGGTGELDLTATQLSQGANGWQIISSSSGATPTSKEQSGNSTNGSNGSESSKNRT
VSGGQYVVAATPNLQNQQVLTGLPGVMPNIQYQVIPQFQTVDGQQLQFAATGAQVQQDGSGQIQIIPGAN
QQIIPNEGSGGNIIAAMPNLLQQAVPLQGLANNVLSGQTQYVTNVPVALNGNITLLPVNSVSAATLTPSSQA
GTISSSGSQESSSQPVTSGTAISSASLVSSQASSSSFFTNANSYSTTTTTSNMGIMNFTSSGSSGTSSQGQTPQ
RVGGLQGSDSLNIQQNQTSGGSLQGSQQKEGEQSQQTQQQQILIQPQLVQGGQALQALQAAPLSGQTFTT
QAISQETLQNLQLQAVQNSGPIIIRTPTVGPNGQVSWQTLQLQNLQVQNPQAQTITLAPMQGVSLGQTSSS
NTTLTPIASAASIPAGTVTVNAAQLSSMPGLQTINLSALGTSGIQVHQLPGLPLAIANTPGDHGTQLGLHGS
GGDGIHDETAGGEGENSSDLQPQAGRRTRREACTCPYCKDSEGRASGDPGKKKQHICHIQGCGKVYGKT
SHLRAHLRWHTGERPFMCNWSYCGKRFTRSDELQRHKRTHTGEKKFACPECPKRFMRSDHLSKHIKTHQ
NKKGGPGVALSVGTLPLDSGAGSEGTATPSALITTNMVAMEAICPEGIARLANSGINVMQVTELQSINISGN
GF
>T00877
MKGQQKTAETEEGTVQIQEGAVATGEDPTSVAIASIQSAATFPDPNVKYVFRTENGGQVMYRVIQVSEGQL
DGQTEGSGAISGYPATQSMTQAVIQGAFTSDDAVDTEGAAAETHYTYFPSTAVGDGSGGTTSGSTTAVVTT
QGSEALLGQATPPSTGQFFVMMSPQEVLQGGSQRSIAPRTHPYSPKSEAPRTTRDEKRRAQHNEVERRRR
DKINNWIVQLSKIIPDCSMESTKSGQSKGGILSKACDYIQELRQSNHRLSEELQGLDQLQLDNDVLRQQVE
DLKNKNLLLRAQLRHHGLEVVIKNDSN
>T00989
MTMESGADNQQSGDAAVTEAENQQMTVQAQPQIATLAQVSMPAAHATSSAPTVTLVQLPNGQTVQVHG
VIQAAQPSVIQSPQVQTVQSSCKDLKRLFSGTQISTIAESEDSQESVDSVTDSQKRREILSRRPSYRKILNDL
SSDAPGVPRIEEEKSEEETSAPAITTVTVPTPIYQTSSGQYIAITQGGAIQLANNGTDGVQGLQTLTMTNAA
ATQPGTTILQYAQTTDGQQILVPSNQVVVQAASGDVQTYQIRTAPTSTIAPGVVMASSPALPTQPAEEAAR

KREVRLMKNREAARECRRKKKEYVKCLENRVAVLENQNKTLIEELKALKDLYCHKSD
>T01147
MEMHRIRGSRIGRGRGGEEAALERGGWLSCSAGTWPTNPRTRPGLGTAPCAQTRAIPSQSPSARADPILLP
QADAAGMDYSYDEDLDELCPVCGDKVSGYHYGLLTCESCKGFFKRTVQNNKHYTCTESQSCKIDKTQR
KRCPFCRFQKCLTVGMRLEAVRADRMRGGRNKFGPMYKRDRALKQQKKAQIRANGFKLETGPPMGVPP
PPPPPPDYMLPPSLHAPEPKALVSGPPSGPLGDIGAPSLPMSVPGPHGPLAGYLYPAFSNRTIKSEYPEPYASP
PQQPGPPYSYPEPFSGGPNVPELILQLLQLEPEEDQVRARIVGCLQEPAKSGSDQPAPFSLLCRMADQTFISI
VDWARRCMVFKELEVADQMTLLQNCWSELLVLDHIYRQVQYGKEDSILLVSGQEVTELVKPLVLHNPRP
LRADSGHPKFQIQGHALARLLCVLGPFEEPQCGMVSGSSYRR
>T01201
MDLEKNYPTPRTIRTGHGGVNQLGGVFVNGRPLPDVVRQRIVELAHQGVRPCDISRQLRVSHGCVSKILG
RYYETGSIKPGVIGGSKPKVATPKVVEKIAEYKRQNPTMFAWEIRDRLLAERVCDNDTVPSVSSINRIIRTK
VQQPPNQPVPASSHSIVSTGSVTQVSSVSTDSAGSSYSISGILGITSPSADTNKRKRDEGIQESPVPNGHSLPG
RDFLRKQMRGDLFTQQQLEVLDRVFERQHYSDIFTTTEPIKPEQTTEYSAMASLAGGLDDMKANLTSPTP
ADIGSSVPGPQSYPIVTGRDLASTTLPGYPPHVPPAGQGSYSAPTLTGMVPGSEFSGSPYSHPQYSSYNDSW
RFPNPGLLGSPYYYSPAARGAAPPAAATAYDRH
>T01211
MVSKLSQLQTELLAALLESGLSKEALIQALGEPGPYLMVGEGPLDKGESCGGSRGDLTELPNGLGETRGS
EDDTDDDGEDFAPPILKEPENLSPEEAAHQKAVVESLLQEDPWRVAKMVKSYLQQHNIPQREVVDTTGLN
QSHLSQHLNKGTPMKTQKRAALYTWYVRKQREVAQQFTHAGQGGLIEEPTGDELPTKKGRRNRFKWGP
ASQQILFQAYERQKNPSKEERETLVEECNRAECIQRGVSPSQAQGLGSNLVTEVRVYNWFANRRKEEAFR
HKLAMDTYNGPPPGPGPGPALPAHSSPGLPTTTLSPSKVHGVRYGQSATSEAAEVPSSSGGPLVTVSAALH
QVSPTGLEPSSLLSTEAKLVSATGGPLPPVSTLTALHSLEQTSPGLNQQPQNLIMASLPGVMTIGPGEPASLG
PTFTNTGASTLVIGLASTQAQSVPVINSMGSSLTTLQPVQFSQPLHPSYQQPLMPPVQSHVAQSPFMATMA
QLQSPHALYSHKPEVAQYTHTSLLPQTMLITDTNLSTLASLTPTKQVFTSDTEASSEPGLHEPPSPATTIHIPS
QDPSNIQHLQPAHRLSTSPTVSSSSLVLYQSSDSNGHSHLLPSNHSVIETFISTQMASSSQ
>T01331
MDTKHFLPLDFSTQVNSSSLNSPTGRGSMAVPSLHPSLGPGIGSPLGSPGQLHSPISTLSSPINGMGPPFSVIS
SPMGPHSMSVPTTPTLGFGTGSPQLNSPMNPVSSTEDIKPPLGLNGVLKVPAHPSGNMASFTKHICAICGD
RSSGKHYGVYSCEGCKGFFKRTVRKDLTYTCRDNKDCLIDKRQRNRCQYCRYQKCLAMGMKREAVQEE
RQRGKDRNENEVESTSSANEDMPVEKILEAELAVEPKTETYVEANMGLNPSSPNDPVTNICQAADKQLFT
LVEWAKRIPHFSELPLDDQVILLRAGWNELLIASFSHRSIAVKDGILLATGLHVHRNSAHSAGVGAIFDRVL
TELVSKMRDMQMDKTELGCLRAIVLFNPDSKGLSNPAEVEALREKVYASLEAYCKHKYPEQPGRFAKLLL
RLPALRSIGLKCLEHLFFFKLIGDTPIDTFLMEMLEAPHQAT
>T01429
MAAAAAATPGLGPLQLQDEVAQPLNLSAKPKTSDGKSPASPTSPHMPALRINSGAGPLKASVPAALASPSA
RVSTIGYLNDHDAVTKAIQEARQMKEQLRREQQALDGKVAVVNSIGLSNCRTEKEKTTLESLTQQLAVKQ
NEEGKFSHGMMDFNMSGDSDGSAGVSESRIYRESRGRGSNEPHIKRPMNAFMVWAKDERRKILQAFPDM
HNSNISKILGSRWKAMTNLEKQPYYEEQARLSKQHLEKYPDYKYKPRPKRTCLVDGKKLRIGEYKAIMR
NRRQEMRQYFNVGQQAQIPIATAGVVYPGAIAMAGMPSPHLPSEHSSVSSSPEPGMPVIQSTYGAKGEEP
HIKEEIQAEDINGEIYEEYDEEEEDPDVDYGSDSENHIAGQAN
>T01441
MPPCPPQQNRNRLSQLPVGELGEMELTWQEIMSITELQGLNVPSETSFEPQAPTPYPGPLPPPTYCPCSIHPD
AGFSLPPPSYELPASTPHVPELPYSYGNVAIPVSKPLTLSGLLNEPLPDHLALLDIGLPVGQPKPQEDPESDS

GLSLNYSDAESLELEGMEAGRRRSEYVDMYPVEYPYSLMPNSLAHPNYTLPPTETPLALESSSGPVRAKPA
VRGEAGSRDERRALAMKIPFPTDKIVNLPVDDFNELLAQYPLTESQLALVRDIRRRGKNKVAAQNCRKRK
LETIVQLERELERLSSERERLLRARGEADRTLEVMRQQLAELYHDIFQHLRDESGNSYSPEEYVLQQAADG
AIFLVPRGTKMEATD
>T01574
MAQWNQLQQLDTRYLEQLHQLYSDSFPMELRQFLAPWIESQDWAYAASKESHATLVFHNLLGEIDQQYS
RFLQESNVLYQHNLRRIKQFLQSRYLEKPMEIARIVARCLWEESRLLQTAATAAQQGGQANHPTAAVVTEK
QQMLEQHLQDVRKRVQDLEQKMKVVENLQDDFDFNYKTLKSQGDMQDLNGNNQSVTRQKMQQLEQM
LTALDQMRRSIVSELAGLLSAMEYVQKTLTDEELADWKRRQQIACIGGPPNICLDRLENWITSLAESQLQT
RQQIKKLEELQQKVSYKGDPIVQHRPMLEERIVELFRNLMKSAFVVERQPCMPMHPDRPLVIKTGVQFTT
KVRLLVKFPELNYQLKIKVCIDKDSGDVAALRGSRKFNILGTNTKVINMEESNNGSLSAEFKHLTLREQRC
GNGGRANCDASLIVTEELHLITFETEVYHQGLKIDLETHSLPVVVISNICQMPNAWASILWYNMLTNNPKN
VNFFTKPPIGTWDQVAEVLSWQFSSTTKRGLSIEQLTTLAEKLLGPGVNYSGCQITWAKFCKENMAGKGF
SFWVWLDNIIDLVKKYILALWNEGYIMGFISKERERAILSTKPPGTFLLRFSESSKEGGVTFTWVEKDISGK
TQIQSVEPYTKQQLNNMSFAEIIMGYKIMDATNILVSPLVYLYPDIPKEEAFGKYCRPESQEHPEADPGSAA
PYLKTKFICVTPTTCSNTIDLPMSPRTLDSLMQFGNNGEGAEPSAGGQFESLTFDMDLTSECATSPM
>T01579
MAGWIQAQQLQGDALRQMQVLYGQHFPIEVRHYLAQWIESQPWDAIDLDNPQDRGQATQLLEGLVQEL
QKKAEHQVGEDGFLLKIKLGHYATQLQNTYDRCPMELVRCIRHILYNEQRLVREANNCSSPAGVLVDAMS
QKHLQINQRFEELRLITQDTENELKKLQQTQEYFIIQYQESLRIQAQFAQLGQLNPQERMSRETALQQKQV
SLETWLQREAQTLQQYRVELAEKHQKTLQLLRKQQTIILDDELIQWKRRQQLAGNGGPPEGSLDVLQSW
CEKLAEIIWQNRQQIRRAEHLCQQLPIPGPVEEMLAEVNATITDIISALVTSTFIIEKQPPQVLKTQTKFAATV
RLLVGGKLNVHMNPPQVKATIISEQQAKSLLKNENTRNECSGEILNNCCVMEYHQATGTLSAHFRNMSLK
RIKRADRRGAESVTEEKFTVLFESQFSVGSNELVFQVKTLSLPVVVIVHGSQDHNATATVLWDNAFAEPGR
VPFAVPDKVLWPQLCEALNMKFKAEVQSNRGLTKENLVFLAQKLFNISSNHLEDYNSMSVSWSQFNREN
LPGWNYTFWQWFDGVMEVLKKHHKPHWNDGAILGFVNKQQAHDLLINKPDGTFLLRFSDSEIGGITIAW
KFDSPDRNLWNLKPFTTRDFSIRSLADRLGDLNYLIYVFPDRPKDEVFAKYYTPVLAKAVDGYVKPQIKQ
VVPEFVNASTDAGASATYMDQAPSPVVCPQPHYNMYPPNPDPVLDQDGEFDLDESMDVARHVEELLRRP
MDSLDARLSPPAGLFTSARSSLS
>T01581
MSLWGLISKMSPEKLQRLYVDFPQRLRHLLADWLESQPWEFLVGSDAFCYNMASALLSATVQRLQATAG
EQGKGNSILPHISTLESIYQRDPLKLVATIRQILQGEKKAVIEEFRHLPGPFHRKQEELKFTTPLGRLHHRVRE
TRLLRESLHLGPKTGQVSLQNLIDPPLNGPGPSEDLPTILQGTVGDLETTQPLVLLRIQIWKRQQQLAGNGT
PFEESLAGLQERCESLVEIYSQLHQEIGAASGELEPKTRASLISRLDEVLRTLVTSSFLVEKQPPQVLKTQTK
FQAGVRFLLGLQFLGTSTKPPMVRADMVTEKQARELSLSQGPGTGVESTGEIMNNTVPLENSIPSNCCSAL
FKNLLLKKIKRCERKGTESVTEEKCAVLFSTSFTLGPNKLLIQLQALSLSLVVIVHGNQDNNAKATILWDN
AFSEMDRVPFVVGERVPWEKMCETLNLKFMVEVGTSRGLLPEHFLFLAQKIFNDNSLSVEAFQHRCVSW
SQFNKEILLGRGFTFWQWFDGVLDLTKRCLRSYWSDRLIIGFISKQYVTSLLLNEPDGTFLLRFSDSEIGGIT
IAHVIRGQDGSSQIENIQPFSAKDLSIRSLGDRIRDLAQLKNLYPKKPKDEAFRSHYKPEQMGKDGRGYVS
TTIKMTVERDQPLPTPEPQMPAMVPPYDLGMAPDASMQLSSDMGYPPQSIHSFQSLEESMSVLPSFQEPHL
QMPPNMSQITMPFDQPHPQGLLQCQSQEHAVSSPEPMLWSDVTMVEDSCLTQPVGGFPQGTWVSEDMYP
PLLPPTEQDLTKLLLENQGEGGGSLGSQPLLKPSPYGQSGISLSHLDLRTNPSW
>T01675
MFPSPALTPTPFSVKDILNLEQQQRSLASGDLSARLEATLAPASCMLAAFKPEAYSGPEAAASGLAELRAE

MGPAPSPPKCSPAFPAAPTFYPGAYGDPDPAKDPRADKKELCALQKAVELDKAETDGAERPRARRRRKPR
VLFSQAQVYELERRFKQQRYLSAPERDQLASVLKLTSTQVKIWFQNRRYKCKRQRQDQTLELLGPPPPPA
RRIAVPVLVRDGKPCLGDPAAYAPAYGVGLNAYGYNAYPYPSYGGAACSPGYSCAAYPAAPPAAQPPAAS
ANSNFVNFGVGDLNTVQSPGMPQGNSGVSTLHGIRAW
>T01828
MPHNSIRSGHGGLNQLGGAFVNGRPLPEVVRQRIVDLAHQGVRPCDISRQLRVSHGCVSKILGRYYETGSI
RPGVIGGSKPKVATPKVVEKIGDYKRQNPTMFAWEIRDRLLAEGVCDNDTVPSVSSINRIIRTKVQQPFNLP
MDSCVATKSLSPGHTLIPSSAVTPPESPQSDSLGSTYSINGLLGIAQPGNDNKRKMDDSDQDSCRLSIDSQSS
SSGPRKHLRTDTFSQHHLEALECPFERQHYPEAYASPSHTKGEQGLYPLPLLNSALDDGKATLTSSNTPLGR
NLSTHQTYPVVADPHSPFAIKQETPELSSSSSTPSSLSSSAFLDLQQVGSGGPAGASVPPFNAFPHAASVYG
QFTGQALLSGREMVGPTLPGYPPHIPTSGQGSYASSAIAGMVAGSEYSGNAYSHTPYSSYSEAWRFPNSSL
LSSPYYYSSTSRPSAPPTSATAFDHL
>T01964
MLLEAELDCHRERPGAPGASALCTFSRTPEIPMCAGCDQHILDRFILKALDRHWHSKCLKCSDCHVPLAE
RCFSRGESVYCKDDFFKRFGTKCAACQLGIPPTQVVRRAQDFVYHLHCFACVVCKRQLATGDEFYLMED
SRLVCKADYETAKQREAEATAKRPRTTITAKQLETLKSAYNTSPKPARHVREQLSSETGLDMRVVQVWFQ
NRRAKEKRLKKDAGRQRWGQYFRNMKRSRGSSKSDKDSIQEGQDSDAEVSFTDEPSMADMGPANGLYS
SLGEPAPALGRPVGGLGSFTLDHGGLTGPEQYRELRPGSPYGIPPSPAAPQSLPGPQPLLSSLVYPDTNLSLV
PSGPPGGPPPMRVLAGNGPSSDLSTESSSGYPDFPASPASWLDEVDHAQF
>T02327
MERIPSAQPPPTCLPKAPGLEHGDLSGMDFAHMYQVYKSRRGIKRSEDSKETYKLPHRLIEKKRRDRINEC
IAQLKDLLPEHLKLTTLGHLEKAVVLELTLKHVKALTNLIDQQQQKIIALQSGLQAGDLSGRNLEAGQEMF
CSGFQTCAREVLQYLAKHENTRDLKSSQLVTHLHRVVSELLQGGASRKPLDSAPKAVDLKEKPSFLAKGS
EGPGKNCVPVIQRTFAPSGGEQSGSDTDTDSGYGGELEKGDLRSEQPYFKSDHGRRFAVGERVSTIKQESE
EPPTKKSRMQLSEEEGHFAGSDLMGSPFLGPHPHQPPFCLPFYLIPPSATAYLPMLEKCWYPTSVPVLYPGL
NTSAAALSSFMNPDKIPTPLLLPQRLPSPLAHSSLDSSALLQALKQIPPLNLETKD
>T02361
MPAAHATSSAPTVTLVQLPNGQTVQVHGVIQAAQPSVIQSPQVQTVQISTIAESEDSQESVDSVTDSQKRR
EILSRRPSYRKILNDLSSDAPGVPRIEEEKSEEETSAPAITTVTVPTPIYQTSSGQYIAITQGGAIQLANNGTD
GVQGLQTLTMTNAAATQPGTTILQYAQTTDGQQILVPSNQVVVQAASGDVQTYQIRTAPTSTIAPGVVMA
SSPALPTQPAEEAARKREVRLMKNREAARECRRKKKEYVKCLENRVAVLENQNKTLIEELKALKDLYCHK
SD
>T02529
MVDTEMPFWPTNFGISSVDLSVMEDHSHSFDIKPFTTVDFSSISAPHYEDIPFTRADPMVADYKYDLKLQE
YQSAIKVEPASPPYYSEKTQLYNRPHEEPSNSLMAIECRVCGDKASGFHYGVHACEGCKGFFRRTIRLKLIY
DRCDLNCRIHKKSRNKCQYCRFQKCLAVGMSHNAIRFGRMPQAEKEKLLAEISSDIDQLNPESADLRALA
KHLYDSYIKSFPLTKAKARAILTGKTTDKSPFVIYDMNSLMMGEDKIKFKHITPLQEQSKEVAIRIFQGCQF
RSVEAVQEITEYAKNIPGFINLDLNDQVTLLKYGVHEIIYTMLASLMNKDGVLISEGQGFMTREFLKSLRK
PFGDFMEPKFEFAVKFNALELDDSDLAIFIAVIILSGDRPGLLNVKPIEDIQDNLLQALELQLKLNHPESSQLF
AKVLQKMTDLRQIVTEHVQLLHVIKKTETDMSLHPLLQEIYKDLY
>T02772
MERDERPPSGGGGGGGSAGFLEPPAALPPPPRNGFCQDELAELDPGTNGETDSLTLGQGHIPVSVPDDRAE
QRTCLICGDRATGLHYGIISCEGCKGFFKRSICNKRVYRCSRDKNCVMSRKQRNRCQYCRLLKCLQMGM
NRKAIREDGMPGGRNKSIGPVQISEEEIERIMSGQEFEEEANHWSNHGDSDHSSPGNRASESNQPSPGSTLS

SSRSVELNGFMAFRDQYMGMSVPPHYQYIPHLFSYSGHSPLLPPQARSLDPQSYSLIHQLMSAEDLEPLGT
PMLIEDGYAVTQAELFALLCRLADELLFRQIAWIKKLPFFCELSIKDYTCLLSSTWQELILLSSLTVYSKQIFG
ELADVTAKYSPSDEELHRFSDEGMEVIERLIYLYHKFHQLKVSNEEYACMKAINFLNQDIRGLTSASQLEQ
LNKRYWYICQDFTEYKYTHQPNRFPDLMMCLPEIRYIAGKMVNVPLEQLPLLFKVVLHSCKTSTVKE
>T02983
MQQDGLSSVNQLGGLFVNGRPLPLDTRQQIVQLAIRGMRPCDISRSLKVSNGCVSKILGRYYRTGVLEPK
CIGGSKPRLATPAVVARIAQLKDEYPALFAWEIQHQLCTEGLCTQDKAPSVSSINRVLRALQEDQSLHWTQ
LRSPAVLAPVLPSPHSNCGAPRGPHPGTSHRNRTIFSPGQAEALEKEFQRGQYPDSVARGKLAAATSLPEDT
VRVWFSNRRAKWRRQEKLKWEAQLPGASQDLTVPKNSPGIISAQQSPGSVPSAALPVLEPLSPSFCQLCC
GTAPGRCSSDTSSQAYLQPYWDCQSLLPVASSSYVEFAWPCLTTHPVHHLIGGPGQVPSTHCSNWP
>T03388
MAQRYDDLPHYGGMDGVGIPSTMYGDPHAARSMQPVHHLNHGPPLHSHQYPHTAHTNAMAPSMGSSV
NDALKRDKDAIYGHPLFPLLALIFEKCELATCTPREPGVAGGDVCSSESFNEDIAVFAKQIRAEKPLFSSNPE
LDNLMIQAIQVLRFHLLELEKVHELCDNFCHRYISCLKGKMPIDLVIDDREGGSKSDSEDVTRSANLTDQP
SWNRDHDDTASTRSGGTPGPSSGGHTSHSGDNSSEQGDGLDNSVASPSTGDDDDPDKDKKRHKKRGIFP
KVATNIMRAWLFQHLTHPYPSEEQKKQLAQDTGLTILQVNNWFINARRRIVQPMIDQSNRAVSQGTPYNP
DGQPMGGFVMDGQQHMGIRAPGPMSGMGMNMGMEGQWHYM
>T04176
MDPENKKSATGAAAILDLDPDFEPQSRPRSCTWPLPRPDLATEPHEPSEVEPSLGQKVPTEGHSEPILLPSRL
PEPAGGPQPGILGAVTGPRKGGSRRNAWGNQSYAELISQAIESAPEKRLTLAQIYEWMVRTVPYFKDKGDS
NSSAGWKNSIRHNLSLHSKFIKVHNEATGKSSWWMLNPDGGKGGKAPRRRAASMDSSSKLLRGRSKGP
KKKPSVLPAPPEGATPRSPLGHFAKWSSSPCPRNREEADVWTTFRPRSSSNASTVSTRLSPMRPESEVLAEE
EMPASASSYAGGVPPTLSEDLELLDGLNLASPHSLLSRSGLSGFSLQHPGLAGPLHSYGASLFGPIDGSLSA
GEGCFSSSQSLEALLTSDTPPPPADVLMTQVDPILSQAPTLLLLGGMPSSSKLGTGVSLCPTPLEGPGPSNLV
PNLSVMAPPPVMAGAPIPKVLGTPVLASPTEDSSHDRMPQDLDLDMYMENLECDMDNIISDLMDGEGLD
FNFEPDP
>T04669
MLLDAGPQYPAIGVTTFGASRHHSAGDVAERDVGLGINPFADGMGAFKLNPSSHELASAGQTAFTSQAPG
YRAAAALGHHHHPGHVGSYSSAAFNSTRDFLFRNRGFGDAAAAASAQHSLFAASAGGFGGPHGHTDAA
GHLLFSGLHEQAAGHASPNVVNGQMRLGFSGDMYPRPEQYGQVTSPRSEHYAAPQLHGYGPMNVNMA
AHHGAGAFFRYMRQPIKQELICKWIEPEQLANPKKSCNKTFSTMHELVTHVTVEHVGGPEQSNHICFWEE
CPREGKPFKAKYKLVNHIRVHTGEKPFPCPFPGCGKVFARSENLKIHKRTHTGEKPFKCEFEGCDRRFANS
SDRKKHMHVHTSDKPYLCKMCDKSYTHPSSLRKHMKVHESSSQGSQPSPAASSGYESSTPPTIVSPTTDNP
TTSSMSPSSSAVHHTAGHSALSSNFNEWYV
>T04670
MLLDAGPQFPAIGVGSFARHHHHSAAAAAAAAAEMQDRELSLAAAQNGFVDSAAAHMGAFKLNPGAH
ELSPGQSSAFTSQGPGAYPGSAAAAAAAAALGPHAAHVGSYSGPPFNSTRDFLFRSRGFGDSAPGGGQHG
LFGPGAGGLHHAHSDAQGHLLFPGLPPEQHGPHASQNVLNGQMRLGLPGEVFGRSEQYRQVASPRTDPY
SAAQLHNQYGPMNMNMGMNMAAAAAHHHHHHHHPGAFFRYMRQQCIKQELICKWIDPEQLSNPKKSC
NKTFSTMHELVTHVSVEHVGGPEQSNHVCFWEECPREGKPFKAKYKLVNHIRVHTGEKPFPCPFPGCGKV
FARSENLKIHKRTHTGEKPFQCEFEGCDRRFANSSDRKKHMHVHTSDKPYLCKMCDKSYTHPSSLRKHM
KVHESSPQGSESSPAASSGYESSTPPGLVSPSAEPQSSSNLSPAAAAAAAAAAAARAAVSAVHRGAGSGSS
GSGGARRPAAVGAAAGRAAGAAAALAGAAGTTGGHSGLSSNFNEWYV
>T06585

MPRSFLVKSKKAHTYHQPRAQGDELVWPPAVIPVAKEHSQSASPLLSTPLPSQTLDWNTIKQEREMLLNQS
LPKMASAPEGPLVTPQPQDGESPLSESPPFYKPSFSWDTLASSYSHSYTQTPSTMQSAFLERSVRLYGSPLV
PSTESPLDFRLRYSPGMDTYHCVKCNKVFSTPHGLEVHVRRSHSGTRPFACDVCGKTFGHAVSLEQHTHV
HSQERSFECRMCGKAFKRSSTLSTHLLIHSDTRPYPCQFCGKRFHQKSDMKKHTYIHTGEKPHKCQVCGK
AFSQSSNLITHSRKHTGFKPFSCELCTKGFQRKVDLRRHRESQHNLK
>T08291
MDFPGLGALGTSEPLPQFVDSALVSSPSDSTGFFSSGPEGLDAASSSTSPNAATAAASALAYYREAEAYRHS
PVFQVYPLLNSMEGIPGGSPYASWAYGKTALYPASTVCPSHEDAPSQALEDQEGKSNNTFLDTLKTERLSP
DLLTLGTALPASLPVTGSAYGGADFPSPFFSPTGSPLSSAAYSSPKFHGSLPLAPCEARECVNCGATATPLWR
RDRTGHYLCNACGLYHKMNGQNRPLIRPKKRMIVSKRAGTQCTNCQTTTTTLWRRNASGDPVCNACGL
YFKLHQVNRPLTMRKDGIQTRNRKASGKGKKKRGSNLAGAGAAEGPAGGFMVVAGSSSSGNCGEVASG
LALGTAGTAHLYQGLGPVVLSGPVSHLMPFPGPLLGSPTTSFPTGPAPTTSSTSVIAPLSS
>T00042
MEVQLGLGRVYPRPPSKTYRGAFQNLFQSVREAIQNPGPRHPEAASIAPPGACLQQRQETSPRRRRRQQH
PEDGSPQAHIRGTTGYLALEEEQQPSQQQSASEGHPESGCLPEPGAATAPGKGLPQQPPAPPDQDDSAAPS
TLSLLGPTFPGLSSCSADIKDILSEAGTMQLLQQQQQQQQQQQQQQQQQQQQQQEVISEGSSSVRAREAT
GAPSSSKDSYLGGNSTISDSAKELCKAVSVSMGLGVEALEHLSPGEQLRGDCMYASLLGGPPAVRPTPCAP
LAECKGLSLDEGPGKGTEETAEYSSFKGGYAKGLEGESLGCSGSSEAGSSGTLEIPSSLSLYKSGAVDEAAA
YQNRDYYNFPLALSGPPHPPPPTHPHARIKLENPSDYGSAWAAAAAQCRYGDLASLHGGSVAGPSTGSPPA
TASSSWHTLFTAEEGQLYGPGGGGGSSSPSDAGPVAPYGYTRPPQGLASQEGDFSASEVWYPGGVVNRVP
YPSPSCVKSEMGPWMENYSGPYGDMRLDSTRDHVLPIDYYFPPQKTCLICGDEASGCHYGALTCGSCKV
FFKRAAEGKQKYLCASRNDCTIDKFRRKNCPSCRLRKCYEAGMTLGARKLKKLGNLKLQEEGENSSAGS
PTEDPSQKMTVSHIEGYECQPIFLNVLEAIEPGVVCAGHDNNQPDSFAALLSSLNELGERQLVHVVKWAK
ALPGFRNLHVDDQMAVIQYSWMGLMVFAMGWRSFTNVNSRMLYFAPDLVFNEYRMHKSRMYSQCVR
MRHLSQEFGWLQITPQEFLCMKALLLFSIIPVDGLKNQKFFDELRMNYIKELDRIIACKRKNPTSCSRRFYQ
LTKLLDSVQPIARELHQFTFDLLIKSHMVSVDFPEMMAEIISVQVPKILSGKVKPIYFHTQ
>T00108
MESADFYEAEPRPPMSSHLQSPPHAPSNARLWLSPGRGPRAAPSPTCRPGAAGRICEHETSIDISAYIDPAAF
NDEFLADLFQHSRQQEKAKAAAGPAGGGDFDYPGAPAGPGGAVMSAGAHGPPPGYGCAAAGYLDGRL
EPLYERVGAPALRPLVIKQEPREEDEAKQLALAGLFPYQPPPPPPPPHPHASPAHLAAPHLQFQIAHCGQTT
MHLQPGHPTPPPTPVPSPHPAPAMGAAGLPGPGGSLKGLAGPHPDLRTGGGGGGGAGAGKAKKSVDKNS
NEYRVRRERNNIAVRKSRDKAKQRNVETQQKVLELTSDNDRLRKRVEQLSRELDTLRGIFRQLPESSLVKA
MGNCA
>T00333
MDSKESLAPPGRDEVPGSLLGQGRGSVMDFYKSLRGGATVKVSASSPSVAAASQADSKQQRILLDFSKGS
TSNVQQRQQQQQQQQQQQQQQQQQQPGLSKAVSLSMGLYMGETETKVMGNDLGYPQQGQLGLSSGE
TDFRLLEESIANLNRSTSVPENPKSSTSATGCATPTEKEFPKTHSDASSEQQNRKSQTGTNGGSVKLYPTDQ
STFDLLKDLEFSAGSPSKDTNESPWRSDLLIDENLLSPLAGEDDPFLLEGNTNEDCKPLILPDTKPKIKDTG
DTILSSPSSVALPQVKTEKDDFIELCTPGVIKQEKLGPVYCQASFSGTNIIGNKMSAISVHGVSTSGGQMYH
YDMNTASLSQQQDQKPVFNVIPPIPVGSENWNRCQGSGEDSLTSLGALNFPGRSVFSNGYSSPGMRPDVSS
PPSSSSAATGPPPKLCLVCSDEASGCHYGVLTCGSCKVFFKRAVEGQHNYLCAGRNDCIIDKIRRKNCPACR
YRKCLQAGMNLEARKTKKKIKGIQQATAGVSQDTSENPNKTIVPAALPQLTPTLVSLLEVIEPEVLYAGYD
SSVPDSAWRIMTTLNMLGGRQVIAAVKWAKAILGLRNLHLDDQMTLLQYSWMFLMAFALGWRSYRQSS
GNLLCFAPDLIINEQRMSLPCMYDQCKHMLFVSSELQRLQVSYEEYLCMKTLLLLSSVPKEGLKSQELFD

EIRMTYIKELGKAIVKREGNSSQNWQRFYQLTKLLDSMHEVVENLLTYCFQTFLDKTMSIEFPEMLAEIIT
NQIPKYSNGNIKKLLFHQK
>T00369
MVSKLSQLQTELLAALLESGLSKEALIQALGEPGPYLMVGDGPLDKGESCGGTRGDLTELPNGLGETRGS
EDDTDDDGEDFAPPILKELENLSPEEAAHQKAVVESLLQEDPWRVAKMVKSYLQQHNIPQREVVDTTGLN
QSHLSQHLNKGTPMKTQKRAALYTWYVRKQREVAQQFTHAGQGGLIEEPTGDELPTKKGRRNRFKWGP
ASQQILFQAYERQKNPSKEERETLVEECNRAECIQRGVSPSQAQGLGSNLVTEVRVYNWFANRRKEEAFR
HKLAMDTYNGPPPGPGPGPALPAHSSPGLPTTTLSPSKVHGVRYGQSATSEAAEVPSSSGGPLVTVSAALH
QVSPTGLEPSSLLSTEAKLVSATGGPLPPVSTLTALHSLEQTSPGLNQQPQNLIMASLPGVMTIGPGEPASLG
PTFTNTGASTLVIGLASTQAQSVPVINSMGSSLTTLQPVQFSQPLHPSYQQPLMPPVQSHVAQSPFMATMA
QLQSPHALYSHKPEVAQYTHTSLLPQTMLITDTNLSTLASLTPTKQVFTSDTEASSEPGLHEPSSPATTIHIPS
QDPSNIQHLQPAHRLSTSPTVSSSSLVLYQSSDSNGHSHLLPSNHGVIETFISTQMASSSQ
>T00371
MLGTVKMEGHESNDWNSYYADTQEAYSSVPVSNMNSGLGSMNSMNTYMTMNTMTTSGNMTPASFNM
SYANPGLGAGLSPGAVAGMPGGSAGAMNSMTAAGVTAMGAALSPGGMGSMGAQPAASMNGLGPYAAA
MNPCMSPMAYAPSNLGRSRAGGGGDAKTFKRSYPHAKPPYSYISLITMAIQQAPSKMLTLSEIYQWIMDL
FPYYRQNQQRWQNSIRHSLSFNACFVKVARSPDKPGKGSYWTLHPDSGNMFENGCYLRRQKRFKCEKQP
GAGGGSGGGGSKGVPENRKDPSGPVNPSAESPIHRGVHGKASQLEGAPAPGPAASPQTLDHSGATATGGG
SELKSPASSSAPPISSGPGGWICTPLSPTWLAPHESQLHLKGAPHYSFNHPFSINNLMSSSEQQHKLDFKAY
EQALQYSPYGATLPASLPLGGASVATRSPIEPSALEPAYYQGVYSRPVLNTS
>T00372
MDMADYSAALDPAYTTLEFENVQVLTMGNDTSPSEGANLNSSNSLGVSALCAICGDRATGKHYGASSCD
GCKGFFRRSVRKNHMYSCRFSRQCVVDKDKRNQCRYCRLKKCFRAGMKKEAVQNERDRISTRRSSYED
SSLPSINALLQAEVLSQQITSPISGINGDIRAKRIASITDVCESMKEQLLVLVEWAKYIPAFCELLLDDQVALL
RAHAGEHLLLGATKRSMVFKDVLLLGNDYIVPRHCPELAEMSRVSIRILDELVLPFQELQIDDNEYACLKA
IIFFDPDAKGLSDPGKIKRLRSQVQVSLEDYINDRQYDSRGRFGELLLLLPTLQSITWQMIEQIQFIKLFGMA
KIDNLLQEMLLGGSASDAPHAHHPLHPHLMQEHMGTNVIVANTMPSHLSNGQMSTPETPQPSPPSGSGSE
SYKLLPGAITTIVKPPSAIPQPTITKQEAI
>T00459
MHRLLAWDAACLPPPPAAFRPMEVANFYYEPDCLAYGAKAARAAPRAPAAEPAIGEHERAIDFSPYLEPL
APAAADFAAPAPAHHDFLSDLFADDYGAKPSKKPSDYGYVSLGRAGAKAAPPACFPPPPPAALKAEPGFEP
ADCKRADDAPAMAAGFPFALRAYLGYQATPSGSSGSLSTSSSSSPPGTPSPADAKAAPAACFAGPPAAPAK
AKAKKAVDKLSDEYKMRRERNNIAVRKSRDKAKMRNLETQHKVLELTAENERLQKKVEQLSRELSTLRN
LFKQLPEPLLASAGHC
>T00535
LCLTQDEFHPFIEALLPHVRAFAYTWFNLQARKRKYFKKHEKRMSKEEERAVKDELLSEKPEVKQKWASR
LLAKLRKDIRPEYREDFVLTVTGKKPPCCVLSNPDQKGKMRRIDCLRQADKVWRLDLVMVILFKGIPLES
TDGERLVKSPQCSNPGLCVQPHHIGVSVKELDLYLAYFVHAADSSQSESPSQPSDADIKDQPENGHLGFQD
SFVTSGVFSVTELVRVSQTPIAAGTGPNFSLSDLESSSYYSMSPGAMRRSLPSTSSTSSTKRLKSVEDEMDS
PGEEPFYTGQGRSPGSGSQSSGWHEVEPGMPSPTTLKKSEKSGFSSPSPSQTSSLGTAFTQHHRPVITGPRA
SPHATPSTLHFPTSPIIQQPGPYFSHPAIRYHPQETLKEFVQLVCPDAGQQAGQVGFLNPNGSSQGKVHNPF
LPTPMLPPPPPPPMARPVPLPMPDTKPPTTSTEGGAASPTSPTYSTPSTSPANRFVSVGPRDPSFVNIPQQTQ
SWYLG
>T00599

LCLTQDEFHPFIEALLPHVRAFAYTWFNLQARKRKYFKKHEKRMSKEEERAVKDELLSEKPEVKQKWASR
LLAKLRKDIRPEYREDFVLTVTGKKPPCCVLSNPDQKGKMRRIDCLRQADKVWRLDLVMVILFKGIPLES
TDGERLVKSPQCSNPGLCVQPHHIGVSVKELDLYLAYFVHAADSSQSESPSQPSDADIKDQPENGHLGFQD
SFVTSGVFSVTELVRVSQTPIAAGTGPNFSLSDLESSSYYSMSPGAMRRSLPSTSSTSSTKRLKSVEDEMDS
PGEEPFYTGQGRSPGSGSQSSGWHEVEPGMPSPTTLKKSEKSGFSSPSPSQTSSLGTAFTQHHRPVITGPRA
SPHATPSTLHFPTSPIIQQPGPYFSHPAIRYHPQETLKEFVQLVCPDAGQQAGQVGFLNPNGSSQGKVHNPF
LPTPMLPPPPPPPMARPVPLPMPDTKPPTTSTEGGAASPTSPTYSTPSTSPANRFVSVGPRDPSFVNIPQQTQ
SWYLG
>T00853
MTPNSMTENRLPAWDKQKPHPDRGQDWKLVGMSEACLHRKSHVERRGALKNEQTSSHLIQATWASSIFH
LDPDDVNDQSVSSAQTFQTEEKKCKGYIPSYLDKDELCVVCGDKATGYHYRCITCEGCKGFFRRTIQKSL
HPSYSCKYEGKCIIDKVTRNQCQECRFKKCIYVGMATDLVLDDSKRLAKRKLIEENREKRRREELQKSIGH
KPEPTDEEWELIKTVTEAHVATNAQGSHWKQKRKFLPEDIGQAPIVNAPEGGQVDLEAFSHFTKIITPAITR
VVDFAKKLPMFCELPCEDQIILLKGCCMEIMSLRAAVRYDPDSETLTLNGEMAVTRGQLKNGGLGVVSDA
IFDLGMSLSSFNLDDTEVALLQAVLLMSSDRPGLACVERIEKYQDSFLLAFEHYINYRKHHVTHFWPKLL
MKVTDLRMIGACHASRFLHMKVECPTELFPPLFLEVFED
>T00856
MSMSPKHTTPFSVSDILSPLEESYKKVGMEGGGLGAPLAAYRQGQAAPPAAAMQQHAVGHHGAVTAAY
HMTAAGVPQLSHSAVGGYCNGNLGNMSELPPYQDTMRNSASGPGWYGANPDPRFPAISRFMGPASGMN
MSGMGGLGSLGDVSKNMAPLPSAPRRKRRVLFSQAQVYELERRFKQQKYLSAPEREHLASMIHLTPTQV
KIWFQNHRYKMKRQAKDKAAQQQLQQDSGGGGGGGGGAGCPQQQQAQQQSPRRVAVPVLVKDGKPC
QAGAPAPGAASLQGHAQQQAQQQAQAAQAAAAAISVGSGGAGLGAHPGHQPGSAGQSPDLAHHAASP
AALQGQVSSLSHLNSSGSDYGAMSCSTLLYGRTW
>T01349
MGDTGRDSRSPDSSSPNPLSQGIPPSSPPGPPHTPSAPPPPMPPPPLGSPFPVISSSMGSPGLPPPAPPGFSGPV
SSPQINSTVSLPGGGSGPPEDVKPPVLGVRGLHCPPPPGGPGAGKRLCAICGDRSSGKHYGVYSCEGCKGF
FKRTIRKDLTYSCRDNKDCTVDKRQRNRCQYCRYQKCLATGMKREAVQEERQRGKDKDGDGDGAGGAP
EEMPVDRILEAELAVEQKSDQGVEGPGATGGGGSSPNDPVTNICQAADKQLFTLVEWAKRIPHFSSLPLDD
QVILLRAGWNELLIASFSHRSIDVRDGILLATGLHVHRNSAHSAGVGAIFDRVLTELVSKMRDMRMDKTE
LGCLRAIILFNPDAKGLSNPGEVEILREKVYASLETYCKQKYPEQQGRFAKLLLRLPALRSIGLKCLEHLFFF
KLIGDTPIDTFLMEMLEAPHQLA
>T02288
MGSDLEGAGSSDVPSPLSAAGDDSLGSDGDCAANSPAAGSGAGDLEGGGGERNSSGGASTQDDPEVTDG
SRTQASPVGPCAGSVGGGEGARSKPYTRRPKPPYSYIALIAMAIRDSAGGRLTLAEINEYLMGKFPFFRGS
YTGWRNSVRHNLSLNDCFVKVLRDPSRPWGKDNYWMLNPNSEYTFADGVFRRRRKRLSHRTTVSASGY
GGGSPPGPAGTPQPAPTAGSSPIARSPARQEEGSSPASKFSSSFAIDSILSKPFRSRRDGTRLWGCSYPGALLP
ARRCAPIPRSFPRRPAVPCCRSVLTARRAHAAGVARGRGAARGAPVRGAPLHRGPSQAISRSGDRRRGAPV
LPPTAAHGPAGGRGLRSGSAPVLPGGDAASLTGAWSSAGGSRVELGAPFVMEGTFCHGGWNAGSRAVHF
PPSPRNRDFFSTLHQMC
>T02290
LVKPPYSYIALITMAILQSPQKKLTLSGICEFISNRFPYYREKFPAWQNSIRHNLSLNDCFVKIPREPGNPGKG
NYWTLDPQSEDMFDNGSFLRRRKRFKR
>T00105
MESADFYEAEPRPPMSSHLQSPPHAPSSAAFGFPRGAGPPKPPAPPAAPEPLGGICEHETSIDISAYIDPAAFN

DEFLADLFQHSRQQEKAKAAVGPTGGGGGGDFDYPGAPAGPGGAVMPGGAHGPPPGYGCAAAGYLDGR
LEPLYERVGAPALRPLVIKQEPREEDEAKQLALAGLFPYQPPPPPPPSHPHPHPPPAHLAAPHLQFQIAHCGQ
TTMHLQPGHPTPPPTPVPSPHPAPALGAAGLPGPGSALKGLGAAHPDLRASGGTGAGKAKKSVDKNSNE
YRVRRERNNIAVRKSRDKAKQRNVETQQKVLELTSDNDRLRKRVEQLSRELDTLRGIFRQLPESSLVKAM
GNCA
>T00112
MKAAVDLKPTLTIIKTEKVDLELFPSPDMECADVPLLTPSSKEMMSQALKATFSGFTKEQQRLGIPKDPRQ
WTETHVRDWVMWAVNEFSLKGVDFQKFCMNGAALCALGKDCFLELAPDFVGDILWEHLEILQKEDVKP
YQVNGVNPAYPESRYTSDYFISYGIEHAQCVPPSEFSEPSFITESYQTLHPISSEELLSLKYENDYPSVILRDP
LQTDTLQNDYFAIKQEVVTPDNMCMGRTSRGKLGGQDSFESIESYDSCDRLTQSWSSQSSFNSLQRVPSY
DSFDSEDYPAALPNHKPKGTFKDYVRDRADLNKDKPVIPAAALAGYTGSGPIQLWQFLLELLTDKSCQSFI
SWTGDGWEFKLSDPDEVARRWGKRKNKPKMNYEKLSRGLRYYYDKNIIHKTAGKRYVYRFVCDLQSLL
GYTPEELHAMLDVKPDADE
>T00113
MNDFGIKNMDQVAPVANSYRGTLKRQPAFDTFDGSLFAVFPSLNEEQTLQEVPTGLDSISHDSANCELPLL
TPCSKAVMSQALKATFSGFKKEQRRLGIPKNPWLWSEQQVCQWLLWATNEFSLVNVNLQRFGMNGQML
CNLGKERFLELAPDFVGDILWEHLEQMIKENQEKTEDQYEENSHLTSVPHWINSNTLGFGTEQAPYGMQT
QNYPKGGLLDSMCPASTPSVLSSEQEFQMFPKSRLSSVSVTYCSVSQDFPGSNLNLLTNNSGTPKDHDSPE
NGADSFESSDSLLQSWNSQSSLLDVQRVPSFESFEDDCSQSLCLNKPTMSFKDYIQERSDPVEQGKPVIPAA
VLAGFTGSGPIQLWQFLLELLSDKSCQSFISWTGDGWEFKLADPDEVARRWGKRKNKPKMNYEKLSRGL
RYYYDKNIIHKTSGKRYVYRFVCDLQNLLGFTPEELHAILGVQPDTED
>T00137
MARRPRHSIYSSDEDDEDFEMCDHDYDGLLPKSGKRHLGKTRWTREEDEKLKKLVEQNGTDDWKVIAN
YLPNRTDVQCQHRWQKVLNPELIKGPWTKEEDQRVIELVQKYGPKRWSVIAKHLKGRIGKQCRERWHNH
LNPEVKKTSWTEEEDRIIYQAHKRLGNRWAEIAKLLPGRTDNAIKNHWNSTMRRKVEQEGYLQESSKAS
QPAVATSFQKNSHLMGFAQAPPTAQLPATGQPTVNNDYSYYHISEAQNVSSHVPYPVALHVNIVNVPQPAA
AAIQRHYNDEDPEKEKRIKELELLLMSTENELKGQQVLPTQNHTCSYPGWHSTTIADHTRPHGDSAPVSC
LGEHHSTPSLPADPGSLPEESASPARCMIVHQGTILDNVKNLLEFAETLQFIDSFLNTSSNHENSDLEMPSLT
STPLIGHKLTVTTPFHRDQTVKTQKENTVFRTPAIKRSILESSPRTPTPFKHALAAQEIKYGPLKMLPQTPSH
LVEDLQDVIKQESDESGFVAEFQENGPPLLKKIKQEVESPTDKSGNFFCSHHWEGDSLNTQLFTQTSPVRD
APNILTSSVLMAPASEDEDNVLKAFTVPKNRSLASPLQPCSSTWEPASCGKMEEQMTSSSQARKYVNAFS
ARTLVM
>T00207
MNQPQRMAPVGTDKELSDLLDFSMMFPLPVTNGKGRPASLAGAQFGGSGLEDRPSSGSWGSGDQSSSSF
DPSRTFSEGTHFTESHSSLSSSTFLGPGLGGKSGERGAYASFGRDAGVGGLTQAGFLSGELALNSPGPLSPS
GMKGTSQYYPSYSGSSRRRAADGSLDTQPKKVRKVPPGLPSSVYPPSSGEDYGRDATAYPSAKTPSSTYPA
PFYVADGSLHPSAELWSPPGQAGFGPMLGGGSSPLPLPPGSGPVGSSGSSSTFGGLHQHERMGYQLHGAE
VNGGLPSASSFSSAPGATYGGVSSHTPPVSGADSLLGSRGTTAGSSGDALGKALASIYSPDHSSNNFSSSPS
TPVGSPQGLAGTSQWPRAGAPGALSPSYDGGLHGLQSKIEDHLDEAIHVLRSHAVGTAGDMHTLLPGHG
ALASGFTGPMSLGGRHAGLVGGSHPEDGLAGSTSLMHNHAALPSQPGTLPDLSRPPDSYSGLGRAGATAA
ASEIKREEKEDEENTSAADHSEEEKKELKAPRARTSSTDEVLSLEEKDLRDRERRMANNARERVRVRDINE
AFRELGRMCQMHLKSDKAQTKLLILQQAVQVILGLEQQVRERNLNPKAACLKRREEEKVSGVVGDPQM
VLSAPHPGLSEAHNPAGHM
>T00250

MDPSVTLWQFLLQLLREQGNGHIISWTSRDGGEFKLVDAEEVARLWGLRKNKTNMNYDKLSRALRYYY
DKNIIRKVSGQKFVYKFVSYPEVAGCSTEDCPPQPEVSVTSTMPNVAPAAIHAAPGDTVSGKPGTPKGAG
MAGPGGLARSSRNEYMRSGLYSTFTIQSLQPQPPPHPRPAVVLPNAAPAGAAAPPSGSRSTSPSPLEACLEA
EEAGLPLQVILTPPEAPNLKSEELNVEPGLGRALPPEVKVEGPKEELEVAGERGFVPETTKAEPEVPPQEGV
PARLPAVVMDTAGQAGGHAASSPEISQPQKGRKPRDLELPLSPSLLGGPGPERTPGSGSGSGLQAPGPALTP
SLLPTHTLTPVLLTPSSLPPSIHFWSTLSPIAPRSPAKLSFQFPSSGSAQVHIPSISVDGLSTPVVLSPGPQKP
>T00311
MEVTADQPRWVSHHHPAVLNGQHPDTHHPGLSHSYMDAAQYPLPEEVDVLFNIDGQGNHVPPYYGNSV
RATVQRYPPTHHGSQVCRPPLLHGSLPWLDGGKALGSHHTASPWNLSPFSKTSIHHGSPGPLSVYPPASSSS
LSGGHASPHLFTFPPTPPKDVSPDPSLSTPGSAGSARQDEKECLKYQVPLPDSMKLESSHSRGSMTALGGA
SSSTHHPITTYPPYVPEYSSGLFPPSSLLGGSPTGFGCKSRPKARSSTGRECVNCGATSTPLWRRDGTGHYL
CNACGLYHKMNGQNRPLIKPKRRLSAARRAGTSCANCQTTTTTLWRRNANGDPVCNACGLYYKLHNIN
RPLTMKKEGIQTRNRKMSSKSKKCKKVHDSLEDFPKNSSFNPAALSRHMSSLSHISPFSHSSHMLTTPTPM
HPPSSLSFGPHHPSSMVTAMG
>T00330
MFNSMTPPPISSYGEPCCLRPLPSQGAPSVGTEGLSGPPFCHQANLMSGPHSYGPARETNSCTEGPLFSSPR
SAVKLTKKRALSISPLSDASLDLQTVIRTSPSSLVAFINSRCTSPGGSYGHLSIGTMSPSLGFPAQMNHQKGPS
PSFGVQPCGPHDSARGGMIPHPQSRGPFPTCQLKSELDMLVGKCREEPLEGDMSSPNSTGIQDPLLGMLDG
REDLEREEKREPESVYETDCRWDGCSQEFDSQEQLVHHINSEHIHGERKEFVCHWGGCSRELRPFKAQYM
LVVHMRRHTGEKPHKCTFEGCRKSYSRLENLKTHLRSHTGEKPYMCEHEGCSKAFSNASDRAKHQNRTH
SNEKPYVCKLPGCTKRYTDPSSLRKHVKTVHGPDAHVTKRHRGDGPLPRAPSISTVEPKREREGGPIREES
RLTVPEGAMKPQPSPGAQSSCSSDHSPAGSAANTDSGVEMTGNAGGSTEDLSSLDEGPCIAGTGLSTLRRL
ENLRLDQLHQLRPIGTRGLKLPSLSHTGTTVSRRVGPPVSLERRSSSSSSISSAYTVSRRSSLASPFPPGSPPE
NGASSLPGLMPAQHYLLRARYASARGGGTSPTAASSLDRIGGLPMPPWRSRAEYPGYNPNAGVTRRASDP
AQAADRPAPARVQRFKSLGCVHTPPTVAGGGQNFDPYLPTSVYSPQPPSITENAAMDARGLQEEPEVGTS
MVGSGLNPYMDFPPTDTLGYGGPEGAAAEPYGARGPGSLPLGPGPPTNYGPNPCPQQASYPDPTQETWG
EFPSHSGLYPGPKALGGTYSQCPRLEHYGQVQVKPEQGCPVGSDSTGLAPCLNAHPSEGPPHPQPLFSHYP
QPSPPQYLQSGPYTQPPPDYLPSEPRPCLDFDSPTHSTGQLKAQLVCNYVQSQQELLWEGGGREDAPAQEP
SYQSPKFLGGSQVSPSRAKAPVNTYGPGFGPNLPNHKSGSYPTPSPCHENFVVGANRASHRAAAPPRLLPP
LPTCYGPLKVGGTNPSCGHPEVGRLGGGPALYPPPEGQVCNPLDSLDLDNTQLDFVAILDEPQGLSPPPSH
DQRGSSGHTPPPSGPPNMAVGNMSVLLRSLPGETEFLNSSA
>T00331
MEAQSHSSTTTEKKKVENSIVKCSTRTDVSEKAVASSTTSNEDESPGQTYHRERRNAITMQPQNVQGLSK
VSEEPSTSSDERASLIKKEIHGSLPHVAEPSVPYRGTVFAMDPRNGYMEPHYHPPHLFPAFHPPVPIDARHH
EGRYHYDPSPIPPLHMTSALSSSPTYPDLPFIRISPHRNPAAASESPFSPPHPYINPYMDYIRSLHSSPSLSMIS
ATRGLSPTDAPHAGVSPAEYYHQMALLTGQRSPYADIIPSAATAGTGAIHMEYLHAMDSTRFSSPRLSARP
SRKRTLSISPLSDHSFDLQTMIRTSPNSLVTILNNSRSSSSASGSYGHLSASAISPALSFTYSSAPVSLHMHQQI
LSRQQSLGSAFGHSPPLIHPAPTFPTQRPIPGIPTVLNPVQVSSGPSESSQNKPTSESAVSSTGDPMHNKRSKI
KPDEDLPSPGARGQQEQPEGTTLVKEEGDKDESKQEPEVIYETNCHWEGCAREFDTQEQLVHHINNDHIH
GEKKEFVCRWLDCSREQKPFKAQYMLVVHMRRHTGEKPHKCTFEGCTKAYSRLENLKTHLRSHTGEKP
YVCEHEGCNKAFSNASDRAKHQNRTHSNEKPYVCKIPGCTKRYTDPSSLRKHVKTVHGPEAHVTKKQRG
DIHPRPPPPRDSGSHSQSRSPGRPTQGALGEQQDLSNTTSKREECLQVKTVKAEKPMTSQPSPGGQSSCSS
QQSPISNYSNSGLELPLTDGGSIGDLSAIDETPIMDSTISTATTALALQARRNPAGTKWMEHVKLERLKQVN
GMFPRLNPILPPKAPAVSPLIGNGTQSNNTCSLGGPMTLLPGRSDLSGVDVTMLNMLNRRDSSASTISSAYL

SSRRSSGISPCFSSRRSSEASQAEGRPQNVSVADSYDPISTDASRRSSEASQSDGLPSLLSLTPAQQYRLKAK
YAAATGGPPPTPLPNMERMSLKTRLALLGDALEPGVALPPVHAPRRCSDGGAHGYGRRHLQPHDALGHG
VRRASDPVRTGSEGLALPRVPRFSSLSSCNPPAMATSAEKRSLVLQNYTRPEGGQSRNFHSSPCPPSITENVT
LESLTMDADANLNDEDFLPDDVVQYLNSQNQAGYEQHFPSALPDDSKVPHGPGDFDAPGLPDSHAGQQF
HALEQPCPEGSKTDLPIQWNEVSSGSADLSSSKLKCGPRPAVPQTRAFGFCNGMVVHPQNPLRSGPAGGY
QTLGENSNPYGGPEHLMLHNSPGSGTSGNAFHEQPCKAPQYGNCLNRQPVAPGALDGACGAGIQASKLK
STPMQGSGGQLNFGLPVAPNESAGSMVNGMQNQDPVGQGYLAHQLLGDSMQHPGAGRPGQQMLGQIS
ATSHINIYQGPESCLPGAHGMGSQPSSLAVVRGYQPCASFGGSRRQAMPRDSLALQSGQLSDTSQTCRVN
GIKMEMKGQPHPLCSNLQNYSGQFYDQTVGFSQQDTKAGSFSISDASCLLQGTSAKNSELLSPGANQVTS
TVDSLDSHDLEGVQIDFDAIIDDGDHSSLMSGALSPSIIQNLSHSSSRLTTPRASLPFPVAVHEHHQHGYRGH
EFFADLPSGRKQIPCSYAIGFRKKRLQPTEINRS
>T00337
MDSKESLTPGREENPSSVLAQERGDVMDFYKTLRGGATVKVSASSPSLAVASQSDSKQRRLLVDFPKGSV
SNAQQPDLSKAVSLSMGLYMGETETKVMGNDLGFPQQGQISLSSGETDLKLLEESIANLNRSTSVPENPKS
SASTAVSAAPTEKEFPKTHSDVSSEQQHLKGQTGTNGGNVKLYTTDQSTFDILQDLEFSSGSPGKETNESP
WRSDLLIDENCLLSPLAGEDDSFLLEGNSNEDCKPLILPDTKPKIKDNGDLVLSSPSNVTLPQVKTEKEDFI
ELCTPGVIKQEKLGTVYCQASFPGANIIGNKMSAISVHGVSTSGGQMYHYDMNTASLSQQQDQKPIFNVIP
PIPVGSENWNRCQGSGDDNLTSLGTLNFPGRTVFSNGYSSPSMRPDVSSPPSSSSTATTGPPPKLCLVCSDE
ASGCHYGVLTCGSCKVFFKRAVEGQHNYLCAGRNDCIIDKIRRKNCPACRYRKCLQAGMNLEARKTKKK
IKGIQQATTGVSQETSENPGNKTIVPATLPQLTPTLVSLLEVIEPEVLYAGYDSSVPDSTWRIMTTLNMLGGR
QVIAAVKWAKAIPGFRNLHLDDQMTLLQYSWMFLMAFALGWRSYRQSSANLLCFAPDLIINEQRMTLPC
MYDQCKHMLYVSSELHRLQVSYEEYLCMKTLLLLSSVPKDGLKSQELFDEIRMTYIKELGKAIVKREGNS
SQNWQRFYQLTKLLDSMHEVVENLLNYCFQTFLDKTMSIEFPEMLAEIITNQIPKYSNGNIKKLLFHQK
>T00368
MVSKLSQLQTELLAALLESGLSKEALIQALGEPGPYLLAGEGPLDKGESCGGGRGELAELPNGLGETRGS
EDETDDDGEDFTPPILKELENLSPEEAAHQKAVVETLLQEDPWRVAKMVKSYLQQHNIPQREVVDTTGLN
QSHLSQHLNKGTPMKTQKRAALYTWYVRKQREVAQQFTHAGQGGLIEEPTGDELPTKKGRRNRFKWGP
ASQQILFQAYERQKNPSKEERETLVEECNRAECIQRGVSPSQAQGLGSNLVTEVRVYNWFANRRKEEAFR
HKLAMDTYSGPPPGPGPGPALPAHSSPGLPPPALSPSKVHGVRYGQPATSETAEVPSSSGGPLVTVSTPLHQ
VSPTGLEPSHSLLSTEAKLVSAAGGPLPPVSTLTALHSLEQTSPGLNQQPQNLIMASLPGVMTIGPGEPASL
GPTFTNTGASTLVIGLASTQAQSVPVINSMGSSLTTLQPVQFSQPLHPSYQQPLMPPVQSHVTQSPFMATM
AQLQSPHALYSHKPEVAQYTHTGLLPQTMLITDTTNLSALASLTPTKQVFTSDTEASSESGLHTPASQATTL
HVPSQDPAGIQHLQPAHRLSASPTVSSSSLVLYQSSDSSNGQSHLLPSNHSVIETFISTQMASSSQ
>T00529
MNGPLVYAGFALQLGSISAGPGSVSPHLHVPWDLGMAGLSGQIQSPSREGGFAHRVLLPSDLRSEQDPTDE
DPCRGVGPALITTRWRSPRGRSRGRPSTGGGVVRGGRCDVCGKVFSQRSNLLRHQKIHTGERPFVCSECG
RSFSRSSHLLRHQLTHTEERPFVCGDCGQGFVRSARLEEHRRVHTGEQPFRCAECGQSFRQRSNLLQHQRI
HGDPPGPGAKPPAPPGAPEPPGPFPCSECRESFARRAVLLEHQAVHTGDKSFGCVECGERFGRRSVLLQHR
RVHSGERPFACAECGQSFRQRSNLTQHRRIHTGERPFACAECGKAFRQRPTLTQHLRVHTGEKPFACPECG
QRFSQRLKLTRHQRTHTGEKPYHCGECGLGFTQVSRLTEHQRIHTGERPFACPECGQSFRQHANLTQHRRI
HTGERPYACPECGKAFRQRPTLTQHLRTHRREKPFACQDCGRRFHQSTKLIQHQRVHSAE
>T00539
MDEFHPFIEALLPHVRAFAYTWFNLQARKRKYFKKHEKRMSKDEERAVKDELLGEKPEVKQKWASRLLA
KLRKDIRPECREDFVLSITGKKAPGCVLSNPDQKGKMRRIDCLRQADKVWRLDLVMVILFKGIPLESTDG

ERLVKAAQCGHPVLCVQPHHIGVAVKELDLYLAYFVRERDAEQSGSPRTGMGSDQEDSKPITLDTTDFQES
FVTSGVFSVTELIQVSRTPVVTGTGPNFSLGELQGHLAYDLNPASTGLRRTLPSTSSSGSKRHKSGSMEEDV
DTSPGGDYYTSPSSPTSSSRNWTEDMEGGISSPVKKTEMDKSPFNSPSPQDSPRLSSFTQHHRPVIAVHSGI
ARSPHPSSALHFPTTSILPQTASTYFPHTAIRYPPHLNPQDPLKDLVSLACDPASQQPGPLNGSGQLKMPSHC
LSAQMLAPPPPGLPRLALPPATKPATTSEGGATSPTSPSYSPPDTSPANRSFVGLGPRDPAGIYQAQSWYLG
>T00581
MQRLVAWDPACLPLPPPPPAFKSMEVANFYYEADCLAAAYGGKAAPAAPPAARPGPRPPAGELGSIGDHER
AIDFSPYLEPLGAPQAPAPATATDTFEAAPPAPAPAPASSGQHHDFLSDLFSDDYGGKNCKKPAEYGYVSLG
RLGAAKGALHPGCFAPLHPPPPPPPPPAELKAEPGFEPADCKRKEEAGAPGGGAGMAAGFPYALRAYLGY
QAVPSGSSGSLSTSSSSSPPGTPSPADAKAPPTACYAGAGPAPSQVKSKAKKTVDKHSDEYKIRRERNNIAV
RKSRDKAKMRNLETQHKVLELTAENERLQKKVEQLSRELSTLRNLFKQLPEPLLASSGHC
>T00625
MADGPRCKRRKQANPRRNNVTNYNTVVETNSDSDDEDKLHIVEEESVTDAADCEGVPEDDLPTDQTVLP
GRSSEREGNAKNCWEDDRKEGQEILGPEAQADEAGCTVKDDECESDAENEQNHDPNVEEFLQQQDTAVI
FPEAPEEDQRQGTPEASGHDENGTPDAFSQLLTCPYCDRGYKRFTSLKEHIKYRHEKNEDNFSCSLCSYTF
AYRTQLERHMTSHKSGRDQRHVTQSGCNRKFKCTECGKAFKYKHHLKEHLRIHSGEKPYECPNCKKRFS
HSGSYSSHISSKKCISLIPVNGRPRTGLKTSQCSSPSLSASPGSPTRPQIRQKIENKPLQEQLSVNQIKTEPVD
YEFKPIVVASGINCSTPLQNGVFTGGGPLQATSSPQGMVQAVVLPTVGLVSPISINLSDIQNVLKVAVDGNVI
RQVLENNQANLASKEQETINASPIQQGGHSVISAISLPLVDQDGTTKIIINYSLEQPSQLQVVPQNLKKENPV
ATNSCKSEKLPEDLTVKSEKDKSFEGGVNDSTCLLCDDCPGDINALPELKHYDLKQPTQPPPLPAAEAEKP
ESSVSSATGDGNLSPSQPPLKNLLSLLKAYYALNAQPSAEELSKIADSVNLPLDVVKKWFEKMQAGQISVQ
SSEPSSPEPGKVNIPAKNNDQPQSANANEPQDSTVNLQSPLKMTNSPVLPVGSTTNGSRSSTPSPSPLNLSSS
RNTQGYLYTAEGAQEEPQVEPLDLSLPKQQGELLERSTITSVYQNSVYSVQEEPLNLSCAKKEPQKDSCVT
DSEPVVNVIPPSANPINIAIPTVTAQLPTIVAIADQNSVPCLRALAANKQTILIPQVAYTYSTTVSPAVQEPPLK
VIQPNGNQDERQDTSSEGVSNVEDQNDSDSTPPKKKMRKTENGMYACDLCDKIFQKSSSLLRHKYEHTG
KRPHECGICKKAFKHKHHLIEHMRLHSGEKPYQCDKCGKRFSHSGSYSQHMNHRYSYCKREAEERDSTE
QEEAGPEILSNEHVGARASPSQGDSDERESLTREEDEDSEKEEEEEDKEMEELQEEKECEKPQGDEEEEEE
EEEVEEEEVEEAENEGEEAKTEGLMKDDRAESQASSLGQKVGESSEQVSEEKTNEA
>T00641
MNNPSETSKPSMESGDGNTGTQTNGLDFQKQPVPVGGAISTAQAQAFLGHLHQVQLAGTSLQAAAQSLN
VQSKSNEESGDSQQPSQPSQQPSVQAAIPQTQLMLAGGQITGLTLTPAQQQLLLQQAQAQAQLLAAAVQQ
HSASQQHSAAGATISASAATPMTQIPLSQPIQIAQDLQQLQQLQQQNLNLQQFVLVHPTTNLQPAQFIISQTP
QGQQGLLQAQNLQTQLPQQSQANLLQSQPSITLTSQPATPTRTIAATPIQTLPQSQSTPKRIDTPSLEEPSDLE
ELEQFAKTFKQRRIKLGFTQGDVGLAMGKLYGNDFSQTTISRFEALNLSFKNMCKLKPLLEKWLNDAENL
SSDSSLSSPSALNSPGIEGLSRRRKKRTSIETNIRVALEKSFLENQKPTSEEITMIADQLNMEKEVIRVWFCNR
RQKEKRINPPSSGGTSSSPIKAIFPSPTSLVATTPSLVTSSAATTLTVSPVLPLTSAAVTNLSVTGTSDTTSNNTA
TVISTAPPASSAVTSPSLSPSPSASASTSEASSASETSTTQTTSTPLSSPLGTSQVMVTASGLQTAAAAALQGA
AQLPANASLAAMAAAAGLNPSLMAPSQFAAGGALLSLNPGTLSGALSPALMSNSTLATIQALASGGSLPIT
SLDATGNLVFANAGGAPNIVTAPLFLNPQNLSLLTSNPVSLVSAAAASAGNSAPVASLHATSTSAESIQNSLF
TVASASGAASTTTTASKAQ
>T00647
MVHSSMGAPEIRMSKPLEAEKQGLDSPSEHTDTERNGPDTNHQNPQNKTSPFSVSPTGPSTKIKAEDPSGD
SAPAAPLPPQPAQPHLPQAQLMLTGSQLAGDIQQLLQLQQLVLVPGHHLQPPAQFLLPQAQQSQPGLLPTP
NLFQLPQQTQGALLTSQPRAGLPTQAVTRPTLPDPHLSHPQPPKCLEPPSHPEEPSDLEELEQFARTFKQRRI

KLGFTQGDVGLAMGKLYGNDFSQTTISRFEALNLSFKNMCKLKPLLEKWLNDAETMSVDSSLPSPNQLSS
PSLGFDGLPGRRRKKRTSIETNVRFALEKSFLANQKPTSEEILLIAEQLHMEKEVIRVWFCNRRQKEKRINP
CSAAPMLPSPGKPASYSPHMVTPQGGAGTLPLSQASSSLSTTVTTLSSAVGTLHPSRTAGGGGGGGGAAPP
LNSIPSVTPPPPATTNSTNPSPQGSHSAIGLSGLNPSTGPGLWWNPAPYQP
>T00759
MSDQDHSMDEMTAVVKIEKGVGGNNGGNGNGGGAFSQARSSSTGSSSSTGGGGQESQPSPLALLAATCS
RIESPNENSNNSQGPSQSGGTGELDLTATQLSQGANGWQIISSSSGATPTSKEQSGSSTNGSNGSESSKNRTV
SGGQYVVAAAPNLQNQQVLTGLPGVMPNIQYQVIPQFQTVDGQQLQFAATGAQVQQDGSGQIQIIPGANQ
QIITNRGSGGNIIAAMPNLLQQAVPLQGLANNVLSGQTQYVTNVPVALNGNITLLPVNSVSAATLTPSSQAV
TISSSGSQESGSQPVTSGTTISSASLVSSQASSSSFFTNANSYSTTTTTSNMGIMNFTTSGSSGTNSQGQTPQR
VSGLQGSDALNIQQNQTSGGSLQAGQQKEGEQNQQTQQQQILIQPQLVQGGQALQALQAAPLSGQTFTT
QAISQETLQNLQLQAVPNSGPIIIRTPTVGPNGQVSWQTLQLQNLQVQNPQAQTITLAPMQGVSLGQTSSS
NTTLTPIASAASIPAGTVTVNAAQLSSMPGLQTINLSALGTSGIQVHPIQGLPLAIANAPGDHGAQLGLHGA
GGDGIHDDTAGGEEGENSPDAQPQAGRRTRREACTCPYCKDSEGRGSGDPGKKKQHICHIQGCGKVYGK
TSHLRAHLRWHTGERPFMCTWSYCGKRFTRSDELQRHKRTHTGEKKFACPECPKRFMRSDHLSKHIKTH
QNKKGGPGVALSVGTLPLDSGAGSEGSGTATPSALITTNMVAMEAICPEGIARLANSGINVMQVADLQSINI
SGNGF
>T00851
MTPNSMTENGLTAWDKPKHCPDREHDWKLVGMSEACLHRKSHSERRSTLKNEQSSPHLIQTTWTSSIFHL
DHDDVNDQSVSSAQTFQTEEKKCKGYIPSYLDKDELCVVCGDKATGYHYRCITCEGCKGFFRRTIQKNLH
PSYSCKYEGKCVIDKVTRNQCQECRFKKCIYVGMATDLVLDDSKRLAKRKLIEENREKRRREELQKSIGH
KPEPTDEEWELIKTVTEAHVATNAQGSHWKQKRKFLPEDIGQAPIVNAPEGGKVDLEAFSHFTKIITPAITR
VVDFAKKLPMFCELPCEDQIILLKGCCMEIMSLRAAVRYDPESETLTLNGEMAVTRGQLKNGGLGVVSDA
IFDLGMSLSSFNLDDTEVALLQAVLLMSSDRPGLACVERIEKYQDSFLLAFEHYINYRKHHVTHFWPKLL
MKVTDLRMIGACHASRFLHMKVECPTELFPPLFLEVFED
>T00874
MKGQQKTAETEEGTVQIQEGAVATGEDPTSVAIASIQSAATFPDPNVKYVFRTENGGQVMYRVIQVSEGQL
DGQTEGTGAISGYPATQSMTQAVIQGAFTSDDAVDTEGTAAETHYTYFPSTAVGDGAGGTTSGSTAAVVTT
QGSEALLGQATPPGTGQFFVMMSPQEVLQGGSQRSIAPRTHPYSPKSEAPRTTRDEKRRAQHNEVERRRR
DKINNWIVQLSKIIPDCSMESTKSGQSKGGILSKACDYIQELRQSNHRLSEELQGLDQLQLDNDVLRQQVE
DLKNKNLLLRAQLRHHGLEVVIKNDSN
>T00902
MVVVAAAPNPADGTPKVLLLSGQPASAAGAPAARLPLMVPAQRGASPEAASGGLPQARKRQRLTHLSPEE
KALRRKLKNRVAAQTARDRKKARMSELEQQVVDLEEENQKLLLENQLLREKTHGLVVENQELRQRLGM
DALVAEEEAEAKGNEVRPVAGSAESAALRLRAPLQQVQAQLSPLQNISPWILAVLTLQIQSLISCWAFWTT
WTQSCSSNALPQSLPAWRSSQRSTQKDPVPYQPPFLCQWGRHQPSWKPLMN
>T00915
MASGDTLYIATDGSEMPAEIVELHEIEVETIPVETIETTVVGEEEEEDDDDEDGGGGDHGGGGGHGHAGH
HHHHHHHHHHPPMIALQPLVTDDPTQVHHHQEVILVQTREEVVGGDDSDGLRAEDGFEDQILIPVPAPAG
GDDDYIEQTLVTVAAAGKSGGGGSSSSGGGRVKKGGGKKSGKKSYLSGGAGAAGGRGADPGNKKWEQ
KQVQIKTLEGEFSVTMWSSDEKKDIDHETVVEEQIIGENSPPDYSEYMTGKKLPPGGIPGIDLSDPKQLAEF
ARMKPRKIKEDDAPRTIACPHKGCTKMFRDNSAMRKHLHTHGPRVHVCAECGKAFVESSKLKRHQLVHT
GEKPFQCTFEGCGKRFSLDFNLKTHVKIHTGEKPYVCPFEGCNKKFAQSTNLKSHILTHAKAKNNQ
>T01005

MGRKKIQITRIMDERNRQVTFTKRKFGLMKKAYELSVLCDCEIALIIFNSSNKLFQYASTDMDKVLLKYTE
YNEPHESRTNSDIVEALNKKEHRGCDSPDPDTSYVLTPHTEEKYKKINEEFDNMMRNHKIAPGLPPQNFS
MSVTVPVTSPNALSYTNPGSSLVSPSLAASSTLTDSSMLSPPQTTLHRNVSPGAPQRPPSTGNAGGMLSTT
DLTVPNGAGSSPVGNGFVNSRASPNLIGATGANSLGKVMPTKSPPPPGGGNLGMNSRKPDLRVVIPPSSKG
MMPPLSEEEELELNTQRISSSQATQPLATPVVSVTTPSLPPQGLVYSAMPTAYNTDYSLTSADLSALQGFNS
PGMLSLGQVSAWQQHHLGQAALSSLVAGGQLSQGSNLSINTNQNISIKSEPISPPRDRMTPSGFQQQQQQ
QQQQPPPPPQPQPQPPQPQPRQEMGRSPVDSLSSSSSSYDGSDREDPRGDFHSPIVLGRPPNTEDRESPSVK
RMRMDAWVT
>T01042
MDLPVGPGAAGPSNVPAFLTKLWTLVSDPDTDALICWSPSGNSFHVFDQGQFAKEVLPKYFKHNNMASF
VRQLNMYGFRKVVHIEQGGLVKPERDDTEFQHPCFLRGQEQLLENIKRKVTSVSTLKSEDIKIRQDSVTKL
LTDVQLMKGKQECMDSKLLAMKHENEALWREVASLRQKHAQQQKVVNKLIQFLISLVQSNRILGVKRKI
PLMLNDSGSAHSMPKYSRQFSLEHVHGSGPYSAPSPAYSSSSLYAPDAVASSGPIISDITELAPASPMASPGG
SIDERPLSSSPLVRVKEEPPSPPQSPRVEEASPGRPSSVDTLLSPTALIDSILRESEPAPASVTALTDARGHTDT
EGRPPSPPPTSTPEKCLSVACLDKNELSDHLDAMDSNLDNLQTMLSSHGFSVDTSALLDLFSPSVTVPDMS
LPDLDSSLASIQELLSPQEPPRPPEAENSSPDSGKQLVHYTAQPLFLLDPGSVDTGSNDLPVLFELGEGSYFS
EGDGFAEDPTISLLTGSEPPKAKDPTVS
>T01345
MDTKHFLPLDFSTQVNSSLTSPTGRGSMAAPSLHPSLGPGIGSPGQLHSPISTLSSPINGMGPPFSVISSPMGP
HSMSVPTTPTLGFSTGSPQLSSPMNPVSSSEDIKPPLGLNGVLKVPAHPSGNMASFTKHICAICGDRSSGKH
YGVYSCEGCKGFFKRTVRKDLTYTCRDNKDCLIDKRQRNRCQYCRYQKCLAMGMKREAVQEERQRGK
DRNENEVESTSSANEDMPVERILEAELAVEPKTETYVEANMGLNPSSPNDPVTNICQAADKQLFTLVEWA
KRIPHFSELPLDDQVILLRAGWNELLIASFSHRSIAVKDGILLATGLHVHRNSAHSAGVGAIFDRVLTELVSK
MRDMQMDKTELGCLRAIVLFNPDSKGLSNPAEVEALREKVYASLEAYCKHKYPEQPGRFAKLLLRLPALR
SIGLKCLEHLFFFKLIGDTPIDTFLMEMLEAPHQMT
>T01346
MAATTANPEMTSDVPSLGPAIASGNSGPGIQGGGAIVQRAIKRRPGLDFDDDGEGNSKFLRCDDDQMSND
KERFARSDDEQSSADKERLARENHSEIERRRRNKMTAYITELSDMVPTCSALARKPDKLTILRMAVSHMKS
LRGTGNTSTDGSYKPSFLTDQELKHLILEAADGFLFIVSCETGRVVYVSDSVTPVLNQPQSEWFGSTLYDQ
VHPDDVDKLREQLSTSENALTGRILDLKTGTVKKEGQQSSMRMCMGSRRSFICRMRCGSSSVDPVSVNRL
SFVRNRCRNGLGSVKDGEPHFVVVHCTGYIKAWPPAGVSLPDDDPEAGQGSKFCLVAIGRLQVTSSPNCT
DMSNVCQPTEFISRHNIEGIFTFVDHRCVATVGYQPQELLGKNIVEFCHPEDQQLLRDSFQQVVKLKGQVL
SVMFRFRSKNQEWLWMRTSSFTFQNPYSDEIEYIICTNTNVKNSSQEPRPTLSNTIQRPQLGPTANLPLEMG
SGQLAPRQQQQQTELDMVPGRDGLASYNHSQVVQPVTTTGPEHSKPLEKSDGLFAQDRDPRFSEIYHNIN
ADQSKGISSSTVPATQQLFSQGNTFPPTPRPAENFRNSGLAPPVTIVQPSASAGQMLAQISRHSNPTQGATPT
WTPTTRSGFSAQQVATQATAKTRTSQFGVGSFQTPSSFSSMSLPGAPTASPGAAAYPSLTNRGSNFAPETGQ
TAGQFQTRTAEGVGVWPQWQGQQPHHRSSSSEQHVQQPPAQQPGQPEVFQEMLSMLGDQSNSYNNEEFP
DLTMFPPFSE
>T01427
MAENVVEPGPPSAKRPKLSSPALSASASDGTDFGSLFDLEHDLPDELINSTELGLTNGGDINQLQTSLGMV
QDAASKHKQLSELLRSGSSPNLNMGVGGPGQVMASQAQQSSPGLGLINSMVKSPMTQAGLTSPNMGMG
TSGPNQGPTQSTGMMNSPVNQPAMGMNTGTNAGMNPGMLAAGNGQGIMPNQVMNGSIGAGRGRQDM
QYPNPGMGSAGNLLTEPLQQGSPQMGGQTGLRGPQPLKMGMMNNPNPYGSPYTQNPGQQIGASGLGLQ
IQTKTVLSNNLSPFAMDKKAVPGGGMPNMGQQPAPQVQQPGLVTPVAQGMGSGAHTADPEKRKLIQQQL

VLLLHAHKCQRREQANGEVRQCNLPHCRTMKNVLNHMTHCQSGKSCQVAHCASSRQIISHWKNCTRHD
CPVCLPLKNAGDKRNQQPILTGAPVGLGNPSSLGVGQQSAPNLSTVSQIDPSSIERAYAALGLPYQVNQMP
TQPQVQAKNQQNQQPGQSPQGMRPMSNMSASPMGVNGGVGVQTPSLLSDSMLHSAINSQNPMMSENA
SVPSLGPMPTAAQPSTTGIRKQWHEDITQDLRNHLVHKLVQAIFPTPDPAALKDRRMENLVAYARKVEGD
MYESANNRAEYYHLLAEKIYKIQKELEEKRRTRLQKQNMLPNAAGMVPVSMNPGPNMGQPQPGMTSNG
PLPDPSMIRGSVPNQMMPRITPQSGLNQFGQMSMAQPPIVPRQTPPLQHHGQLAQPGALNPPMGYGPRM
QQPSNQGQFLPQTQFPSQGMNVTNIPLAPSSGQAPVSQAQMSSSSCPVNSPIMPPGSQGSHIHCPQLPQPAL
HQNSPSPVPSRTPTPHHTPPSIGAQQPPATTIPAPVPTPPAMPPGPQSQALHPPPRQTPTPPTTQLPQQVQPSLP
AAPSADQPQQQPRSQQSTAASVPTPNAPLLPPQPATPLSQPAVSIEGQVSNPPSTSSTEVNSQAIAEKQPSQE
VKMEAKMEVDQPEPADTQPEDISESKVEDCKMESTETEERSTELKTEIKEEEDQPSTSATQSSPAPGQSKK
KIFKPEELRQALMPTLEALYRQDPESLPFRQPVDPQLLGIPDYFDIVKSPMDLSTIKRKLDTGQYQEPWQY
VDDIWLMFNNAWLYNRKTSRVYKYCSKLSEVFEQEIDPVMQSLGYCCGRKLEFSPQTLCCYGKQLCTIPR
DATYYSYQNRYHFCEKCFNEIQGESVSLGDDPSQPQTTINKEQFSKRKNDTLDPELFVECTECGRKMHQIC
VLHHEIIWPAGFVCDGCLKKSARTRKENKFSAKRLPSTRLGTFLENRVNDFLRRQNHPESGEVTVRVVHA
SDKTVEVKPGMKARFVDSGEMAESFPYRTKALFAFEEIDGVDLCFFGMHVQEYGSDCPPPNQRRVYISYL
DSVHFFRPKCLRTAVYHEILIGYLEYVKKLGYTTGHIWACPPSEGDDYIFHCHPPDQKIPKPKRLQEWYKK
MLDKAVSERIVHDYKDIFKQATEDRLTSAKELPYFEGDFWPNVLEESIKELEQEEEERKREENTSNESTDV
TKGDSKNAKKKNNKKTSKNKSSLSRGNKKKPGMPNVSNDLSQKLYATMEKHKEVFFVIRLIAGPAANSL
PPIVDPDPLIPCDLMDGRDAFLTLARDKHLEFSSLRRAQWSTMCMLVELHTQSQDRFVYTCNECKHHVET
RWHCTVCEDYDLCITCYNTKNHDHKMEKLGLGLDDESNNQQAAATQSPGDSRRLSIQRCIQSLVHACQC
RNANCSLPSCQKMKRVVQHTKGCKRKTNGGCPICKQLIALCCYHAKHCQENKCPVPFCLNIKQKLRQQQ
LQHRLQQAQMLRRRMASMQRTGVVGQQQGLPSPTPATPTTPTGQQPTTPQTPQPTSQPQPTPPNSMPPYL
PRTQAAGPVSQGKAAGQVTPPTPPQTAQPPLPGPPPTAVEMAMQIQRAAETQRQMAHVQIFQRPIQHQMP
PMTPMAPMGMNPPPMTRGPSGHLEPGMGPTGMQQQPPWSQGGLPQPQQLQSGMPRPAMMSVAQHGQP
LNMAPQPGLGQVGISPLKPGTVSQQALQNLLRTLRSPSSPLQQQQVLSILHANPQLLAAFIKQRAAKYANS
NPQPIPGQPGMPQGQPGLQPPTMPGQQGVHSNPAMQNMNPMQAGVQRAGLPQQQPQQQLQPPMGGMS
PQAQQMNMNHNTMPSQFRDILRRQQMMQQQQQQGAGPGIGPGMANHNQFQQPQGVGYPPQPQQRMQ
HHMQQMQQGNMGQIGQLPQALGAEAGASLQAYQQRLLQQQMGSPVQPNPMSPQQHMLPNQAQSPHLQ
GQQIPNSLSNQVRSPQPVPSPRPQSQPPHSSPSPRMQPQPSPHHVSPQTSSPHPGLVAAQANPMEQGHFASP
DQNSMLSQLASNPGMANLHGASATDLGLSTDNSDLNSNLSQSTLDIH
>T01428
MQLRKMQTVKKEQASLDASSNVDKMMVLNSALTEVSEDSTTGEDVLLSEGSVGKNKSSACRRKREFIPD
EKKDAMYWEKRRKNNEAAKRSREKRRLNDLVLENKLIALGEENATLKAELLSLKLKFGLISSTAYAQEIQ
KLSNSTAVYFQDYQTSKSNVSSFVDEHEPSMVSSSCISVIKHSPQSSLSDVSEVSSVEHTQESSVQGSCRSPE
NKFQIIKQEPMELESYTREPRDDRGSYTASIYQNYMGNSFSGYSHSPPLLQVNRSSSNSPRTSETDDGVVG
KSSDGEDEQQVPKGPIHSPVELKHVHATVVKVPEVNSSALPHKLRIKAKAMQIKVEAFDNEFEATQKLSSP
IDMTSKRHFELEKHSAPSMVHSSLTPFSVQVTNIQDWSLKSEHWHQKELSGKTQNSFKTGVVEMKDSGY
KVSDPENLYLKQGIANLSAEVVSLKRLIATQPISASDSG
>T01468
MAAESDVLHFQFEQQGDVVLQKMNLLRQQNLFCDVSIYINDTEFQGHKVILAACSTFMRDQFLLTQSKH
VRITILQSAEVGRKLLLSCYTGALEVKRKELLKYLTAASYLQMVHIVEKCTEALSKYLEIDLSMKNNNQH
TDLCQSSDPDVKNEDENSDKDCEIIEISEDSPVNIDFHVKEEESNALQSTVESLTSERKEMKSPELSTVDIGF
KDNEICILHVESISTAGVENGQFSQPCTSSKASMYFSETQHSLINSTVESRVAEVPGNQDQGLFCENTEGSY
GTVSEIQNLEEGYSLRHQCPRCPRGFLHVENYLRHLKMHKLFLCLQCGKTFTQKKNLNRHIRGHMGIRPF

QCTVCLKTFTAKSTLQDHLNIHSGDRPYKCHCCDMDFKHKSALKKHLTSVHGRSSGEKLSRPDLKRQSLL
>T01527
MESAPAAPDPAASEPGSSGADAAAGSRETPLNQESARKSEPPAPVRRQSYSSTSRGISVTKKTHTSQIEIIPC
KICGDKSSGIHYGVITCEGCKGFFRRSQQSNATYSCPRQKNCLIDRTSRNRCQHCRLQKCLAVGMSRDAVK
FGRMSKKQRDSLYAEVQKHRMQQQQRDHQQQPGEAEPLTPTYNISANGLTELHDDLSNYIDGHTPEGSK
ADSAVSSFYLDIQPSPDQSGLDINGIKPEPICDYTPASGFFPYCSFTNGETSPTVSMAELEHLAQNISKSHLET
CQYLREELQQITWQTFLQEEIENYQNKQREVMWQLCAIKITEAIQYVVEFAKRIDGFMELCQNDQIVLLK
AGSLEVVFIRMCRAFDSQNNTVYFDGKYASPDVFKSLGCEDFISFVFEFGKSLCSMHLTEDEIALFSAFVL
MSADRSWLQEKVKIEKLQQKIQLALQHVLQKNHREDGILTKLICKVSTLRALCGRHTEKLMAFKAIYPDI
VRLHFPPLYKELFTSEFEPAMQIDG
>T01528
MNEGAPGDSDLETEARVPWSIMGHCLRTGQARMSATPTPAGEGARRDELFGILQILHQCILSSGDAFVLTG
VCCSWRQNGKPPYSQKEDKEVQTGYMNAQIEIIPCKICGDKSSGIHYGVITCEGCKGFFRRSQQSNATYSC
PRQKNCLIDRTSRNRCQHCRLQKCLAVGMSRDAVKFGRMSKKQRDSLYAEVQKHRMQQQQRDHQQQPG
EAEPLTPTYNISANGLTELHDDLSNYIDGHTPEGSKADSAVSSFYLDIQPSPDQSGLDINGIKPEPICDYTPAS
GFFPYCSFTNGETSPTVSMAELEHLAQNISKSHLETCQYLREELQQITWQTFLQEEIENYQNKQREVMWQ
LCAIKITEAIQYVVEFAKRIDGFMELCQNDQIVLLKAGSLEVVFIRMCRAFDSQNNTVYFDGKYASPDVFK
SLGCEDFISFVFEFGKSLCSMHLTEDEIALFSAFVLMSADRSWLQEKVKIEKLQQKIQLALQHVLQKNHRE
DGILTKLICKVSTLRALCGRHTEKLMAFKAIYPDIVRLHFPPLYKELFTSEFEPAMQIDG
>T01553
MLEMLEYNHYQVQTHLENPTKYHIQQAQRQQVKQYLSTTLANKHANQVLSLPCPNQPGDHVMPPVPGS
SAPNSPMAMLTLNSNCEKEGFYKFEEQNRAESECPGMNTHSRASCMQMDDVIDDIISLESSYNEEILGLM
DPALQMANTLPVSGNLIDLYGNQGLPPPGLTISNSCPANLPNIKRELTACIFPTESEARALAKERQKKDNHN
LIERRRRFNINDRIKELGTLIPKSNDPDMRWNKGTILKASVDYIRKLQREQQRAKELENRQKKLEHANRHL
LLRIQELEMQARAHGLSLIPSTGLCSPDLVNRIIKQEPVLENCSQDLLQHHADLTCTTTLDLTDGTITFNNNL
GTGTEANQAYSVPTKMGSKLEDILMDDTLSPVGVTDPLLSSVSPGASKTSSRRSSMSMEETEHTC
>T01673
MATQAYTELQAAPPPSQPPQAPPQAQPQPPPPPPPAAPQPPQPPTAAATPQPQYVTELQSPQPQAQPPGGQK
QYVTELPAVPAPSQPTGAPTPSPAPQQYIVVTVSEGAMRASETVSEASPGSTASQTGVPTQVVQQVQGTQQ
RLLVQTSVQAKPGHVSPLQLTNIQVPQQALPTQRLVVQSAAPGSKGGQVSLTVHGTQQVHSPPEQSPVQA
NSSSSKTAGAPTGTVPQQLQVHGVQQSVPVTQERSVVQATPQAPKPGPVQPLTVQGLQPVHVAQEVQQL
QQVPVPHVYSSQVQYVEGGDASYTASAIRSSTYSYPETPLYTQTASTSYYEAAGTATQVSTPATSQAVASSG
SMPMYVSGSQVVASSASTGAGASNSSGGGGSGGGGGGGGGGGGGSGSTGGGGSGAGTYVIQGGYML
GSASQSYSHTTRASPATVQWLLDNYETAEGVSLPRSTLYCHYLLHCQEQKLEPVNAASFGKLIRSVFMGL
RTRRLGTRGNSKYHYYGLRIKASSPLLRLMEDQQHMAMRGQPFSQKQRLKPIQKMEGMTNGVAVGQQP
STGLSDISAQVQQYQQFLDASRSLPDFTELDLQGKVLPEGVGPGDIKAFQVLYREHCEAIVDVMVNLQFTL
VETLWKTFWRYNLSQPSEAPPLAVHDEAEKRLPKAILVLLSKFEPVLQWTKHCDNVLYQGLVEILIPDVLR
PIPSALTQAIRNFAKSLESWLTHAMVNIPEEMLRVKVAAAGAFAQTLRRYTSLNHLAQAARAVLQNTAQIN
QMLSDLNRVDFANVQEQASWVCRCEDRVVQRLEQDFKVTLQQQNSLEQWAAWLDGVVSQVLKPYQGS
AGFPKAAKLFLLKWSFYSSMVIRDLTLRSAASFGSFHLIRLLYDEYMYYLIEHRVAQAKGETPIAVMGEFA
NLATSLNPLDPDKDEEEEEEEESEDELPQDISLAAGGESPALGPETLEPPAKLARTDARGLFVQALPSS
>T01804
MEQYTANSNSSTEQIVVQAGQIQQQQQGGVTAVQLQTEAQVASASGQQVQTLQVVQGQPLMVQVSGGQ
LITSTGQPIMVQAVPGGQGQTIMQVPVSGTQGLQQIQLVPPGQIQIQGGQAVQVQGQQGQTQQIIIQQPQTA

VTAGQTQTQQQIAVQGQQVAQTAEGQTIVYQPVNADGTILQQVTVPVSGMITIPAASLAGAQIVQTGANT
NTTSSGQGTVTVTLPVAGNVVNSGGMVMMVPGAGSVPAIQRIPLPGAEMLEEEPLYVNAKQYHRILKRR
QARAKLEAEGKIPKERRKYLHESRHRHAMARKRGEGGRFFSPKEKDSPHMQDPNQADEEAMTQIIRVS
>T01853
MNLLDPFMKMTDEQEKGLSGAPSPTMSEDSAGSPCPSGSGSDTENTRPQENTFPKGEPDLKKESEEDKFP
VCIREAVSQVLKGYDWTLVPMPVRVNGSSKNKPHVKRPMNAFMVWAQAARRKLADQYPHLHNAELSK
TLGKLWRLLNESEKRPFVEEAERLRVQHKKDHPDYKYQPRRRKSVKNGQAEAEEATEQTHISPNAIFKAL
QADSPHSSSGMSEVHSPGEHSGQSQGPPTPPTTPKTDVQPGKADLKREGRPLPEGGRQPPIDFRDVDIGEL
SSDVISNIETFDVNEFDQYLPPNGHPGVPATHGQVTYTGSYGISSTAATPASAGHVWMSKQQAPPPPPQQPP
QAPPAPQAPPQPQAAPPQQPAAPPQQPQAHTLTTLSSEPGQSQRTHIKTEQLSPSHYSEQQQHSPQQIAYSPF
NLPHYSPSYPPITRSQYDYTDHQNSSSYYSHAAGQGTGLYSTFTYMNPAQRPMYTPIADTSGVPSIPQTHSP
QHWEQPVYTQLTRP
>T01920
MDSKESLTPGREENPSSVLAQERGDVMDFYKTLRGGATVKVSASSPSLAVASQSDSKQRRLLVDFPKGSV
SNAQQPDLSKAVSLSMGLYMGETETKVMGNDLGFPQQGQISLSSGETDLKLLEESIANLNRSTSVPENPKS
SASTAVSAAPTEKEFPKTHSDVSSEQQHLKGQTGTNGGNVKLYTTDQSTFDILQDLEFSSGSPGKETNESP
WRSDLLIDENCLLSPLAGEDDSFLLEGNSNEDCKPLILPDTKPKIKDNGDLVLSSPSNVTLPQVKTEKEDFI
ELCTPGVIKQEKLGTVYCQASFPGANIIGNKMSAISVHGVSTSGGQMYHYDMNTASLSQQQDQKPIFNVIP
PIPVGSENWNRCQGSGDDNLTSLGTLNFPGRTVFSNGYSSPSMRPDVSSPPSSSSTATTGPPPKLCLVCSDE
ASGCHYGVLTCGSCKVFFKRAVEGQHNYLCAGRNDCIIDKIRRKNCPACRYRKCLQAGMNLEARKTKKK
IKGIQQATTGVSQETSENPGNKTIVPATLPQLTPTLVSLLEVIEPEVLYAGYDSSVPDSTWRIMTTLNMLGGR
QVIAAVKWAKAIPGFRNLHLDDQMTLLQYSWMFLMAFALGWRSYRQSSANLLCFAPDLIINEQRMTLPC
MYDQCKHMLYVSSELHRLQVSYEEYLCMKTLLLLSSVPKDGLKSQELFDEIRMTYIKELGKAIVKREGNS
SQNWQRFYQLTKLLDSMHENVMWLKPESTSHTLI
>T01948
MNAPERQPQPDGGDAPGHEPGGSPQDELDFSILFDYEYLNPNEEEPNAHKVASPPSGPAYPDDVMDYGLK
PYSPLASLSGEPPGRFGEPDRVGPQKFLSAAKPAGASGLSPRIEITPSHELIQAVGPLRMRDAGLLVEQPPLA
GVAASPRFTLPVPGFEGYREPLCLSPASSGSSASFISDTFSPYTSPCVSPNNGGPDDLCPQFQNIPAHYSPRTS
PIMSPRTSLAEDSCLGRHSPVPRPASRSSSPGAKRRHSCAEALVALPPGASPQRSRSPSPQPSSHVAPQDHGS
PAGYPPVAGSAVIMDALNSLATDSPCGIPPKMWKTSPDPSPVSAAPSKAGLPRHIYPAVEFLGPCEQGERRN
SAPESILLVPPTWPKPLVPAIPICSIPVTASLPPLEWPLSSQSGSYELRIEVQPKPHHRAHYETEGSRGAVKAP
TGGHPVVQLHGYMENKPLGLQIFIGTADERILKPHAFYQVHRITGKTVTTTSYEKIVGNTKVLEIPLEPKN
NMRATIDCAGILKLRNADIELRKGETDIGRKNTRVRLVFRVHIPESSGRIVSLQTASNPIECSQRSAHELPMV
ERQDTDSCLVYGGQQMILTGQNFTSESKVVFTEKTTDGQQIWEMEATVDKDKSQPNMLFVEIPEYRNKHI
RTPVKVNFYVINGKRKRSQPQHFTYHPVPAIKTEPTDEYDPTLICSPTHGGLGSQPYYPQHPMVAESPSCLV
ATMAPCQQFRTGLSSPDARYQQQNPAAVLYQRSKSLSPSLLGYQQPALMAAPLSLADAHRSVLVHAGSQG
QSSALLHPSPTNQQASPVIHYSPTNQQLRCGSHQEFQHIMYCENFAPGTTRPGPPPVSQGQRLSPGSYPTVI
QQQNATSQRAAKNGPPVSDQKEVLPAGVTIKQEQNLDQTYLDDVNEIIRKEFSGPPARNQT
>T01950
MVSKLSQLQTELLAALLESGLSKEALIQALGEPGPYLLAGEGPLDKGESCGGGRGELAELPNGLGETRGS
EDETDDDGEDFTPPILKELENLSPEEAAHQKAVVETLLQEDPWRVAKMVKSYLQQHNIPQREVVDTTGLN
QSHLSQHLNKGTPMKTQKRAALYTWYVRKQREVAQQFTHAGQGGLIEEPTGDELPTKKGRRNRFKWGP
ASQQILFQAYERQKNPSKEERETLVEECNRAECIQRGVSPSQAQGLGSNLVTEVRVYNWFANRRKEEAFR
HKLAMDTYSGPPPGPGPGPALPAHSSPGLPPPALSPSKVHGVRYGQPATSETAEVPSSSGGPLVTVSTPLHQ

VSPTGLEPSHSLLSTEAKLVSAAGGPLPPVSTLTALHSLEQTSPGLNQQPQNLIMASLPGVMTIGPGEPASL
GPTFTNTGASTLVIGLASTQAQSVPVINSMGSSLTTLQPVQFSQPLHPSYQQPLMPPVQSHVTQSPFMATM
AQLQSPHGEHPVPHTAGDDDRGWLSMDAGERGAWQALQSACVSGTSVFP
>T01951
MVSKLSQLQTELLAALLESGLSKEALIQALGEPGPYLLAGEGPLDKGESCGGGRGELAELPNGLGETRGS
EDETDDDGEDFTPPILKELENLSPEEAAHQKAVVETLLQEDPWRVAKMVKSYLQQHNIPQREVVDTTGLN
QSHLSQHLNKGTPMKTQKRAALYTWYVRKQREVAQQFTHAGQGGLIEEPTGDELPTKKGRRNRFKWGP
ASQQILFQAYERQKNPSKEERETLVEECNRAECIQRGVSPSQAQGLGSNLVTEVRVYNWFANRRKEEAFR
HKLAMDTYSGPPPGPGPGPALPAHSSPGLPPPALSPSKVHGVRYGQPATSETAEVPSSSGGPLVTVSTPLHQ
VSPTGLEPSHSLLSTEAKLVSAAGGPLPPVSTLTALHSLEQTSPGLNQQPQNLIMASLPGVMTIGPGEPASL
GPTFTNTGASTLVIGKLVGMGGHLGGRLMGQPQNPGAGRATGTHSFIHSFIQHVFIQCLLWTSHCATSVIP
G
>T02513
MLGAVKMEGHEPSDWSSYYAEPEGYSSVSNMNAGLGMNGMNTYMSMSAAAMGSGSGNMSAGSMNM
SSYVGAGMSPSLAGMSPGAGAMAGMGGSAGAAGVAGMGPHLSPSLSPLGGQAAGAMGGLAPYANMNS
MSPMYGQAGLSRARDPKTYRRSYTHAKPPYSYISLITMAIQQSPNKMLTLSEIYQWIMDLFPFYRQNQQR
WQNSIRHSLSFNDCFLKVPRSPDKPGKGSFWTLHPDSGNMFENGCYLRRQKRFKCEKQLALKEAAGAAG
SGKKAAAGAQASQAQLGEAAGPASETPAGTESPHSSASPCQEHKRGGLGELKGTPAAALSPPEPAPSPGQ
QQQAAAHLLGPPHHPGLPPEAHLKPEHHYAFNHPFSINNLMSSEQQHHHSHHHHQPHKMDLKAYEQVM
HYPGYGSPMPGSLAMGPVTNKTGLDASPLAADTSYYQGVYSRPIMNSS
>T02689
MYQTLAALSSQGPAAYDGAPGGFVHSAAAAAAAAAAASSPVYVPTTRVGSMLPGLPYHLQGSGSGPAN
HAGGAGAHPGWPQASADSPPYGSGGGAAGGGAAGPGGAGSAAAHVSARFPYSPSPPMANGAAREPGG
YAAAGSGGAGGVSGGGSSLAAMGGREPQYSSLSAARPLNGTYHHHHHHHHHPSPYSPYVGAPLTPAWP
AGPFETPVLHSLQSRAGAPLPVPRGPSADLLEDLSESRECVNCGSIQTPLWRRDGTGHYLCNACGLYSKM
NGLSRPLIKPQKRVPSSRRLGLSCANCHTTTTTLWRRNAEGEPVCNACGLYMKLHGVPRPLAMKKEGIQT
RKRKPKNINKSKTCSGNSNNSIPMTPTSTSSNSDDCSKNTSPTTQPTASGAGAPVMTGAGESTNPENSELK
YSGQDGLYIGVSLASPAEVTSSVRPDSWCALALA
>T03978
MEFLSEKFALKSPPSKNSDFYMGAGGPLEHVMETLDNESFYSKASAGKCVQAFGPLPRAEHHVRLERTSP
CQDSSVNYGITKVEGQPLHTELNRAMDNCNSLRMSPVKGMQEKGELDELGDKCDTNVSSSKKRRHRTTF
TSLQLEELEKVFQKTHYPDVYVREQLALRTELTEARVQVWFQNRRAKWRKRERYGQIQQAKSHFAATYD
ISVLPRTDSYPQIQNNLWAGNASGGSVVTSCMLPRDTSSCMTPYSHSPRTDSSYTGFSNHQNQFSHVPLNN
FFTDSLLTGATNGHAFETKPEFERRSSSIAVLRMKAKEHTANISWAM
>T04139
MTGKAGEALSKPKSETVAKSTSGGAPARCTGFGIQEILGLNKEPPSSHPRAALDGLAPGHLLAARSVLSPA
GVGGMGLLGPGGLPGFYTQPTFLEVLSDPQSVHLQPLGRASGPLDTSQTASSDSEDVSSSDRKMSKSALN
QTKKRKKRRHRTIFTSYQLEELEKAFNEAHYPDVYAREMLAMKTELPEDRIQVWFQNRRAKWRKREKC
WGRSSVMAEYGLYGAMVRHSIPLPESILKSAKDGIMDSCAPWLLGMHKKSLEAAAESGRKPEGERQALP
KLDKMEQDERGPDAQAAISQEELRENSIAVLRAKAQEHSTKVLGTVSGPDSLARSTEKPEEEEAMDEDRP
AERLSPPQLEDMA
>T04255
MLRVPEPRPGEAKAEGAAPPTPSKPLTSFLIQDILRDGAQRQGGRTSSQRQRDPEPEPEPEPEGGRSRAGAQ
NDQLSTGPRAAPEEAETLAETEPERHLGSYLLDSENTSGALPRLPQTPKQPQKRSRAAFSHTQVIELERKFS

HQKYLSAPERAHLAKNLKLTETQVKIWFQNRRYKTKRKQLSSELGDLEKHSSLPALKEEAFSRASLVSVY
NSYPYYPYLYCVGSWSPAFG
>T04345
MADADEGFGLAHTPLEPDAKDLPCDSKPESALGAPSKSPSSPQAAFTQQGMEGIKVFLHERELWLKFHEV
GTEMIITKAGRRMFPSYKVKVTGLNPKTKYILLMDIVPADDHRYKFADNKWSVTGKAEPAMPGRLYVHP
DSPATGAHWMRQLVSFQKLKLTNNHLDPFGHIILNSMHKYQPRLHIVKADENNGFGSKNTAFCTHVFPET
AFIAVTSYQNHKITQLKIENNPFAKGFRGSDDMELHRMSRMQSKEYPVVPRSTVRQKVASNHSPFSSESRA
LSTSSNLGSQYQCENGVSGPSQDLLPPPNPYPLPQEHSQIYHCTKRKEEECSTTDHPYKKPYMETSPSEEDS
FYRSSYPQQQGLGASYRTESAQRQACMYASSAPPSEPVPSLEDISCNTWPSMPSYSSCTVTTVQPMDRLPY
QHFSAHFTSGPLVPRLAGMANHGSPQLGEGMFQHQTSVAHQPVVRQCGPQTGLQSPGTLQPPEFLYSHGV
PRTLSPHQYHSVHGVGMVPEWSDNS
>T04651
MDIKNSPSSLNSPSSYNCSQSILPLEHGSIYIPSSYVDSHHEYPAMTFYSPAVMNYSIPSNVTNLEGGPGRQT
TSPNVLWPTPGHLSPLVVHRQLSHLYAEPQKSPWCEARSLEHTLPVNRETLKRKVSGNRCASPVTGPGSK
RDAHFCAVCSDYASGYHYGVWSCEGCKAFFKRSIQGHNDYICPATNQCTIDKNRRKSCQACRLRKCYEV
GMVKCGSRRERCGYRLVRRQRSADEQLHCAGKAKRSGGHAPRVRELLLDALSPEQLVLTLLEAEPPHVLI
SRPSAPFTEASMMMSLTKLADKELVHMISWAKKIPGFVELSLFDQVRLLESCWMEVLMMGLMWRSIDHP
GKLIFAPDLVLDRDEGKCVEGILEIFDMLLATTSRFRELKLQHKEYLCVKAMILLNSSMYPLVTATQDADSS
RKLAHLLNAVTDALVWVIAKSGISSQQQSMRLANLLMLLSHVRHASNKGMEHLLNMKCKNVVPVYDLL
LEMLNAHVLRGCKSSITGSECSPAEDSKSKEGSQNPQSQ
>T04682
MVSEEEEEEDGDAEETQDSEDDEEDEMEEDDDDSDYPEEMEDDDDDASYCTESSFRSHSTYSSTPGRRKP
RVHRPRSPILEEKDIPPLEFPKSSEDLMVPNEHIMNVIAIYEVLRNFGTVLRLSPFRFEDFCAALVSQEQCTL
MAEMHVVLLKAVLREEDTSNTTFGPADLKDSVNSTLYFIDGMTWPEVLRVYCESDKEYHHVLPYQEAED
YPYGPVENKIKVLQFLVDQFLTTNIAREELMSEGVIQYDDHCRVCHKLGDLLCCETCSAVYHLECVKPPLE
EVPEDEWQCEVCVAHKVPGVTDCVAEIQKNKPYIRHEPIGYDRSRRKYWFLNRRLIIEEDTENENEKKIW
YYSTKVQLAELIDCLDKDYWEAELCKILEEMREEIHRHMDITEDLTNKARGSNKSFLAAANEEILESIRAK
KGDIDNVKSPEETEKDKNETENDSKDAEKNREEFEDQSLEKDSDDKTPDDDPEQGKSEEPTEVGDKGNS
VSANLGDNTTNATSEETSPSEGRSPVGCLSETPDSSNMAEKKVASELPQDVPEEPNKTCESSNTSATTTSIQ
PNLENSNSSSELNSSQSESAKAADDPENGERESHTPVSIQEEIVGDFTSEKSTGELSESPGAGKGASGSTRII
TRLRNPDSKLSQLKSQQVAAAAHEANKLFKEGKEVLVVNSQGEISRLSTKKEVIMKGNINNYFKLGQEGK
YRVYHNQYSTNSFALNKHQHREDHDKRRHLAHKFCLTPAGEFKWNGSVHGSKVLTISTLRLTITQLETTSL
HPSFIPTGHHIGQIGSRQFRCVANPENLHWL
>T04683
MAGWIQAQQLQGDALRQMQVLYGQHFPIEVRHYLAQWIESQPWDAIDLDNPQDRAQATQLLEGLVQEL
QKKAEHQVGEDGFLLKIKLGHYATQLQKTYDRCPLELVRCIRHILYNEQRLVREANNCSSPAGILVDAMSQ
KHLQINQTFEELRLVTQDTENELKKLQQTQEYFIIQYQESLRIQAQFAQLAQLSPQERLSRETALQQKQVSL
EAWLQREAQTLQQYRVELAEKHQKTLQLLRKQQTIILDDELIQWKRRQQLAGNGGPPEGSLDVLQSWCE
KLAEIIWQNRQQIRRAEHLCQQLPIPGPVEEMLAEVNATITDIISALVTSTFIIEKQPPQVLKTQTKFAATVRL
LVGGKLNVHMNPPQVKATIISEQQAKSLLKNENTRNECSGEILNNCCVMEYHQATGTLSAHFRNMSLKRI
KRADRRGAESVTEEKFTVLFESQFSVGSNELVFQVKTLSLPVVVIVHGSQDHNATATVLWDNAFAEPGRV
PFAVPDKVLWPQLCEALNMKFKAEVQSNRGLTKENLVFLAQKLFNNSSSHLEDYSGLSVSWSQFNRENLP
GWNYTFWQWFDGVMEVLKKHHKPHWNDGAILGFVNKQQAHDLLINKPDGTFLLRFSDSEIGGITIAWKF
DSPERNLWNLKPFTTRDFSIRSLADRLGDLSYLIYVFPDRPKDEVFSKYYTPVLAKAVDGYVKPQIKQVVP

EFVNASADAGGSSATYMDQAPSPAVCPQAPYNMYPQNPDHVLDQDGEFDLDETMDVARHVEELLRRPM
DSLDSRLSPPAGLFTSARGSLS
>T04684
MAVWIQAQQLQGEALHQMQALYGQHFPIEVRHYLSQWIESQAWDSVDLDNPQENIKATQLLEGLVQELQ
KKAEHQVGEDGFLLKIKLGHYATQLQNTYDRCPMELVRCIRHILYNEQRLVREANNGSSPAGSLADAMSQ
KHLQINQTFEELRLVTQDTENELKKLQQTQEYFIIQYQESLRIQAQFGPLAQLSPQERLSRETALQQKQVSL
EAWLQREAQTLQQYRVELAEKHQKTLQLLRKQQTIILDDELIQWKRRQQLAGNGGPPEGSLDVLQSWCE
KLAEIIWQNRQQIRRAEHLCQQLPIPGPVEEMLAEVNATITDIISALVTSTFIIEKQPPQVLKTQTKFAATVRL
LVGGKLNVHMNPPQVKATIISEQQAKSLLKNENTRNDYSGEILNNCCVMEYHQATGTLSAHFRNMSLKRI
KRSDRRGAESVTEEKFTILFESQFSVGGNELVFQVKTLSLPVVVIVHGSQDNNATATVLWDNAFAEPGRVP
FAVPDKVLWPQLCEALNMKFKAEVQSNRGLTKENLVFLAQKLFNNSSSHLEDYSGLSVSWSQFNRENLPG
RNYTFWQWFDGVMEVLKKHLKPHWNDGAILGFVNKQQAHDLLINKPDGTFLLRFSDSEIGGISIAWKFD
SQERMFWNLMPFTTRDFSIRSLADRLGDLNYLIYVFPDRPKDEVYSKYYTPVPCESATAKAVDGYVKPQI
KQVVPEFVNASADGGRSATYMDQAPSPAVCPQAHYNMYPQNPDSVLDTDGDFDLEDTMDVARRVEELL
GRPMDSQWIPHAQS
>T05040
MEFPDHSRHLLQCLSEQRHQGFLCDCTVLVGDAQFRAHRAVLASCSMYFHLFYKDQLDKRDIVHLNSDI
VTAPAFALLLEFMYEGKLQFKDLPIEDVLAAASYLHMYDIVKVCKKKLKEKATTEADSTKKEEDASSCSD
KVESLSDGSSHIAGDLPSDEDEGEDEKLNILPSKRDLAAEPGNMWMRLPSDSAGIPQAGGEAEPHATAAG
KTVASPCSSTESLSQRSVTSVRDSADVDCVLDLSVKSSLSGVENLNSSYFSSQDVLRSNLVQVKVEKEASC
DESDVGTNDYDMEHSTVKESVSTNNRVQYEPAHLAPLREDSVLRELDREDKASDDEMMTPESERVQVEG
GMESSLLPYVSNILSPAGQIFMCPLCNKVFPSPHILQIHLSTHFREQDGIRSKPAADVNVPTCSLCGKTFSCM
YTLKRHERTHSGEKPYTCTQCGKSFQYSHNLSRHAVVHTREKPHACKWCERRFTQSGDLYRHIRKFHCEL
VNSLSVKSEALSLPTVRDWTLEDSSQELWK
>T06429
MLDTMEAPGHSRQLLLQLNNQRTKGFLCDVIIVVQNALFRAHKNVLAASSAYLKSLVVHDNLLNLDHD
MVSPAVFRLVLDFIYTGRLADGAEAAAAAAVAPGAEPSLGAVLAAASYLQIPDLVALCKKRLKRHGKYCH
LRGGGGGGGGYAPYGRPGRGLRAATPVIQACYRSPVGPPPPPAAEPPSGPEAAVNTHCAELYASGPGPAAA
LCASERRCSPLCGLDLSKKSPPGSAAPERPLAERELPPRPDSPPSAGPAAYKEPPLALPSLPPLPFQKLEEAA
PPSDPFRGGSGSPGPEPPGRPDGPSLLYRWMKHEPGLGSYGDELGRERGSPSERCEERGGDAAVSPGGPPL
GLAPPPRYPGSLDGPGAGGDGDDYKSSSEETGSSEDPSPPGGHLEGYPCPHLAYGEPESFGDNLYVCIPCG
KGFPSSEQLNAHVEAHVEEEEALYGRAEAAEVAAGAAGLGPPFGGGGDKVAGAPGGLGELLRPYRCASC
DKSYKDPATLRQHEKTHWLTRPYPCTICGKKFTQRGTMTRHMRSHLGLKPFACDACGMRFTRQYRLTEH
MRIHSGEKPYECQVCGGKFAQQRNLISHMKMHAVGGAAGAAGALAGLGGLPGVPGPDGKGKLDFPEGV
FAVARLTAEQLSLKQQDKAAAAELLAQTTHFLHDPKVALESLYPLAKFTAELGLSPDKAAEVLSQGAHLA
AGPDGGTIDRFSPT
>T08292
MEFPGLGSLGTSEPLPQFVDPALVSSTPESGVFFPSGPEGLDAAASSTAPSTATAAAAALAYYRDAEAYRHS
PVFQVYPLLNCMEGIPGGSPYAGWAYGKTGLYPASTVCPTREDSPPQAVEDLDGKGSTSFLETLKTERLSP
DLLTLGPALPSSLPVPNSAYGGPDFSSTFFSPTGSPLNSAAYSSPKLRGTLPLPPCEARECVNCGATATPLWR
RDRTGHYLCNACGLYHKMNGQNRPLIRPKKRLIVSKRAGTQCTNCQTTTTTLWRRNASGDPVCNACGLY
YKLHQVNRPLTMRKDGIQTRNRKASGKGKKKRGSSLGGTGAAEGPAGGFMVVAGGSGSGNCGEVASGL
TLGPPGTAHLYQGLGPVVLSGPVSHLMPFPGPLLGSPTGSFPTGPMPPTTSTTVVAPLSS
>T08300

MTMTLHTKASGMALLHQIQGNELEPLNRPQLKIPLERPLGEVYLDSSKPAVYNYPEGAAYEFNAAAAAN
AQVYGQTGLPYGPGSEAAAFGSNGLGGFPPLNSVSPSPLMLLHPPPQLSPFLQPHGQQVPYYLENEPSGYT
VREAGPPAFYRPNSDNRRQGGRERLASTNDKGSMAMESAKETRYCAVCNDYASGYHYGVWSCEGCKAF
FKRSIQGHNDYMCPATNQCTIDKNRRKSCQACRLRKCYEVGMMKGGIRKDRRGGRMLKHKRQRDDGE
GRGEVGSAGDMRAANLWPSPLMIKRSKKNSLALSLTADQMVSALLDAEPPILYSEYDPTRPFSEASMMGL
LTNLADRELVHMINWAKRVPGFVDLTLHDQVHLLECAWLEILMIGLVWRSMEHPGKLLFAPNLLLDRNQ
GKCVEGMVEIFDMLLATSSRFRMMNLQGEEFVCLKSIILLNSGVYTFLSSTLKSLEEKDHIHRVLDKITDTL
IHLMAKAGLTLQQQHQRLAQLLLILSHIRHMSNKGMEHLYSMKCKNVVPLYDLLLEMLDAHRLHAPTSR
GGASVEETDQSHLATAGSTSSHSLQKYYITGEAEGFPATV
>T00104
MESADFYEVEPRPPMSSHLQSPPHAPSNARLWLSPGRGPRAAPSPTCRPGAAGRICEHETSIDISAYIDPAAF
NDEFLADLFQHSRQQEKAKAAAGPAGGGGDFDYPGAPAGPGGAVMSAGAHGPPPGYGCAAAGYLDGRL
EPLYERVGAPALRPLVIKQEPREEDEAKQLALAGLFPYQPPPPPPPPHPHASPAHLAAPHLQFQIAHCGQTT
MHLQPGHPTPPPTPVPSPHAAPALGAAGLPGPGSALKGLAGAHPDLRTGGGGGGSGAGAGKAKKSVDKN
SNEYRVRRERNNIAVRKSRDKAKQRNVETQQKVLELTSDNDRLRKRVEQLSRELDTLRGIFRQLPESSLVK
AMGNCA
>T00378
MQKATYYDSSAIYGGYPYQAANGFAYNASQQPYAPSAALGTDGVEYHRPACSLQSPASAGGHPKTHELS
EACLRTLSGPPSQPPGLGEPPLPPPPPQAAPPAPQPPQPPPQPPAPTPAAPPPPSSVSPPQSANSNPTPASTAKS
PLLNSPTVGKQIFPWMKESRQNTKQKTSGSSSGESCAGDKSPPGQASSKRARTAYTSAQLVELEKEFHFNR
YLCRPRRVEMANLLNLTERQIKIWFQNRRMKYKKDQKGKGMLTSSGGQSPSRSPVPPGAGGYLNSMHSL
VNSVPYEPQSPPPFSKPPQGAYGLPPASYPAPLPSCAPPPPPQKRYTAAGSGAGGTPDYDPHAHGLQGNGS
YGTPHLQGSPVFVGGSYVEPMSNSGPLFGLTHLPHTTSAAMDYGGTGPLGSGHHHGPGPGEPHPTYTDLT
AHHPSQGRIQEAPKLTHL
>T00644
MNNPSETNKSSMESEDASTGTQTNGLDFQKQPVPVGGAISTAQAQAFLGHLHQVQLAGTSLQAAAQSLN
VQSKSSEESGDSQQSSQPSSQPPSVQSAIPQTQLMLAGGQITGLTLTPAQQQLLLQQAQAQAQLLAAAVQQ
HSASQQHSAAGATISASAATPMTQIPLSQPIQIAQDLQQLQQLQQQNLNLQQFVLVHPTTNLQPAQFIISQTP
QGQQGLLQAQNLLTQLPQQSQANLLQPQPSITLTSQPTTPTRTIAAASVQTLPQSQSTPKRIDTPSLEEPSDL
EELEQFAKTFKQRRIKLGFTQGDVGLAMGKLYGNDFSQTTISRFEALNLSFKNMCKLKPLLEKWLNDAEN
LSSDSTASSPSALNSPGLGAEGLNRRRKKRTSIETNIRVALEKSFMENQKPTSEDITLIAEQLNMEKEVIRVW
FCNRRQKEKRINPPSSGGTSSSPIKAIFPSPASLVATTPSLVTSSTATTLTVNPVLPLTSAAVTNLSLTDQDLRR
GCSWEVLRSLPDRVTTTAGTTDSTSNNNTATVISTAPPASSAVTSPSLSPSPSASASTSEASSASETNTTQTTS
TPLPSPLGASQVMVTTPGLQTAAAALQGAGQLPANASLAAMAAAAGLSPGLMAPSQFAAGGALLSLSPG
TLGSALSPALMSNSTLATIQALASSGSLPITSLDATGNLVFANAGGAPNIVTAPLFLNPQNLSLLTSNPVSLVS
AAAASTGNSAPTASLHASSTSTESIQSSLFTVASASGPASTTTAASKAQ
>T00648
MVHSSMGAPEIRMSKPLEAEKQSLDSPSEHTDTERNGPDINHQNPQNKASPFSVSPTGPSTKIKAEDPSGD
SAPAAPPPPQPAQPHLPQAQLMLTGSQLAGDIQQLLQLQQLVLVPGHHLQPPAQFLLPQAQQSQPGLLPTP
NLFQLPQQTQGALLTSQPRAGLPTQAMTRPTLPDPHLSHPQPPKCLEPPSHPEEPSDLEELEQFARTFKQRR
IKLGFTQGDVGLAMGKLYGNDFSQTTISRFEALNLSFKNMCKLKPLLEKWLNDAETMSVDSSLPSPNQLS
SPSLGFDGLPGRRRKKRTSIETNVRFALEKSFLANQKPTSEEILLIAEQLHMEKEVIRVWFCNRRQKEKRIN
PCSAAPMLPSPGKPTSYSPHLVTPQGGAGTLPLSQASSSLSTTVTTLSSAVGTLHPSRTAGGGGGGGGRALP
LNSIPSVTPPPPATTNSTNPSPQGSHSAIGLSGLNPSAGPGLWWNPAPYQP

>T00651
MAGHLASDFAFSPPPGGGDGSAGLEPGWVDPRTWLSFQGPPGGPGIGPGSEVLGISPCPPAYEFCGGMAYC
GPQVGLGLVPQVGVETLQPEGQAGARVESNSEGTSSEPCADRPNAVKLEKVEPTPEESQDMKALQKELEQ
FAKLLKQKRITLGYTQADVGLTLGVLFGKVFSQTTICRFEALQLSLKNMCKLRPLLEKWVEEADNNENLQ
EICKSETLVQARKRKRTSIENRVRWSLETMFLKCPKPSLQQITHIANQLGLEKDVVRVWFCNRRQKGKRSS
IEYSQREEYEATGTPFPGGAVSFPLPPGPHFGTPGYGSPHFTTLYSVPFPEGEAFPSVPVTALGSPMHSN
>T00859
MSMSPKHTTPFSVSDILSPLEESYKKVGMEGGGLGAPLAAYRQGQAAPPAAAMQQHAVGHHGAVTAAY
HMTAAGVPQLSHSAVGGYCNGNLGNMSELPPYQDTMRNSASGPGWYGANPDPRFPAISRFMGPASGMN
MSGMGGLGSLGDVSKNMAPLPSAPRRKRRVLFSQAQVYELERRFKQQKYLSAPEREHLASMIHLTPTQV
KIWFQNHRYKMKRQAKDKAAQQQLQQDSGGGGGGGGGAGCPQQQQAQQQSPRRVAVPVLVKDGKPC
QAGAPAPGAASLQSHAQQQAQQQAQAAQAAAAAISVGSGGAGLGAHPGHQPGSAGQSPDLAHHAASPA
GLQGQVSSLSHLNSSGSDYGAMSCSTLLYGRTW
>T01332
MPPPPLGSPFPVISSSMGSPGLPPPAPPGFSGPVSSPQINSTVSLPGGGSGPPEDVKPPVLGVRGLHCPPPPGG
PGAGKRLCAICGDRSSGKHYGVYSCEGCKGFFKRTIRKDLTYSCRDNKDCTVDKRQRNRCQYCRYQKCL
ATGMKREAVQEERQRGKDKDGDGDGAGGAPEEMPVDRILEAELAVEQKSDQGVEGPGATGGGGSSPND
PVTNICQAADKQLFTLVEWAKRIPHFSSLPLDDQVILLRAGWNELLIASFSHRSIDVRDGILLATGLHVHRN
SAHSAGVGAIFDRVLTELVSKMRDMRMDKTELGCLRAIILFNPDAKGLSNPGEVEILREKVYASLETYCKQ
KYPEQQGRFAKLLLRLPALRSIGLKCLEHLFFFKLIGDTPIDTFLMEMLEAPHQLA
>T01483
MDSAAAAFALDPPAPGPGPPPAPGDCAQARKNFSVSHLLDLEEVAAAGRRAAGPVSGPAEARVGAAREPS
GGSSGTEAAPQDGDCPSPGRGTKRKKKQRRNRTTFNSSQLQALERVFERTHYPDAFVREELARRVNLSEA
RVQVWFQNRRAKFRRNERAMLATRSASLLKSYGQEAAIEQPVAPRPTTMSPDYLSWPASSPYSSVPPYSP
GGSSPATPGVNMANSIASLRLKAKEFSLHHSQVPTVN
>T01526
MSSPGTESAGKSLQYRVDHLLSAVESELQAGSEKGDPTERELRVGLEESELWLRFKELTNEMIVTKNGRR
MFPVLKVNVSGLDPNAMYSFLLDFVTADNHRWKYVNGEWVPGGKPEPQAPSCVYIHPDSPNFGAHWM
KAPVSFSKVKLTNKLNGGGQIMLNSLHKYEPRIHIVRVGGPQRMITSHCFPETQFIAVTAYQNEEITALKIK
YNPFAKAFLDAKERNDHKDVMEEPGDCQQPGYSQWGWLVPGAGTLCPPASSHPQFGGSLSLPSTHGCER
YPALRNHRSSPYPSPYAHRNSSPTYADNSSACLSMLQSHDNWSSLGVPGHTSMLPVSHNASPPTGSSQYPS
LWSVSNGTITPGSQTAGVSNGLGAQFFRGSPAHYTPLTHTVSAATSSSSGSPMYEGAATVTDISDSQYDTA
QSLLIASWTPVSPPSM
>T01649
MPADIMEKNSSSPVAATPASVNTTPDKPKTASEHRKSSKPIMEKRRRARINESLSQLKTLILDALKKDSSRH
SKLEKADILEMTVKHLRNLQRAQMTAALSTDPSVLGKYRAGFSECMNEVTRFLSTCEGVNTEVRTRLLG
HLANCMTQINAMTYPGQAHPALQAPPPPPPSGPAGPQHAPFAPPPPPLVPIPGGAAPPPGSAPCKLGSQAGE
AAKVFGGFQVVPAPDGQFAFLIPNGAFAHSGPVIPVYTSNSGTSVGPNAVSPSSGSSLTSDSMWRPWRN
>T01862
MNNPSETNKSSMESEDASTGTQTNGLDFQKQPVPVGGAISTAQAQAFLGHLHQVQLAGTSLQAAAQSLN
VQSKSSEESGDSQQSSQPSSQPPSVQSAIPQTQLMLAGGQITGLTLTPAQQQLLLQQAQAQAQLLAAAVQQ
HSASQQHSAAGATISASAATPMTQIPLSQPIQIAQDLQQLQQLQQQNLNLQQFVLVHPTTNLQPAQFIISQTP
QGQQGLLQAQNLLTQLPQQSQANLLQPQPSITLTSQPTTPTRTIAAASVQTLPQSQSTPKRIDTPSLEEPSDL
EELEQFAKTFKQRRIKLGFTQGDVGLAMGKLYGNDFSQTTISRFEALNLSFKNMCKLKPLLEKWLNDAEN

LSSDSTASSPSALNSPGLGAEGLNRRRKKRTSIETNIRVALEKSFMENQKPTSEDITLIAEQLNMEKEVIRVW
FCNRRQKEKRINPPSSGGTSSSPIKAIFPSPASLVATTPSLVTSSTATTLTVNPVLPLTSAAVTNLSLTGTTDSTS
NNNTATVISTAPPASSAVTSPSLSPSPSASASTSEASSASETNTTQTTSTPLPSPLGASQVMVTTPGLQTAAAA
LQGAGQLPANASLAAMAAAAGLSPGLMAPSQFAAGGALLSLSPGTLGSALSPALMSNSTLATIQALASSG
SLPITSLDATGNLVFANAGGAPNIVTAPLFLNPQNLSLLTSNPVSLVSAAAASTGNSAPTASLHASSTSTESIQ
SSLFTVASASGPASTTTAASKAQ
>T01863
MNNPSETNKSSMESEDASTGTQTNGLDFQKQPVPVGGAISTAQAQAFLGHLHQVQLAGTSLQAAAQSLN
VQSKSSEESGDSQQSSQPSSQPPSVQSAIPQTQLMLAGGQITGLTLTPAQQQLLLQQAQAQAQLLAAAVQQ
HSASQQHSAAGATISASAATPMTQIPLSQPIQIAQDLQQLQQLQQQNLNLQQFVLVHPTTNLQPAQFIISQTP
QGQQGLLQAQNLLTQLPQQSQANLLQPQPSITLTSQPTTPTRTIAAASVQTLPQSQSTPKRIDTPSLEEPSDL
EELEQFAKTFKQRRIKLGFTQGDVGLAMGKLYGNDFSQTTISRFEALNLSFKNMCKLKPLLEKWLNDAEN
LSSDSTASSPSALNSPGLGAEGLNRRRKKRTSIETNIRVALEKSFMENQKPTSEDITLIAEQLNMEKEVIRVW
FCNRRQKEKRINPPSSGGTSSSPIKAIFPSPASLVATTPSLVTSSTATTLTVNPVLPLTSAAVTNLSLTGTTDSTS
NNNTATVISTAPPASSAVTSPSLSPSPSASASTSEASSASETNTTQTTSTPLPSPLGASQVMVTTPGLQTAAAA
LQGAGQLPANASLAAMAAAAGLSPGLMAPSQFAAGGALLSLSPGTLGSALSPALMSNSTLATIQALASSG
SLPITSLDATGNLVFANAGGAPNIVTAPLFLNPQNLSLLTSNPDCFMDWRTF
>T01944
MDVPEPQPDPDGGDGPGHEPGGSPQDELDFSILFDYDYLNPIEEEPIAHKAISSPSGLAYPDDVLDYGLKPC
NPLASLSGEPPGRFGEPDSIGFQNFLSPVKPAGASGPSPRIEITPSHELMQAGGALRGRDAGLSPEQPALALA
GVAASPRFTLPVPGYEGYREPLCLSPASSGSSASFISDTFSPYTSPCVSPNNAGPDDLCPQFQNIPAHYSPRTS
PIMSPRTSLAEDSCLGRHSPVPRPASRSSSPGAKRRHSCAEALVAPLPAASPQRSRSPSPQPSPHVAPQDDSIP
AGYPPTAGSAVLMDALNTLATDSPCGIPSKIWKTSPDPTPVSTAPSKAGLARHIYPTVEFLGPCEQEERRNS
APESILLVPPTWPKQLVPAIPICSIPVTASLPPLEWPLSNQSGSYELRIEVQPKPHHRAHYETEGSRGAVKAPT
GGHPVVQLHGYMENKPLGLQIFIGTADERILKPHAFYQVHRITGKTVTTTSYEKIVGNTKVLEIPLEPKNN
MRATIDCAGILKLRNADIELRKGETDIGRKNTRVRLVFRVHVPEPSGRIVSLQAASNPIECSQRSAHELPMV
ERQDMDSCLVYGGQQMILTGQNFTAESKVVFMEKTTDGQQIWEMEATVDKDKSQPNMLFVEIPEYRNK
HIRVPVKVNFYVINGKRKRSQPQHFTYHPVPAIKTEPSDEYEPSLICSPAHGGLGSQPYYPQHPMLAESPSC
LVATMAPCQQFRSGLSSPDARYQQQSPAAALYQRSKSLSPGLLGYQQPSLLAAPLGLADAHRSVLVHAGS
QGQGQGSTLRHTSSASQQASPVIHYSPTNQQLRGGGHQEFQHIMYCENFGPSSARPGPPPINQGQRLSPGA
YPTVIQQQTAPSQRAAKNGPSDQKEALPTGVTVKQEQNLDQTYLDDAATSESWVGTERYIERKFWKKTL
VQPGLLPSFLLLGSLSAGPRSQTPSERKPIEEDVPLSCSQIAWCCQHPLGTCPVLPGPLAVEWWEGQLGRG
LEPIPWAPDSAGSLHEVDSVGLAGVVGMVLLTLMHHFSMDQNQTPSPHWQRHKEVASPGWI
>T02057
MNSEEQYYAATQLYKDPCAFQRGPVPEFSANPPACLYMGRQPPPPPPPQFTSSLGSLEQGSPPDISPYEVPPL
ASDDPAGAHLHHHLPAQLGLAHPPPGPFPNGTEPGGLEEPNRVQLPFPWMKSTKAHAWKGQWAGGAYT
AEPEENKRTRTAYTRAQLLELEKEFLFNKYISRPRRVELAVMLNLTERHIKIWFQNRRMKWKKEEDKKRSS
GTPSGGGGGEEPEQDCAVTSGEELLAVPPLPPPGGAVPPGVPAAVREGLLPSGLSVSPQPSSIAPLRPQEPR
>T02072
MTSLPLGVKVEDSAFAKPAGGGVGQAPGAAAATATAMGTDEEGAKPKVPASLLPFSVEALMADHRKPGA
KESVLVASEGAQAAGGSVQHLGTRPGSLGAPDAPSSPRPLGHFSVGGLLKLPEDALVKAESPEKLDRTPW
MQSPRFSPPPARRLSPPACTLRKHKTNRKPRTPFTTAQLLALERKFRQKQYLSIAERAEFSSSLSLTETQVKI
WFQNRRAKAKRLQEAELEKLKMAAKPMLPPAAFGLSFPLGGPAAAGASLYSASGPFQRAALPVAPVGLY
TAHVGYSMYHLT

>T02142
MLWQKSTAPEQAPAPPRPYQGVRVKEPVKELLRRKRGHTSVGAAGPPTAGVLPHQPLATYSTVGPSCLD
MEVSASTVTEEGTLCAGWLSQPAPATLHALAPWTPYTEYVSHEAVSCPYSTDMYVQPVCPSYTVVGPSSV
LTYASPPLITNVTPRSTATPAVGPQLEGPEHQAPLTYFPWPQPLSTLPTSSLQYQPPAPTLSGPQFVQLPISIPE
PVLQDMDDPRRAISSLTIDKLLLEEEESNTYELNHTLSVEGF
>T02251
MSSAIERKSLDPSEEPVDEVLQIPPSLLTCGGCQQNIGDRYFLKAIDQYWHEDCLSCDLCGCRLGEVGRRL
YYKLGRKLCRRDYLRLFGQDGLCASCDKRIRAYEMTMRVKDKVYHLECFKCAACQKHFCVGDRYLLIN
SDIVCEQDIYEWTKINGII
>T02344
MLGAVKMEGLEPSDWSSYYAEPEGYSSVSNMNAGLGMNGMNTYMSMSAAAMGGGSGNMSAGSMNM
SSYVGAGMSPSLAGMSPGAGAMAGMSGSAGAAGVAGMGPHLSPSLSPLGGQAAGAMGGLAPYANMNS
MSPMYGQAGLSRARDPKTYRRSYTHAKPPYSYISLITMAIQQSPNKMLTLSEIYQWIMDLFPFYRQNQQR
WQNSIRHSLSFNDCFLKVPRSPDKPGKGSFWTLHPDSGNMFENGCYLRRQKRFKCEKQLALKEAAGAAS
SGGKKTAPGSQASQAQLGEAAGSASETPAGTESPHSSASPCQEHKRGGLSELKGAPASALSPPEPAPSPGQ
QQQAAAHLLGPPHHPGLPPEAHLKPEHHYAFNHPFSINNLMSSEQQHHHSHHHHQPHKMDLKAYEQVM
HYPGGYGSPMPGSLAMGPVTNKAGLDASPLAADTSYYQGVYSRPIMNSS
>T02349
MEFFISMSETIKYNDDDHKTLFLKTLNEQRLEGEFCDIAIVVEDVKFRAHRCVLAACSTYFKKLFKKLEVD
SSSVIEIDFLRSDIFEEVLNYMYTAKISVKKEDVNLMMSSGQILGIRFLDKLCSQKRDVSSPDESNGQSKSK
YCLKLNRPIGDAADAQDDDVEEIGDQDDSPSDDTVEGTPPSQEDGKSPTTTLRVQEAILKELGSEEVRKVN
CYGQEVESMETPESKDLGSQTPQALTFNDGMSEVKDEQTPGWTTAASDMKFEYLLYGHHREQIACQACG
KTFSDEGRLRKHEKLHTADRPFVCEMCTKGFTTQAHLKEHLKIHTGYKPYSCEVCGKSFIRAPDLKKHER
VHSNERPFACHMCDKAFKHKSHLKDHERRHRGEKPFVCGSCTKAFAKASDLKRHENNMHSERKQVTPS
AIQSETEQLQAAAMAAEAEQQLETIACS
>T03461
MMAYMNPGPHYSVNALALSGPNVDLMHQAVPYSSAPRKQRRERTTFTRSQLEELEALFAKTQYPDVYAR
EEVALKINLPESRVQVWFKNRRAKCRQQRQQQKQQQQPPGAQTKARPAKRKAGTSPRPSTDVCTDPLGIS
DSYSPSLPGPSGSPTTAVATVSIWSPASEAPLPEAQRAGLVASGPSLTSAPYAMTYAPASAFCSSPSAYASPSS
YFSGLDPYLSPMVPQLGGPALSPLSGPSVGPSLAQSPTSLSGQSYSTYSPVDSLEFKDPTGTWKFTYNPMD
PLDYKDQSAWKFQIL
>T04203
MAEAPQVVETDPDFEPLPRQRSCTWPLPRPEFNQSNSTTSSPAPSGGAAANPDAAASLASASAVSTDFMSN
LSLLEESEDFARAPGCVAVAAAAAASRGLCGDFQGPEAGCVHPAPPQPPPTGPLSQPPPVPPSAAAAAGPL
AGQPRKTSSSRRNAWGNLSYADLITKAIESSAEKRLTLSQIYEWMVKSVPYFKDKGDSNSSAGWKNSIRH
NLSLHSKFIRVQNEGTGKSSWWMLNPEGGKSGKSPRRRAASMDNNSKFAKSRGRAAKKKASLQSGQEG
PGDSPGSQFSKWPASPGSHSNDDFDNWSTFRPRTSSNASTISGRLSPIMTEQDDLGDGDVHSLVYPPSAAK
MASTLPSLSEISNPENMENLLDNLNLLSSPTSLTVSTQSSPGSMMQQTPCYSFAPPNTSLNSPSPNYSKYTY
GQSSMSPLPQMPMQTLQDSKSSYGGLNQYNCAPGLLKELLTSDSPPHNDIMSPVDPGVAQPNSRVLGQNV
MMGPNSVMPAYGSQASHNKMMNPSSHTHPGHAQQTASVNGRTPPHVVNTMPHTSAMNRLTPVKTPLQV
PLSHPMQMSALGRYSSVSSCNGYGRMGVLHQEKLPSDLDGMFIERLDCDMESIIRNDLMDGDTLDFNFD
NVLPNQSFPHSVKTTTHSWVSG
>T04368
MEPAVLAAHHLPHHEPISFGIDQILSGPEPPGGGLGPGQSGQSHGESAAFSSGFHGASGYAPAGSLASLPRG

SGVGPGGVIRVPAHRPLPVPPPSGAAPAVPGPSGLGGAGGLAGLTFPWMDSGRRFAKDRLTAALSPFSGTR
RIGHPYQNRTPPKRKKPRTSFSRSQVLELERRFLRQKYLASAERAALAKALRMTDAQVKTWFQNRRTKW
RRQTAEEREAERHRAGRLLLHLQQDALPRPLRPPLPPDPLCLHNSSLFALQNLQPWAEDNKVASVSGLAS
VV
>T04668
MSDTDNSAEMPARCPSPNPAPGAKQEPPNSGITISLLEIGSLPTVCYHSFPPPKNSICPVEKRGRVQKFSNLL
KDVKDVLKNIAGVEEKSTVGEPFDDAYIPEDLSELNVRGVEKKNKIRFKDDLFIHFDPEREQNTMKQMLL
KNQSAKNMVPKFARDLCNAEETRGFDGMLLSVKRPRNGSLHLRGEYRKLRNNMEQLLQEADHWSKQH
NELSELMRSYQECQNETQETTDKDRACLQNQPNNGLSTKQKLEEQVKKLSHDTHALHLIAALLENECQV
LQQRVDILKDFHLHEAGLGHEKPLQMSCEQDKKCPKLAEADKTDAFKHTTRATEGTIRKPKILRSPDVCF
TKKARNNRFNARVAKKSLVGKRRTVSSFR
>T04849
MSSQVVGIEPLYIKAEPASPDSPKGSSETETEPPVTLASGPAPARCLPGHKEEEDGEGAGSGEQGSGKLVLS
SLPKRLCLVCGDVASGYHYGVASCEACKAFFKRTIQGSIEYSCPASNECEITKRRRKACQACRFTKCLRVG
MLKEGVRLDRVRGGRQKYKRRPEVDPLPFPGPFPAGPLAVAGGPRKTAPVNALVSHLLVVEPEKLYAMPD
PASPDGHLPAVATLCDLFDREIVVTISWAKSIPGFSSLSLSDQMSVLQSVWMEVLVLGVAQGSLPLQDELAF
AEDLVLDEEGARAAGLGDLGAALQLVRRLQALRLEREEYVLLKALALANSDSVHIEDAEAVEQLREALH
EALLEYEAGRAGPGGGAERRRAGRLLLTLPLLRQTAGKVLAHFYGVKLEGKVHAQVFLEMLEAMMD
>T05012
MAAVVQQNDLVFEFASNGMEDEQQLGDPAIFPAVIVEHVPGADILNSYAGLACVEEPNDMITESSLDVAEE
EIIDDDDDDITLTVEASCHNGDETIETIEAAEALLNIDSPSPPVLDEKQINNNIFSSSEDDIVAPITHVSVTLDG
IPEVMETQQVQETNADSPGASSPEQRKRKKGRKTKPPRPDSPTTTPNISVKKKNKDGKGNTIYLWEFLLAL
LQDKATCPKYIKWTQREKGIFKLVDSKAVSRLWGKHKNKPDMNYETMGRALRYYYQRGILAKVEGQRL
VYQFKEMPKDLIYIDDEDPSSSIESSDQSLSSTTASSRNQANRSRVSSSPGIKGGAATILKPGNSKAANPKDP
VEVGQPSEVLRTVQPSQAPYPTQLFRTVHVVQPVQAVPEEATIASTMQEEAANSSVPSIRTIQASTQVPVVV
SPGNQQLHTVTVPLTTVIASIDPSSGAGSQKFILQTIPSSQPMTVLKENVMLQSQKPGSPSIVLSPTQVQQVL
TSNVQSICNGAGSVASAPSFSATTPVVTFSRSSQLVAHPPGTVITSVIKAQETKTLKQEVEKKAEDDLNEDA
EKSAQQPQPYVMVLSSSNGFSSQVAVKQNELLEPNSF
>T06593
MPRSFLVKSKKAHSYHQPRSPGPDYSLRLETVPAPGRAEGGAVSAGESKMEPRERLSPDSQLTEAPDRASA
SPNSCEGSVCDPCSEFEDFWRPPSPSVSPASEKSLCRSLDEAQPYTLPFKPYAWSGLAGSDLRHLVQSYRQC
SALERSAGLSLFCERGSEPGRPAARYGPEQAAGGAGAGQPGSCGVAGGATSAAGLGLYGDFAPAAAGLYE
RPSTAAGRLYQDHGHELHADKSVGVKVESELLCTRLLLGGGSYKCIKCSKVFSTPHGLEVHVRRSHSGTR
PFACEMCGKTFGHAVSLEQHKAVHSQERSFDCKICGKSFKRSSTLSTHLLIHSDTRPYPCQYCGKRFHQKS
DMKKHTFIHTGEKPHKCQVCGKAFSQSSNLITHSRKHTGFKPFGCDLCGKGFQRKVDLRRHRETQHGLK
>T01049
MLGAVKMEGHEPSDWSSYYAEPEGYSSVSNMNASLGMNGMNTYMSMSAAAMGSGSGNMSAGSMNMS
SYVGAGMSPSLAGMSPGAGAMAGMSGSAGAAGVAGMGPHLSPSLSPLGGQAAGAMGGLAPYANMNS
MSPMYGQAGLSRARDPKTYRRSYTHAKPPYSYISLITMAIQQSPNKMLTLSEIYQWIMDLFPFYRQNQQR
WQNSIRHSLSFNDFLKVPRAPDKPGKGSFWTLHPDSGNMFENGCYLRRQKRFKCENELALKEAAGAGSG
GGKKTAPGTQASQVQLGEAAGSASETPAGTESPHSSASPCQEHKRGGLSELKGTPASALSPPEPAPSPGQQ
QQAAAHLLGPPHHPGLPPEAHLKPEHHYAFNHPFSINNLMSSEQQHHHSHHHHQPHKMDLKTYEQVMH
YPGGYGSPMPGSLAMGPVTNKAGLDASPLAADTSYYQGVYSRPIMNSS
>T01050

MLGSVKMEAHDLAEWSYYPEAGEVYSPVNPVPTMAPLNSYMSLNPLSSPYPPGGLQASPLPTGPLAPPAP
TAPLGPTFPGLGAGSGTGGSASGYGAPGPGLVHGKEMAKGYRRPLTHAKPPYSYISLITMAIQQAPGKML
TLSEIYQWIMDLFPYYRENQQRWQNSIRHSLSFNDCFVKVARSPDKPGKGSYWALHPSSGNMFENGCYLR
RQKRFKLEEKAKKGNSATSATRNGTVGSATSATTTAATAVTSPAQPQPTPPSEPEAQSGEDVGGLDCASPPS
SAPYFTGLELPGELKLDAPYNFNHPFSINNLMSEQTSTPSKLDVGFGGYGAESGEPGVYYQSLYSRSLLNA
S
>T01921
MPRSFLVKSKKAHSYHQPRSPGPDYSLRLETVPVPGRADGGAVSAGESKMEPRERLSPESQLTEAPDRASA
SPNSCEGSVCDPSSEFEDYWRPPSPSVSPASEKSLCRSLDEAQPYTLPFKPYAWSGLAGSDLRHLVQSYRQC
SALERSAGLSLFCERGAESGRPAARYGSEQAAGGAGAGQPGSCGAASGATSAGGLGLYGDFAPAAAGLFE
RPSTAAGRLYQDRGHELHADKSVGVKVESELLCTRLLLGGGSYKCIKCSKVFSTPHGLEVHVRRSHSGTR
PFACEMCGKTFGHAVSLEQHKAVHSQERSFDCKICGKSFKRSSTLSTHLLIHSDTRPYPCQYCGKRFHQKS
DMKKHTFIHTGEKPHKCQVCGKAFSQSSNLITHSRKHTGFKPFGCDLCGKGFQRKVDLRRHRETQHGLK
>T03458
MMTYMNPGPHYSVNALALSGPSVDLMHQAVPYSSAPRKQRRERTTFTRSQLEELEALFAKTQYPDVYAR
EEVALKINLPESRVQVWFKNRRAKCRQQRQQQKQQQQPPGVQAKARPAKRKAGTSPRPSTDVCTDPLGI
SDSYSPSLPGPSGSPTTAVATVSIWSPASESPLPEAQRAGLVASGSSLTSAPYAMTYAPASAFCSSPSAYASPSS
YFSGLDPYLSPMVPQLGGPALSPLSGPSVGPSLAQSPTSLSGQSYSTYSPVDSLEFKDPTGTWKFTYNPMD
PLDYKDQSAWKFQIL
>T04761
MAMWIQAQQLQGDALHQMQALYGQHFPIEVRHYLSQWIESQAWDSIDLDNPQENIKATQLLEGLVQELQ
KKAEHQVGEDGFLLKIKLGHYATQLQNTYDRCPMELVRCIRHILYNEQRLVREANNGSSPAGSLADAMSQ
KHLQINQTFEELRLITQDTESELKKLQQTQEYFIIQYQESLRIQAQFAQLAQLNPQERMSRETALQQKQVSL
ETWLQREAQTLQQYRVELAEKHQKTLQLLRKQQTIILDDELIQWKRRQQLAGNGGPPEGSLDVLQSWCE
KLAEIIWQNRQQIRRLEHLCQQLPIPGPVEEMLAEVNATITDIISALSTSTFIIEKQPPQVLKTQTKFAATVRL
LVGGKLNVHMNPPQVKATIISEQQAKSLLKNENTRNDYSGEILNNCCVMEYHQATGTLSAHFRNMSLKRI
KRSDRRGAESVTEEKFTILFDSQFSVGGNELVFQVKTLSLPVVVIVHGSQDNNATATVLWDNAFREPGRVP
FAVPDKVLWPQLCEALNMKFKAEVQSNRGLTKENLVFLAQKLFNSSSNHLEDYNSMSVSWSQFNRENLP
GRNYTFWQWFDGVMEVLKKHLKPHWNDGAILGFVNKQQAHDLLINKPDGTFLLRFSDSEIGGITIAWKF
DSQERMFWNLMPFTTRDFSIRSLADRLGDLNYLIYVFPDRPKDEVYSKYYTPVPCERATAKAADGYVKPQ
IKQVVPEFVNASTDAGSGATYMDQAPSPVVCPQAHYNMYPQNPDSVLDTDGDFDLEDTMDVARRVEELL
GRPMDSQWIPHAQS
>T05026
MVVVAAAPSAASAAPKVLLLSGQPASGGRALPLMVPGPRAAGSEASGTPQARKRQRLTHLSPEEKALRR
KLKNRVAAQTARDRKKARMSELEQQVVDLEEENQKLQLENQLLREKTHGLVIENQELRTRLGMNALVTE
EVSEAESKGNGVRLVAGSAESAALRLRAPLQQVQAQLSPPQNIFPWILTLLPLQILSLISFWAFWTSWTLSC
FSNVLPQSLLIWRNSQRSTQKDLVPYQPPFLCQWGPHQPSWKPLMNSFVLTMYTPSL
>T00646
MVHSSMGAPEIRMSKPLEAEKQGLDSPSEHTDTERNGPDTNHQNPQNKTSPFSVSPTGPSTKIKAEDPSGD
SAPAAPLPPQPAQPHLPQAQLMLTGSQLAGDIQQLLQLQQLVLVPGHHLQPPAQFLLPQAQQSQPGLLPTP
NLFQLPQQTQGALLTSQPRAGLPTQPPKCLEPPSHPEEPSDLEELEQFARTFKQRRIKLGFTQGDVGLAMG
KLYGNDFSQTTISRFEALNLSFKNMCKLKPLLEKWLNDAETMSVDSSLPSPNQLSSPSLGFDGLPGRRRKK
RTSIETNVRFALEKSFLANQKPTSEEILLIAEQLHMEKEVIRVWFCNRRQKEKRINPCSAAPMLPSPGKPAS
YSPHMVTPQGGAGTLPLSQASSSLSTTVTTLSSAVGTLHPSRTAGGGGGGGGAAPPLNSIPSVTPPPPATTN

STNPSPQGSHSAIGLSGLNPSTGPGLWWNPAPYQP
>T00899
MGSDVRDLNALLPAVPSLGGGGGCALPVSGAAQWAPVLDFAPPGASAYGSLGGPAPPPAPPPPPPPPPHSFI
KQEPSWGGAEPHEEQCLSAFTVHFSGQFTGTAGACRYGPFGPPPPSQASSGQARMFPNAPYLPSCLESQPA
IRNQGYSTVTFDGTPSYGHTPSHHAAQFPNHSFKHEDPMGQQGSLGEQQYSVPPPVYGCHTPTDSCTGSQ
ALLLRTPYSSDNLYQMTSQLECMTWNQMNLGATLKGVAAGSSSSVKWTEGQSNHSTGYESDNHTTPILC
GAQYRIHTHGVFRGIQDVRRVPGVAPTLVRSASETSEKRPFMCAYPGCNKRYFKLSHLQMHSRKHTGEKP
YQCDFKDCERRFSRSDQLKRHQRRHTGVKPFQCKTCQRKFSRSDHLKTHTRTHTGKTSEKPFSCRWPSCQ
KKFARSDELVRHHNMHQRNMTKLQLAL
>T00900
MGSDVRDLNALLPAVPSLGGGGGCALPVSGAAQWAPVLDFAPPGASAYGSLGGPAPPPAPPPPPPPPPHSFI
KQEPSWGGAEPHEEQCLSAFTVHFSGQFTGTAGACRYGPFGPPPPSQASSGQARMFPNAPYLPSCLESQPA
IRNQGYSTVTFDGTPSYGHTPSHHAAQFPNHSFKHEDPMGQQGSLGEQQYSVPPPVYGCHTPTDSCTGSQ
ALLLRTPYSSDNLYQMTSQLECMTWNQMNLGATLKGHSTGYESDNHTTPILCGAQYRIHTHGVFRGIQD
VRRVPGVAPTLVRSASETSEKRPFMCAYPGCNKRYFKLSHLQMHSRKHTGEKPYQCDFKDCERRFSRSDQ
LKRHQRRHTGVKPFQCKTCQRKFSRSDHLKTHTRTHTGEKPFSCRWPSCQKKFARSDELVRHHNMHQRN
MTKLQLAL
>T01839
MGSDVRDLNALLPAVPSLGGGGGCALPVSGAAQWAPVLDFAPPGASAYGSLGGPAPPPAPPPPPPPPPHSFI
KQEPSWGGAEPHEEQCLSAFTVHFSGQFTGTAGACRYGPFGPPPPSQASSGQARMFPNAPYLPSCLESQPA
IRNQGYSTVTFDGTPSYGHTPSHHAAQFPNHSFKHEDPMGQQGSLGEQQYSVPPPVYGCHTPTDSCTGSQ
ALLLRTPYSSDNLYQMTSQLECMTWNQMNLGATLKGVAAGSSSSVKWTEGQSNHSTGYESDNHTTPILC
GAQYRIHTHGVFRGIQDVRRVPGVAPTLVRSASETSEKRPFMCAYPGCNKRYFKLSHLQMHSRKHTGEKP
YQCDFKDCERRFSRSDQLKRHQRRHTGVKPFQCKTCQRKFSRSDHLKTHTRTHTGEKPFSCRWPSCQKKF
ARSDELVRHHNMHQRNMTKLQLAL
>T01840
MGSDVRDLNALLPAVPSLGGGGGCALPVSGAAQWAPVLDFAPPGASAYGSLGGPAPPPAPPPPPPPPPHSFI
KQEPSWGGAEPHEEQCLSAFTVHFSGQFTGTAGACRYGPFGPPPPSQASSGQARMFPNAPYLPSCLESQPA
IRNQGYSTVTFDGTPSYGHTPSHHAAQFPNHSFKHEDPMGQQGSLGEQQYSVPPPVYGCHTPTDSCTGSQ
ALLLRTPYSSDNLYQMTSQLECMTWNQMNLGATLKGHSTGYESDNHTTPILCGAQYRIHTHGVFRGIQD
VRRVPGVAPTLVRSASETSEKRPFMCAYPGCNKRYFKLSHLQMHSRKHTGEKPYQCDFKDCERRFSRSDQ
LKRHQRRHTGVKPFQCKTCQRKFSRSDHLKTHTRTHTGKTSEKPFSCRWPSCQKKFARSDELVRHHNMH
QRNMTKLQLAL
>T02054
MEHLGPHHLHPGHAEPISFGIDQILNSPDQGGCMGPASRLQDGEYGLGCLVGGAYTYGGGGSAAATGAG
GAGAYGTGGPGGPGGPAGGGGACSMGPLTGSYNVNMALAGGPGPGGGGGSSGGAGALSAAGVIRVPAH
RPLAGAVAHPQPLATGLPTVPSVPAMPGVNNLTGLTFPWMESNRRYTKDRFTGHPYQNRTPPKKKKPRTSF
TRLQICELEKRFHRQKYLASAERAALAKALKMTDAQVKTWFQNRRTKWRRQTAEEREAERQQANRILL
QLQQEAFQKSLAQPLPADPLCVHNSSLFALQNLQPWSDDSTKITSVTSVASACE
>T05887
MPRGFLVKRSKKSTPVSYRVRGGEDGDRALLLSPSCGGARAEPPAPSPVPGPLPPPPPAERAHAALAAALA
CAPGPQPPPQGPRAAHFGNPEAAHPAPLYSPTRPVSREHEKHKYFERSFNLGSPVSAESFPTPAALLGGGG
GGGASGAGGGGTCGGDPLLFAPAELKMGTAFSAGAEAARGPGPGPPLPPAAALRPPGKRPPPPTAAEPPA
KAVKAPGAKKPKAIRKLHFEDEVTTSPVLGLKIKEGPVEAPRGRAGGAARPLGEFICQLCKEEYADPFALA

QHKCSRIVRVEYRCPECAKVFSCPANLASHRRWHKPRPAPAAARAPEPEAAARAEAREAPGGGSDRDTPS
PGGVSESGSEDGLYECHHCAKKFRRQAYLRKHLLAHHQALQAKGAPLAPPAEDLLALYPGPDEKAPQEA
AGDGEGAGVLGLSASAECHLCPVCGESFASKGAQERHLRLLHAAQVFPCKYCPATFYSSPGLTRHINKCH
PSENRQVILLQVPVRPAC
>T08251
MAGGVDGPIGIPFPDHSSDILSGLNEQRTQGLLCDVVILVEGREFPTHRSVLAACSQYFKKLFTSGAVVDQ
QNVYEIDFVSAEALTALMDFAYTATLTVSTANVGDILSAARLLEIPAVSHVCADLLDRQILAADAGADAGQ
LDLVDQIDQRNLLRAKEYLEFFQSNPMNSLPPAAAAAAASFPWSAFGASDDDLDATKEAVAAAVAAVAAG
DCNGLDFYGPGPPAERPPTGDGDEGDSNPGLWPERDEDAPTGGLFPPPVAPPAATQNGHYGRGGEEEAAS
LSEAAPEPGDSPGFLSGAAEGEDGDGPDVDGLAASTLLQQMMSSVGRAGAAAGDSDEESRADDKGVMD
YYLKYFSGAHDGDVYPAWSQKVEKKIRAKAFQKCPICEKVIQGAGKLPRHIRTHTGEKPYECNICKVRFT
RQDKLKVHMRKHTGEKPYLCQQCGAAFAHNYDLKNHMRVHTGLRPYQCDSCCKTFVRSDHLHRHLKK
DGCNGVPSRRGRKPRVRGGAPDPSPGATATPGAPAQPSSPDARRNGQEKHFKDEDEDEDVASPDGLGRLN
VAGAGGGGDSGGGPGAATDGNFTAGLA
>T01737
MSSYFVNSLFSKYKTGESLRPNYYDCGFAQDLGGRPTVVYGPSSGGSFQHPSQIQEFYHGPSSLSTAPYQQ
NPCAVACHGDPGNFYGYDPLQRQSLFGAQDPDLVQYADCKLAAASGLGEEAEGSEQSPSPTQLFPWMRP
QAAAGRRRGRQTYSRYQTLELEKEFLFNPYLTRKRRIEVSHALGLTERQVKIWFQNRRMKWKKENNKDK
FPSSKCEQEELEKEKLERAPETAEQGDAQKGDKK
>T02083
MMSYLKQPPYAVNGLSLTTSGMDLLHPSVGYPATPRKQRRERTTFTRAQLDVLEALFAKTRYPDIFMREEV
ALKINLPESRVQVWFKNRRAKCRQQQQQQQNGGQNKVRPAKKKSSPAREVSSESGTSGQFTPPSSTSVPTI
ASSSAPVSIWSPASISPLSDPLSTSSSCMQRSYPMTYTQASGYSQGYAGSTSYFGGMDCGSYLTPMHHQLP
GPGATLSPMGTNAVTSHLNQSPASLSTQGYGASSLGFNSTTDCLDYKDQTASWKLNFNADCLDYKDQTSS
WKFQVL
>T02450
MRQPPGESDMAVSDALLPSFSTFASGPAGREKTLRPAGAPTNRWREELSHMKRLPPLPGRPYDLAATVATD
LESGGAGAACSSNNPALLARRETEEFNDLLDLDFILSNSLTHQESVAATVTTSASASSSSSPASSGPASAPST
CSFSYPIRAGGDPGVAASNTGGGLLYSRESAPPPTAPFNLADINDVSPSGGFVAELLRPELDPVYIPPQQPQP
PGGGLMGKFVLKASLTTPGSEYSSPSVISVSKGSPDGSHPVVVAPYSGGPPRMCPKIKQEAVPSCTVSRSLE
AHLSAGPQLSNGHRPNTHDFPLGRQLPTRTTPTLSPEELLNSRDCHPGLPLPPGFHPHPGPNYPPFLPDQMQ
SQVPSLHYQELMPPGSCLPEEPKPKRGRRSWPRKRTATHTCDYAGCGKTYTKSSHLKAHLRTHTGEKPYH
CDWDGCGWKFARSDELTRHYRKHTGHRPFQCQKCDRAFSRSDHLALHMKRHF
>T05840
ALDVDGGGGGGGHGEYLQQQQQQQQHGNGAAAAAAQTGDLASAQLGGAPNRWEVLSATPTTIKDEA
GNLVQIPGAATSSGQYVLPLQNLQNQQIFSVAPGSDSSNGTVSNVQYQVIPQIQSTDAQQVQIGFTGSSDN
GGINQENSQIQIIPGSNQTLLASGTPPANIQNLIPQTGQVQVQGVAIGGSSFPGQTQVVANVPLGLPGNITFV
PINSVDLDSLGLSGSSQTMTAGINADGHLINTGQAMDSSDNSERTGERVSPDVNETNADTDLFVPTSSSSQ
LPVTIDSTGILQQNTNSLTTTSGQVHSSDLQGNYIQSPVSEETQAQNIQVSTAQPVVQHLQLQDSQQPTSQA
QIVQGITPQTIHGVQASGQNISQQALQNLQLQLNPGTFLIQAQTVTPSGQITWQTFQVQGVQNLQNLQIQN
TAAQQITLTPVQTLTLGQVAAGGALTSTPVSLSTGQLPNLQTVTVNSIDSTGIQLHPGENADSPADIRIKEEE
PDPEEWQLSGDSTLNTNDLTHLRVQVVDEEGDQQHQEGKRLRRVACTCPNCKEGGGRGTNLGKKKQHIC
HIPGCGKVYGKTSHLRAHLRWHSGERPFICNWMFCGKRFTRSDELQRHRRTHTGEKKFVCPECSKRFMR
SDHLAKHIKTHQNKKVIHSSSTVLASVEAGRDDALITAGGTTLILANIQQGSVSGIGTVNTSATSNQDILTN

TEIPLQLVTVSGNETME

>T05943

MMTPQVITPQQMQQILQQQVLSPQQLQVLLQQQQALMLQQQLQEFYKKQQEQLQLQLLQQQHAGKQP
KEQQVATQQLAFQQQLLQMQQLQQQHLLSLQRQGLLTIQPGQPALPLQPLAQGMIPTELQQLWKEVTSAH
TAEETTSSNHSSLDLTSTCVSSSAPSKSSLIMNPHASTNGQLSVHTPKRESLSHEEHPHSHPLYGHGVCKWP
GCEAVCDDFPAFLKHLNSEHALDDRSTAQCRVQMQVVQQLELQLAKDKERLQAMMTHLHVKSTEPKAA
PQPLNLVSSVTLSKSASEASPQSLPHTPTTPTAPLTPVTQGPSVITTTSMHTVGPIRRRYSDKYNVPISSADIA
QNQEFYKNAEVRPPFTYASLIRQAILESPEKQLTLNEIYNWFTRMFAYFRRNAATWKNAVRHNLSLHKCFV
RVENVKGAVWTVDEVEFQKRRPQKISGNPSLIKNMQSSHAYCTPLNAALQASMAENSIPLYTTASMGNPT
LGSLASAIREELNGAMEHTNSNESDSSPGRSPMQAVHPIHVKEEPLDPEEAEGPLSLVTTANHSPDFDHDR
DYEDEPVNEDME

>T06029

MSSPDAGYASDDQSQPRSAQPAVMAGLGPCPWAESLSPLGDVKVKGEVVASSGAPAGTSGRAKAESRIRR
PMNAFMVWAKDERKRLAQQNPDLHNAELSKMLGKSWKALTLAEKRPFVEEAERLRVQHMQDHPNYKY
RPRRRKQVKRMKRVEGGFLHALVEPQAGALGPEGGRVAMDGLGLPFPEPGYPAGPPLMSPHMGPHYRDC
QGLGAPALDGYPLPTPDTSPLDGVEQDPAFFAAPLPGDCPAAGTYTYAPVSDYAVSVEPPAGPMRVGPDPS
GPAMPGILAPPSALHLYYGAMGSPAASAGRGFHAQPQQPLQPQAPPPPPQQQHPAHGPGQPSPPPEALPCR
DGTESNQPTELLGEVDRTEFEQYLPFVYKPEMGLPYQGHDCGVNLSDSHGAISSVVSDASSAVYYCNYPD
I
