# Supplementary material for: Towards biological characters of interactions between transcription factors and their DNA targets in mammals
Source: BMC Genomics. 2012 Aug 13;13:388. doi: 10.1186/1471-2164-13-388 (PMC3472306; doi:10.1186/1471-2164-13-388)
Supplement: Additional file 4 — Information of 38 conformational and physicochemical attributes. [file 1471-2164-13-388-S4.pdf]

Detailed information of 38 conformational and physicochemical attributes

Attribute id	Attribute name	Attribute type	origin for attribute	method for attribute definition
P0000001	Twist	Conformational	B-DNA	difined by Sklenar,Ponomarenko
P0000002	Rise	Conformational	B-DNA	difined by Sklenar,Ponomarenko
P0000003	Bend	Conformational	B-DNA	difined by Sklenar,Ponomarenko
P0000004	Tip	Conformational	B-DNA	difined by Sklenar,Ponomarenko
P0000005	Inclination	Conformational	B-DNA	difined by Sklenar,Ponomarenko
P0000006	Major groove width	Conformational	B-DNA	difined by Sklenar,Ponomarenko
P0000007	Major groove depth	Conformational	B-DNA	difined by Sklenar,Ponomarenko
P0000008	Minor groove width	Conformational	B-DNA	difined by Sklenar,Ponomarenko
P0000009	Minor groove depth	Conformational	B-DNA	difined by Sklenar,Ponomarenko
P0000010	Roll	Conformational	Free DNA	Averaged for X-rays
P0000011	Twist	Conformational	Free DNA	Averaged for X-rays
P0000012	Tilt	Conformational	Free DNA	Averaged for X-rays
P0000013	Slide	Conformational	Free DNA	Averaged for X-rays
P0000014	Roll	Conformational	DNA/protein-complex	Averaged for X-rays
P0000015	Twist	Conformational	DNA/protein-complex	Averaged for X-rays
P0000016	Tilt	Conformational	DNA/protein-complex	Averaged for X-rays
P0000017	Slide	Conformational	DNA/protein-complex	Averaged for X-rays
P0000018	Twist	Conformational	B-DNA	Regressed for X-rays
P0000019	Wedge	Conformational	B-DNA	Regressed for X-rays
P0000020	Direction	Conformational	B-DNA	Regressed for X-rays
P0000021	Persistence length	Physico-chemical	B-DNA	Experimental
P0000022	Melting temperature	Physico-chemical	B-DNA	Experimental
P0000023	Probability of contacting with nucleosome core	Physico-chemical	B-DNA	Experimental
P0000024	Mobility to bend towards major groove	Physico-chemical	B-DNA	Experimental
P0000025	Mobility to bend towords minor groove	Physico-chemical	B-DNA	Experimental
P0000026	Twist	Conformational	B-DNA	Averaged for the X-ray centers
P0000027	Tilt	Conformational	B-DNA	Averaged for the X-ray centers
P0000028	Roll	Conformational	B-DNA	Averaged for the X-ray centers
P0000029	Slide	Conformational	B-DNA	Averaged for the X-ray centers
P0000030	Propeller	Conformational	B-DNA	Averaged for the X-ray centers
P0000031	Minor groove size	Conformational	B-DNA	Averaged for the X-ray centers
P0000032	Minor groove dist	Conformational	B-DNA	Averaged for the X-ray centers
P0000033	Major groove size	Conformational	B-DNA	Averaged for the X-ray centers
P0000034	Major groove dist	Conformational	B-DNA	Averaged for the X-ray centers
P0000035	Clash strength	Physico-chemical	B-DNA	Averaged for the X-ray centers
P0000036	Enthalpy change	Physico-chemical	B-DNA	Calculated
P0000037	Entropy change	Physico-chemical	B-DNA	Calculated
P0000038	Free energy change	Physico-chemical	B-DNA	Calculated

Empirical values of 16 binucleotides combination for 38 conformational and physicochemical attributes

dinucleotides	P0000001	P0000002	P0000003	P0000004	P0000005	P0000006	P0000007	P0000008	P0000009	P0000010
AA	38.90	3.16	3.07	1.76	-1.43	12.15	9.12	5.30	9.03	0.30
AT	33.81	3.89	2.60	1.87	0.00	12.87	8.96	5.31	8.91	-0.80
AG	32.15	3.63	2.31	0.90	-0.92	13.51	8.96	5.19	8.98	4.50
AC	31.12	3.41	2.97	2.00	-0.11	12.37	9.41	6.04	8.79	0.50
TA	33.28	3.21	6.74	6.70	0.00	12.32	9.60	6.40	9.00	2.80
TT	38.90	3.16	3.07	1.76	1.43	12.15	9.12	5.30	9.03	0.30
TG	41.41	3.23	3.58	-1.64	-1.31	13.58	8.67	4.79	9.09	0.50
TC	41.31	3.47	2.51	1.35	0.33	13.93	8.76	4.71	9.11	-1.30
GA	41.31	3.47	2.51	1.35	-0.33	13.93	8.76	4.71	9.11	-1.30
GT	31.12	3.41	2.97	2.00	0.11	12.37	9.41	6.04	8.79	0.50
GG	34.96	4.08	2.16	0.71	1.11	15.49	8.45	4.62	8.99	6.00
GC	38.50	3.81	3.06	2.50	0.00	14.55	8.67	4.74	8.98	-6.20
CA	41.41	3.23	3.58	-1.64	1.31	13.58	8.67	4.79	9.09	0.50
CT	32.15	3.63	2.31	0.90	0.92	13.51	8.96	5.19	8.98	4.50
CG	32.91	3.60	2.81	0.22	0.00	14.42	8.81	5.16	9.06	-6.20
CC	34.96	4.08	2.16	0.71	-1.11	15.49	8.45	4.62	8.99	6.00

dinucleotides	P0000011	P0000012	P0000013	P0000014	P0000015	P0000016	P0000017	P0000018	P0000019	P0000020
AA	35.30	0.50	-0.10	0.80	35.60	1.90	0.10	35.62	7.20	-154.00
AT	31.20	0.00	-0.40	0.00	29.30	0.00	-0.70	31.50	2.60	0.00
AG	31.20	2.80	0.40	5.60	31.90	1.30	-0.30	27.70	8.40	2.00
AC	32.60	0.10	-0.20	-0.20	31.10	0.30	-0.60	34.40	1.10	143.00
TA	40.50	0.00	0.90	2.70	39.50	0.00	0.10	36.00	0.90	0.00
TT	35.30	0.50	-0.10	0.80	35.60	1.90	0.10	35.62	7.20	154.00
TG	32.60	-0.70	1.60	6.40	36.00	0.30	0.40	34.50	3.50	64.00
TC	40.30	0.90	0.00	2.40	35.90	1.70	0.10	36.90	5.30	-120.00
GA	40.30	0.90	0.00	2.40	35.90	1.70	0.10	36.90	5.30	120.00
GT	32.60	0.10	-0.20	-0.20	31.10	-0.10	-0.60	34.40	1.10	-143.00
GG	33.30	2.70	0.80	3.30	33.30	1.00	-0.10	33.67	2.10	57.00
GC	37.30	0.00	0.40	-2.00	34.60	0.00	-0.30	40.00	5.00	180.00
CA	39.20	-0.70	1.60	6.40	35.90	0.30	0.40	34.50	3.50	-64.00
CT	31.20	2.80	0.40	5.60	31.90	1.30	-0.30	27.70	8.40	-2.00
CG	36.60	0.00	0.70	6.50	34.90	0.00	0.70	29.80	6.70	0.00
CC	33.30	2.70	0.80	3.30	33.30	1.00	-0.10	33.67	2.10	-57.00

dinucleotides	P0000021	P0000022	P0000023	P0000024	P0000025	P0000026	P0000027	P0000028	P0000029	P0000030
AA	35.00	54.50	18.40	1.18	1.04	35.80	-0.40	0.50	-0.03	-17.30
AT	20.00	57.02	7.20	1.12	1.02	33.40	0.00	-0.60	-0.37	-16.90
AG	60.00	58.42	14.50	1.06	1.09	30.50	-2.60	2.90	0.47	-14.30
AC	60.00	97.73	10.20	1.06	1.10	35.80	-0.90	0.40	-0.13	-6.70
TA	20.00	36.73	6.20	1.07	1.05	40.00	0.00	2.60	0.74	-11.10
TT	35.00	54.50	18.40	1.09	1.04	35.80	-0.40	0.50	-0.03	-17.30
TG	60.00	54.71	15.70	1.03	1.23	36.90	0.60	1.10	1.46	-8.60
TC	60.00	86.44	11.30	1.03	1.20	39.30	-0.40	-0.10	-0.07	-15.10
GA	60.00	86.44	11.30	1.08	1.12	39.30	-0.40	-0.10	-0.07	-15.10
GT	60.00	97.73	10.20	1.02	1.11	35.80	-0.90	0.40	-0.13	-6.70
GG	130.00	85.97	10.20	1.00	1.25	33.40	-1.10	6.50	0.60	-12.80
GC	85.00	136.12	5.20	0.98	1.17	38.30	0.00	-7.00	0.29	-11.70
CA	60.00	54.71	15.70	1.06	1.16	36.90	0.60	1.10	1.46	-8.60
CT	60.00	58.42	14.50	1.04	1.16	30.50	-2.60	2.90	0.47	-14.30
CG	85.00	72.55	1.10	1.02	1.25	31.10	0.00	6.60	0.63	-11.20
CC	130.00	85.97	10.20	0.99	1.27	34.30	-1.10	6.50	0.60	-12.80

dinucleotides	P0000031	P0000032	P0000033	P0000034	P0000035	P0000036	P0000037	P0000038
AA	2.98	2.94	3.98	3.38	0.64	-8.00	-21.90	-1.20
AT	3.26	4.20	4.70	3.02	1.68	-5.60	-15.20	-0.90
AG	3.98	2.79	4.70	3.36	2.53	-6.60	-16.40	-1.50
AC	3.26	4.22	3.98	3.03	0.95	-9.40	-25.50	-1.50
TA	2.70	2.97	3.26	3.81	0.00	-6.60	-18.40	-0.90
TT	2.98	2.94	3.98	3.38	0.64	-8.00	-21.90	-1.20
TG	3.70	3.09	3.98	3.79	0.80	-8.20	-21.00	-1.70
TC	2.98	2.95	3.26	3.40	0.03	-8.80	-23.50	-1.50
GA	2.98	2.95	3.26	3.40	0.03	-8.80	-23.50	-1.50
GT	3.26	4.22	3.98	3.03	0.95	-9.40	-25.50	-1.50
GG	3.98	2.80	3.98	3.38	1.78	-10.90	-28.40	-2.10
GC	3.26	4.24	3.26	3.04	0.22	-10.50	-26.40	-2.30
CA	3.70	3.09	3.98	3.79	0.80	-8.20	-21.00	-1.70
CT	3.98	2.79	4.70	3.36	2.53	-6.60	-16.40	-1.50
CG	4.70	3.21	4.70	3.77	2.42	-11.80	-29.00	-2.80
CC	3.98	2.80	3.98	3.38	1.78	-10.90	-28.40	-2.10
